# Supplementary material for: Assessing the reliability of phone surveys to measure reproductive, maternal and child health knowledge among pregnant women in rural India: a feasibility study
Source: BMJ Open. 2022 Mar 10;12(3):e056076. doi: 10.1136/bmjopen-2021-056076 (PMC8915337; doi:10.1136/bmjopen-2021-056076)
Supplement: Supplementary data [file bmjopen-2021-056076supp001.pdf]

Supplementary Table 1: Questions included in each domain of knowledge

Questions	Answer options	Correct answer(s)
**Maternal nutrition and pregnancy danger signs (6 questions)**		Maximum possible score: 6
What foods should you eat during pregnancy?	Unprompted free list, enumerator noted any correct answers mentioned	Milk/dairy products; fruits; green leafy vegetables; pulses and nuts; egg; meat; fish
What should you not eat or drink during your pregnancy?	Unprompted free list, enumerator noted any correct answers mentioned	Tea; coffee; cigarettes/bidis; alcohol
What should you do if you are anemic?	Unprompted free list, enumerator noted any correct answers mentioned	Have your blood checked regularly; take IFA tablets daily; eat well
What are the benefits of taking Iron Folic Acid tablets during pregnancy?	Unprompted free list, enumerator noted any correct answers mentioned	Help prevent/treat anemia; improve the health/well-being of my baby
How many tetanus injections should you have during pregnancy?	Number or don't know	2
What danger signs during pregnancy and before labor starts would lead you to go to the health facility immediately?	Unprompted free list, enumerator noted any correct answers mentioned	Vaginal bleeding; vaginal discharge; convulsions; stomach cramps; swelling on limbs and face; yellowing of skin; fever; decreased or absent fetal movements
**Immediate post-birth care for the neonate (4 questions)**		Maximum possible score: 4
How soon after delivery should your baby be bathed?		
How should you keep the baby warm after delivery?	Unprompted free list, enumerator noted any correct answers mentioned	
What should be used to cut the umbilical cord?	Unprompted free list, enumerator noted any correct answers mentioned	
What should be put on the cord after delivery?	Unprompted free list, enumerator noted any correct answers mentioned	
**Infant and young child feeding (5 questions)**		Maximum possible score: 5
What are some of the benefits of breastfeeding?	Unprompted free list, enumerator noted any correct answers mentioned	Promotes mother-baby bonding; helps build immunity for child; promotes child growth, wellness;<br>Reduces expenditure on medical care as child will fall sick less; the more the child breastfeeds, the more milk will be produced; it helps to maintain space between two children
How soon after delivery should you breastfeed your child?	Enumerator noted "immediately", number of hours or days mentioned, or "don't know"	Immediately or <1 hour

Questions	Answer options	Correct answer(s)
How many times per day should newborn babies be breastfed?	Enumerator can note if respondent provides either of the correct answers or "don't know"	9-10 times in 24 hours; On-demand (as often as the baby wants to feed)
How soon after delivery should you give foods or liquids other than mother's milk?		Six months
What types of foods should a baby be given after 6 months of age?	Unprompted free list, enumerator noted any correct answers mentioned	
**Infant and young child care (5 questions)**		Maximum possible score: 5
What danger signs do you know about for the newborn after delivery that would cause you to take the baby to the health facility immediately?	Unprompted free list, enumerator noted any correct answers mentioned	
How soon after your baby is born should it receive its first vaccination?		
What should you give your child to treat diarrhea?	Unprompted free list, enumerator noted any correct answers mentioned	
What are things you can do to prevent your child from getting diarrhea?	Unprompted free list, enumerator noted any correct answers mentioned	
What are three critical times for a woman to wash her hands?	Unprompted free list, enumerator noted any correct answers mentioned	
**Family planning (9 questions)**		Maximum possible score: 9
How soon after you give birth can you get pregnant again?		
What is the recommended length of time you should wait between having this child and getting pregnant with another child?		
What are benefits of using family planning?	Unprompted free list, enumerator noted any correct answers mentioned	
Which ways or methods contraception have you heard about?	Unprompted free list, enumerator noted any correct answers mentioned	
True/False: There are many safe methods of birth control	True / false / don't know	True
True/False: Female sterilization can be done at the time of giving birth	True / false / don't know	True
True/False: Male sterilization is an easy way to control family size	True / false / don't know	True
True/False: Men become physically weak after accepting male sterilization	True / false / don't know	False
True/False: PPIUCD insertion and female sterilization services can be free of cost at government facilities	True / false / don't know	True

Supplementary Table 2: Factors associated with pregnant women's RMNCH&N knowledge by domain in four districts of Madhya Pradesh, India

Variable	Mean composite score	Maternal nutrition and pregnancy danger signs	Family Planning	Infant and young child care	Immediate post-birth care for newborns	Infant and young child feeding
**Age**						
18-24	Reference					
25-34	1.91(1.35-2.46)*	1.95(1.02-2.87)*	1.92(1.18-2.67)*	1.85(1.11-2.58)*	2.32(1.1-3.54)*	1.51(0.55-2.47)*
35+	2.04(0.34-3.74)*	2.19(-0.52-4.9)	4.67(2.06-7.29)*	0.81(-1.33-2.95)	1.53(-2.42-5.47)	1.01(-2.36-4.38)
**Parity (>=1 child)**	4.39(3.74-5.03)*	3.29(2.33-4.26)*	1.74(0.92-2.56)*	5.93(5.13-6.74)*	6.8(5.46-8.14)*	4.18(3.11-5.25)*
**Caste**						
General	Reference					
Other backwards	0.62(-0.06-1.3)	0.51(-0.63-1.65)	0.07(-0.83-0.96)	0.82(-0.04-1.68)	1.94(0.47-3.41)*	-0.25(-1.41-0.91)
Scheduled	0.24(-0.58-1.06)	0.12(-1.2-1.43)	0.35(-0.71-1.4)	-0.04(-1.05-0.96)	1.08(-0.63-2.79)	-0.31(-1.65-1.04)
**Wealth Index**						
Q1	Reference					
Q2	0.32(-0.49-1.13)	-0.61(-1.99-0.78)	1.33(0.23-2.43)*	0.67(-0.4-1.75)	0.66(-1.01-2.33)	-0.47(-1.85-0.9)
Q3	0.14(-0.78-1.07)	0.17(-1.37-1.71)	1.17(-0.04-2.37)	0.5(-0.69-1.7)	-1.24(-3.06-0.57)	0.11(-1.44-1.66)
Q4	0.16(-0.83-1.16)	-0.02(-1.65-1.61)	0.95(-0.33-2.24)	0.85(-0.41-2.1)	-2.32(-4.28--0.36)*	1.35(-0.3-2.99)
Q5	0.72(-0.38-1.82)	1.8(-0.01-3.6)	2.32(0.93-3.72)*	1.21(-0.19-2.62)	-2.95(-5.22--0.68)*	1.24(-0.55-3.03)
**Education**						
No School	Reference					
Primary or Less	0.65(-0.35-1.65)	1.87(0.24-3.5)*	1.57(0.2-2.95)*	0.01(-1.34-1.36)	-1.55(-3.6-0.5)	1.35(-0.43-3.13)
Middle School	0.6(-0.51-1.72)	1.19(-0.56-2.93)	2.43(0.92-3.94)*	0.14(-1.27-1.54)	-1.78(-4.06-0.51)	1.04(-0.83-2.91)
Higher Education	1.65(0.42-2.89)*	3.42(1.51-5.34)*	3.29(1.63-4.94)*	1.5(-0.03-3.03)	-2.82(-5.41--0.24)*	2.87(0.83-4.91)*
**Literacy**						
Cannot read at all	Reference					
Reading only parts of sentence	0.95(0.01-1.9)*	0.93(-0.57-2.42)	0.88(-0.42-2.18)	0.91(-0.33-2.15)	0.33(-1.64-2.31)	1.7(0.05-3.34)*
Read whole sentence	2.29(1.42-3.15)*	3.12(1.78-4.46)*	2.29(1.17-3.41)*	1.4(0.33-2.47)*	1.86(0.11-3.61)*	2.76(1.32-4.19)*
**Self Help Group**	0.4(-0.42-1.22)	0.88(-0.57-2.34)	0.77(-0.46-2.01)	0.45(-0.71-1.61)	-0.18(-2.33-1.98)	0.08(-1.39-1.54)
**Nuclear Family**	-2.14(-2.95--1.33)*	-2.61(-3.83--1.38)*	-1.7(-2.65--0.75)*	-4.38(-5.39--3.38)*	-1.46(-2.92-0)	-0.57(-1.81-0.67)

**Primary decision maker in health decisions**	1.59(1-2.19)*	2.74(1.75-3.74)*	0.48(-0.32-1.27)	2.61(1.88-3.34)*	0.43(-0.8-1.66)	1.71(0.68-2.74)*
**Number of ANC Visits**						
1	Reference					
2	1.03(0.16-1.9)*	4.19(2.8-5.59)*	0.62(-0.62-1.86)	-0.03(-1.15-1.08)	0.18(-1.59-1.95)	0.17(-1.24-1.58)
3	1.95(1.04-2.85)*	5.91(4.37-7.45)*	0.35(-0.94-1.63)	0.65(-0.51-1.81)	1.38(-0.49-3.25)	1.44(-0.05-2.93)
4	1.68(0.81-2.54)*	6.61(5.13-8.1)*	0.19(-1.01-1.4)	0.43(-0.67-1.54)	0.12(-1.67-1.9)	1.02(-0.42-2.46)
**Satisfied with the services provided by the ASHA**	2.18(1.57-2.79)*	2.78(1.83-3.72)*	2.06(1.24-2.87)*	1.7(0.9-2.5)*	2.93(1.72-4.13)*	1.44(0.42-2.46)*
**Phone Ownership**	0.61(-0.01-1.22)	0.82(-0.2-1.85)	0.71(-0.11-1.52)	0.73(-0.1-1.57)	0.4(-0.82-1.63)	0.37(-0.67-1.4)
**District**						
Hoshangabad	Reference					
Mandsaur	-3.3(-4.51--2.09)*	-1.72(-3.54-0.1)	-1.49(-2.98-0)	-2.14(-3.59--0.7)*	-6.98(-9.75--4.21)*	-4.18(-5.98--2.38)*
Rewa	-0.13(-1.21-0.95)	-3.05(-4.78--1.31)*	-0.85(-2.24-0.54)	-0.69(-2.02-0.63)	3.58(1.18-5.98)*	0.36(-1.31-2.03)
Rajgarh	-3.05(-4.21--1.89)*	-6.59(-8.41--4.78)*	-1.26(-2.76-0.24)	-0.26(-1.68-1.16)	-4.35(-6.92--1.78)*	-2.77(-4.53--1.01)*

*= Significant at 0.05 level
