# Supplementary material for: Assessing the reliability of phone surveys to measure reproductive, maternal and child health knowledge among pregnant women in rural India: a feasibility study
Source: BMJ Open. 2022 Mar 10;12(3):e056076. doi: 10.1136/bmjopen-2021-056076 (PMC8915337; doi:10.1136/bmjopen-2021-056076)
Supplement: Author's manuscript [file bmjopen-2021-056076.draft_revisions.pdf]

BMJ Open

BMJ Open is committed to open peer review. As part of this commitment we make the peer review history of every article we publish publicly available.

When an article is published we post the peer reviewers' comments and the authors' responses online. We also post the versions of the paper that were used during peer review. These are the versions that the peer review comments apply to.

The versions of the paper that follow are the versions that were submitted during the peer review process. They are not the versions of record or the final published versions. They should not be cited or distributed as the published version of this manuscript.

BMJ Open is an open access journal and the full, final, typeset and author-corrected version of record of the manuscript is available on our site with no access controls, subscription charges or pay-per-view fees (http://bmjopen.bmj.com).

If you have any questions on BMJ Open's open peer review process please email info.bmjopen@bmj.com

BMJ Open

Optimizing the measurement of reproductive, maternal and newborn health and nutrition knowledge among pregnant women in India: are phone surveys a reliable modality for data collection?

Journal:	*BMJ Open*
Manuscript ID	bmjopen-2021-056076
Article Type:	Original research
Date Submitted by the Author:	03-Aug-2021
Complete List of Authors:	Ng, Angela; Johns Hopkins University Bloomberg School of Public Health<br>Mohan, Diwakar; Johns Hopkins University Bloomberg School of Public Health<br>Shah, Neha; Johns Hopkins University Bloomberg School of Public Health<br>Scott, Kerry; Johns Hopkins University Bloomberg School of Public Health<br>Ummer, Osama; Oxford Policy Management; BBC Media Action,<br>Chamberlain, Sara; BBC Media Action, BBC Media Action, India; BBC Media Action, Asia<br>Bhatnagar, Aarushi; World Bank New Delhi Office, Health, Nutrition and Population<br>Dhar, Diva; Bill & Melinda Gates Foundation<br>Agarwal , Smisha ; Johns Hopkins University Bloomberg School of Public Health, Department of International Health<br>Ved, Rajani; National Health Systems Resource Centre<br>LeFevre, Amnesty; International Health
Keywords:	Community child health < PAEDIATRICS, Maternal medicine < OBSTETRICS, Neonatal intensive & critical care < INTENSIVE & CRITICAL CARE

SCHOLARONE™
Manuscripts

I, the Submitting Author has the right to grant and does grant on behalf of all authors of the Work (as defined in the below author licence), an exclusive licence and/or a non-exclusive licence for contributions from authors who are: i) UK Crown employees; ii) where BMJ has agreed a CC-BY licence shall apply, and/or iii) in accordance with the terms applicable for US Federal Government officers or employees acting as part of their official duties; on a worldwide, perpetual, irrevocable, royalty-free basis to BMJ Publishing Group Ltd ("BMJ") its licensees and where the relevant Journal is co-owned by BMJ to the co-owners of the Journal, to publish the Work in this journal and any other BMJ products and to exploit all rights, as set out in our licence.

The Submitting Author accepts and understands that any supply made under these terms is made by BMJ to the Submitting Author unless you are acting as an employee on behalf of your employer or a postgraduate student of an affiliated institution which is paying any applicable article publishing charge ("APC") for Open Access articles. Where the Submitting Author wishes to make the Work available on an Open Access basis (and intends to pay the relevant APC), the terms of reuse of such Open Access shall be governed by a Creative Commons licence – details of these licences and which Creative Commons licence will apply to this Work are set out in our licence referred to above.

Other than as permitted in any relevant BMJ Author's Self Archiving Policies, I confirm this Work has not been accepted for publication elsewhere, is not being considered for publication elsewhere and does not duplicate material already published. I confirm all authors consent to publication of this Work and authorise the granting of this licence.

For peer review only

**Title**	Optimizing the measurement of reproductive, maternal and newborn health and nutrition knowledge among pregnant women in India: are phone surveys a reliable modality for data collection?
**Author's Names**	Angela Ng, Diwakar Mohan (0000-0002-7532-366X), Neha Shah, Kerry Scott (0000-0003-3597-9637), Osama Ummer (0000-0002-4189-5328), Sara. Chamberlain (0000-0003-4785-6482), Aarushi Bhatnagar, Diva Dhar, Smisha Agarwal, Rajani Ved, Amnesty E. LeFevre (0000-0001-8437-7240), on behalf of the Kilkari Impact Evaluation Team*
**Address for each author**	**Angela Ng MSPH**<br>Department of International Health, Johns Hopkins Bloomberg School of Public Health, 615 N. Wolfe St, Baltimore, Maryland, USA<br>angelang1201@gmail.com<br><br>**Diwakar Mohan DrPH MD**<br>Title: Assistant Scientist<br>Department of International Health, Johns Hopkins Bloomberg School of Public Health, 615 N. Wolfe St, Baltimore, Maryland, USA<br>dmohan3@jhu.edu<br>ORCiD iD: 0000-0002-7532-366X<br><br>**Neha Shah MSPH**<br>Title: Research Associate<br>Department of International Health, Johns Hopkins Bloomberg School of Public Health, 615 N. Wolfe St, Baltimore, Maryland, USA<br>nshah67@jhu.edu<br><br>**Kerry Scott PhD, MSc**<br>Title: Associate<br>Department of International Health, Johns Hopkins Bloomberg School of Public Health, 615 N. Wolfe St, Baltimore, Maryland, USA<br>kscott26@jhu.edu<br>ORCiD iD: 0000-0003-3597-9637<br><br>**Osama Ummer MHA, BAMS**<br>Title: Research Manager<br>(1) BBC Media Action, India<br>Innov8 Old Fort Saket District Mall, Saket District Centre, Sector 6, Pushp Vihar, New Delhi, Delhi 110017, India<br>(2) Oxford Policy Management-Delhi, 4/6 First Floor, Siri Fort Institutional Area, New Delhi, Delhi 110049, India<br>kposamaummer@gmail.com<br>ORCiD iD: 0000-0002-4189-5328<br><br>**Sara Chamberlain, MA**<br>Title: Digital Director, BBC Media Action, India<br>Innov8 Old Fort Saket District Mall, Saket District Centre, Sector 6, Pushp Vihar, New Delhi, Delhi 110017, India<br>sara.chamberlain@in.bbcmediaaction.org<br>ORCiD iD: 0000-0003-4785-6482<br>**Aarushi Bhatnagar**<br>Title: Health Economist

For peer review only

	Oxford Policy Management-Delhi, 4/6 First Floor, Siri Fort Institutional Area, New Delhi, Delhi 110049, India<br>aarushibhatnagar@gmail.com<br><br>**Diva Dhar**<br>Title: Senior Program Officer, Gender Data & Evidence<br>Bill and Melinda Gates Foundation, 500 5th Ave N, Seattle, WA 98109 USA<br>Diva.Dhar@gatesfoundation.org<br><br>**Smisha Agarwal**<br>Title: Assistant Professor<br>Department of International Health, Johns Hopkins Bloomberg School of Public Health, 615 N. Wolfe St, Baltimore, Maryland, USA<br>sagarw23@jhu.edu<br><br>**Rajani Ved**<br>Title: Executive Director<br>National Health Systems Resource Centre, Baba Gangnath Marg, Munirka, New Delhi 110067<br>rajani.ved@gmail.com<br><br>**Amnesty LeFevre PhD, MHS**<br>Title: Associate Professor<br>1. School of Public Health and Family Medicine, University of Cape Town, Cape Town, South Africa<br>2. Department of International Health, Johns Hopkins Bloomberg School of Public Health, 615 N. Wolfe St, Baltimore, Maryland, USA<br>Email: aelefevre@gmail.com<br>ORCiD iD: 0000-0001-8437-7240<br><br>On behalf of the Kilkari Impact Evaluation Team<br>*(listed in Alphabetical order) Smisha Agarwal, Salil Arora, Jean JH Bashingwa, Aarushi Bhatanagar, Sara Chamberlain, Rakesh Chandra, Arpita Chakraborty, Neha Dumke, Priyanka Dutt, Anna Godfrey, Suresh Gopalakrishnan, Nayan Kumar, Simone Honikman, Alain Labrique, Amnesty LeFevre, Jai Mendiratta, Molly Miller, Radharani Mitra, Diwakar Mohan, Deshen Moodley, Nicola Mulder, Angela Ng, Dilip Parida, Nehru Penugonda, Sai Rahul, Shiv Rajput, Neha Shah, Kerry Scott, Aashaka Shinde, Aaditya Singh, Nicki Tiffin, Osama Ummer, Rajani Ved, Falyn Weiss, Sonia Whitehead
**Contributions**	AEL is the overall study PI and conceived the idea for this study along with DM, AB, RV, KS, and SC. Data collection tools were designed by AL, NS, DM, KS, AB, SA, OU, and RV with inputs from DD. Phone survey data collection was led and supervised by NS and RV. Face to face data collection was supervised by AB, OU, AEL, DM and AL. AN and NS led the analyses with oversight from DM and inputs from AL. AN and AL wrote the manuscripts with edits and inputs from all co-authors. AN generated all final tables and figures.
**Corresponding Author**	**Angela Ng, MSPH**<br>Department of International Health, Johns Hopkins Bloomberg School of Public Health, 615 N. Wolfe St, Baltimore, Maryland, USA<br>angelang1201@gmail.com

For peer review only

Copyright: The Corresponding Author has the right to grant on behalf of all authors and does grant on behalf of all authors, an exclusive licence on a worldwide basis to the BMJ Publishing Group Ltd to permit this article (if accepted) to be published in BMJ editions and any other BMJPGL products and sublicences such use and exploit all subsidiary rights, as set out in our licence.

Competing interests: All authors have completed the Unified Competing Interest form (available on request from the corresponding author) and declare that the research reported was funded by the Bill and Melinda Gates Foundation. AG, SC, PD are employed by BBC Media Action; one of the entities supporting program implementation. The authors do not have other relationships and are not engaged in activities that could appear to have influenced the submitted work.

Transparency declaration: I, Angela Ng, affirm that the manuscript is an honest, accurate, and transparent account of the study being reported; that no important aspects of the study have been omitted; and that any discrepancies from the study as planned (and, if relevant) have been explained.

Ethics: Institutional Review Boards from the Johns Hopkins Bloomberg School of Public Health in Baltimore, Maryland USA and Sigma Research and Consulting in Delhi, India provided ethical clearance for study activities. Verbal informed consent was obtained from all study participants.

Funding: Bill and Melinda Gates Foundation Role of study sponsors: The study sponsors had no role in the design, implementation or interpretation of study findings. The study team were independent from the funders.

Patient and public involvement: Patients were first engaged upon identification in their households as part of a household listing carried out in mid/ late 2018. Those meeting eligibility criteria were interviewed as part of the baseline survey, and ultimately randomized to the intervention and control arms. Prior to the administration of the baseline, a small number of patients were involved in the refinement of survey tools through qualitative interviews, including cognitive interviews, which were carried out to optimise survey questions, including the language and translation used. Finalised tools were administered to patients at baseline and endline, and for a sub-sample of the study population, additional interviews carried out over the phone and via qualitative interviews between the baseline and endline surveys. Unfortunately, because of COVID-19 patients and associated travel restrictions could not be involved in the dissemination of study findings.

- How was the development of the research question and outcome measures informed by patients' priorities, experience, and preferences?
    - Survey questions were pilot tested amongst beneficiaries and inputs on content wording and language translation used to enhance survey content
- How did you involve patients in the design of this study?
    - Patients were recruited during a household survey listing as described in the study protocol and informed at the time of consent of the potential for inclusion in a range of added research activities including the phone surveys. Only those who consented were included. At the time of the phone survey, a second informed consent was taken and only those respondents who consented to give their time to the interview were included.
- How will the results be disseminated to study participants?
    - COVID travel restrictions prevent added face to face contact with the communities. results have been disseminated and presented to a range of national level stakeholders, including government and donor organisations
- Were patients involved in the recruitment to and conduct of the study?
    - No

For peer review only

- For randomised controlled trials, was the burden of the intervention assessed by patients themselves?
    - Not applicable

Data Sharing Statement: Extra data can be found by emailing Amnesty LeFevre at aelefevre@gmail.com

For peer review only

Abstract (300 of 300)

Introduction: Efforts to understand the factors underpinning the uptake of reproductive, maternal and child health, and nutrition (RMNCH&N) services in high disease burden low-resource settings have often focused on face-to-face surveys or direct observations of service delivery. Increasing access to mobile phones has led to growing interest in phone surveys as a rapid, low-cost alternatives to face-to-face surveys. We assess determinants of RMNCH&N knowledge among pregnant women with access to phones and examine the reliability of alternative modalities of survey delivery.

Methods: A cross-sectional face-to-face survey was administered to women 5-7 month's pregnant with access to a phone (n=5,095) in 4 districts of Madhya Pradesh, India. Within two weeks of the original survey, two separate surveys were conducted on random samples of these women: face-to-face (Test-retest; n=205), and caller attended telephone interviews (CATI; n=375). Analyses included bivariate analyses, multivariable linear regression, and prevalence and bias-adjusted kappa analyses.

Results: Knowledge scores were low across domains: 52% for maternal nutrition and pregnancy danger signs, 58% for family planning, 47% for essential newborn care, 56% infant and young child feeding, and 58% for infant and young child care. Higher knowledge was associated with older age, higher levels of education and literacy; nuclear family accommodations; primary health decision making; greater attendance in antenatal care; and satisfaction with ASHA services. Survey questions with low interrater and intermodal reliability included infant vaccination, complementary feeding, family planning, and anemia questions. Open-ended or multi-response questions had lower intermodal reliability than when administered face-to-face. Reliability may have been hampered by the sensitivity of the content, lack of privacy, enumerators and respondents profile differences, rapport, social desirability bias, and/or enumerator's ability to adequately convey concepts or probe.

Conclusions: Phone surveys are a reliable modality for generating data about pregnant women's knowledge, however, careful revisions are needed when capturing sensitive content.

ARTICLE SUMMARY

Strengths and limitations of this study:

- Phone surveys are a rapid, low-cost alternative to face-to-face surveys and particularly useful in the current context where travel is limited due to COVID-19.
- Despite their potential, little evidence has been presented on how to optimally develop survey tools which can be reliably used to capture data.
- This study aims to address this limitation by outlining a robust process for tool design and reliability testing amongst rural women in India with limited literacy, access to phones and digital skills.
- This study was limited to women who reported having access to a mobile phone and were 5-7 months pregnant in four predominately rural districts of Madhya Pradesh.
- We note that phone surveys as modality for capturing health information data have limited generalisability given that nearly half of women in India do not have phone access.

For peer review only

INTRODUCTION

Despite declines in maternal and child mortality from 1990 to 2015 of 77% and 60% respectively, India fell short of attaining the millennium develop goals for maternal and child health (1). High rates of maternal and child mortality are underpinned by a range of interacting factors including the social determinants of health; deficits in knowledge, the availability and quality of health care; and suboptimal health careseeking by families. Efforts to measure health practice, prevention, and careseeking in India have most commonly been done via face-to-face survey methods, including the population-based National Family Health Surveys (NFHS) carried out approximately every 5 to 7 years (2,3). In addition to being costly and time intensive, these population based surveys are limited by lengthy recall bias (4). In 2015-16, NFHS survey findings suggested that household access to mobile phones in India is near universal (94% urban and 80% rural) and nearly half (48%) of women have a mobile phone that they themselves use (5). Growing access to mobile phones at a household level, and more specifically among women, provides an opportunity to develop alternative modalities for the routine collection of data on health behaviors and knowledge. Despite their immense potential, few applications of phone surveys have been reported for measuring health knowledge, behaviors and outcomes in India (3) and even less information is available on the methods underpinning the development of these tools.

As phone ownership and access increase in India (6), a reliable phone survey tool would enable timely, routine and low cost measurement of the population health status, including knowledge, careseeking, and practices (7). There is a growing importance and reliance on remote data collection including phone surveys (in India and elsewhere) as COVID has impacted the ease and budgets for data collection (8). In this manuscript, we outline the process for developing and refining a phone survey tool for the measurement of reproductive, maternal and newborn health and nutrition (RMNCH&N) knowledge among pregnant women in four districts (Rewa, Mandsaur, Hoshangabad, and Rajgarh) of Madhya Pradesh. We start by developing overall and domain-specific RMNCH&N knowledge scores and then identify the determinants of higher knowledge scores (Figure 1). We then assess differences in the reliability of the knowledge questions over different modalities including face-to-face surveys at two time points (hereafter called test-retest) and caller attended telephone interviews (CATI; hereafter called phone surveys). Study findings illuminate gaps in RMNCH&N knowledge among pregnant women, determinants of these knowledge levels, and the reliability of phone survey tools compared to face-to-face tools.

METHODS

Study Setting

Data collection activities were carried out in four districts (Hoshangabad, Mandsaur, Rewa, and Rajgarh) of Madhya Pradesh; a state in central India with a population of over 75 million (9). The population of Madhya Pradesh is predominately Hindu and based in rural areas (9). Significant gaps between men and women persist with regard to literacy (82% of men are literate as compared to 59% of women), and only 29% of women report having access to mobile phones that they themselves can use (10). For nearly all health indicators, Madhya Pradesh falls below national averages. Despite near universal attendance of at least one antenatal care visit during pregnancy, only 53% of women receive services within the first trimester, and 36% receive the recommended four visits (10). While institutional delivery rates have increased over time, 20% of women deliver outside the formal health sector, and only 18% receive a health check after birth from a trained provider within 2 days of birth (10). Among children, 51 per 1,000 live births will die prior to their first birthday and 65 per 1,000 live births will die prior to their fifth birthday (10). High mortality rates are underpinned by undernutrition (43% of children are under weight, 42% are stunted), low rates of exclusive breastfeeding (58%), and gaps in access to basic health services including immunization. Where the latter is concerned, only 73% of children 12-23 months have received the recommended 3 doses of the DPT vaccine (10).

Study design and sampling

Survey data were captured as part of baseline survey activities conducted as part of the impact evaluation of a mobile health intervention (Kilkari) described in-depth elsewhere (11). A cross-sectional survey was conducted among (n=5,095) women with reported access to a mobile phone during the day (Survey Activity A in Figure 2). The survey sample size was powered to detect a 5% change in the practice of exclusive breastfeeding from baseline to endline (12 months later), assuming an alpha of 0.05, power of 0.80, 20% loss to follow-up, and 35% loss of women due to changes in mobile phone numbers (SIM change).

To assess reproducibility in the surveys, stability of the instrument, and inter-rater reliability, a subsample of women who completed the main survey was re-interviewed within two weeks to assess the reliability of the responses from the main survey (test-retest; Survey Activity B in Figure 2). Assuming a kappa of 0.80, margin of error 0.05, and alpha of 0.5, proportion of positive responses of 0.35 for rater 1 and 0.40 for rater 2, 146 participants were needed. To accommodate a 15% loss to follow up or refusal, a minimum of 168 women were targeted and ultimately 205 interviewed.

In order to develop a validated phone survey tool and observe inter-modal reliability, pregnant women who previously completed a face-to-face interview and were not interviewed in the test-retest phase were interviewed a second time over the phone (Survey Activity C in Figure 2) two weeks after completing the baseline face-to-face survey. Assuming a kappa of 0.80, margin of error 0.05, and alpha of 0.5, proportion of positive responses of 0.35 for rater 1 and 0.40 for rater 2, a minimum sample size of 146 participants were needed. Assuming a 20% response rate, a random sample of 880 women were contacted within 1-2 weeks of the initial face-to-face interview, and ultimately 375 completed the interviews.

Data collection

The survey included seven modules on: 1. mobile phone ownership, use, and digital literacy; 2. general pregnancy, delivery, child health and family planning knowledge; 3. birth history; 4. experiences during pregnancy care; 5. content of pregnancy care; 6. delivery and postpartum care intentions; and 7. client satisfaction with ASHA services. This manuscript focuses on knowledge; findings from other modules are reported elsewhere (12, 13). Female enumerators conducted face to face and phone surveys. The phone surveys were conducted out of the National Health Systems Resource Center in New Delhi.

Data analysis

The data collected were analyzed using Stata 15 (Stata Corporation, College Station, TX, USA). First, a conceptual framework was developed comprised of factors believed to underpin pregnant women's knowledge from previous literature (10-23). At the individual level, characteristics such as phone ownership, health care characteristics, socioeconomic, and sociodemographic factors were considered. A wealth index was created by dividing the women into 5 quintiles using principal component analysis. At the community level, social gender norms were analyzed such as participation in self-help groups, women being the primary health decision maker, and family living structure as well as geographic characteristics of the community.

In order to best categorize knowledge among pregnant women, survey questions were consolidated into five thematic domains: 1. Maternal nutrition and pregnancy danger signs, 2. Family planning, 3. Essential newborn care, 4. Infant and young child feeding, and 5. Infant and young child care (Supplementary Table 1). For each question, if there was only one correct answer, a binary score was given: 1 if the women knew the correct answer, 0 if not. If there were multiple correct answer options, each correct option was weighted so if all correct options were selected, the final score for that question would be 1. Each question then had equal weight in its category. All the knowledge domains were averaged together at the end to create a final composite score.

A simple linear regression was performed on the final composite score with the factors thought to have an impact on them. A multiple linear regression was performed to see all together which factors would have

For peer review only

an impact on overall women's knowledge. In order to find the best model, only variables with a p-value of 0.20 or less during bivariate analyses were used. A multiple linear regression was also performed on each individual knowledge domain to see if particular factors influenced one category more than another.

If the difference in prevalence between survey questions was <15%, kappa scores were used to assess the validity of the question. Reliability analyses aimed to determine the stability and consistency of results. Cronbach's alpha of 0.7 or higher is the cutoff for determining a sufficient evidence of reliability. Kappa coefficients were adjusted for the difference in prevalence levels using the Prevalence Adjusted Bias Adjusted Kappa (PABAK) score. This was to determine if the questions themselves were reliable, and if so, if the question was reliable over the phone. If the threshold of 0.7 was met for just the test-retest, the question was deemed to be reliable for face-to-face surveys in this context. If the threshold of 0.7 was met for both test-retest and phone survey, the question was deemed reliable for phone surveys in this context.

Ethics Approval

Ethics approval for this research study was obtained through Johns Hopkins School of Public Health Institutional Review Board in Baltimore, Maryland, as well as Sigma Research and Consulting in New Delhi, India. All participants consented to the study prior to interview.

RESULTS

Pregnant Women Characteristics

Among 5,095 interviewed through the face-to-face, 95% were Hindu, 58% were between 18-24 years of age, 68% had at least one child, 47% belonged to other backward castes, 8% belong to a self-help group, 41% had completed high school or higher, and 57% were able to read the whole sentence (Table 1). A majority of the women interviewed resided in Rewa (57%), followed by Mandsaur, Hoshangabad, and Rajgarh falling at 16%, 8%, and 19% respectively.

Table 1. Characteristics of pregnant women drawn from a face-to-face survey in 4 districts of Madhya Pradesh.

Characteristic	n	Frequency
**Age**		
18-24	2,975	58.4%
25-34	2,023	39.7%
35+	97	1.90%
**Religion**		
Hindu	4,848	95.2%
Muslim	241	4.73%
Other	6	0.12%
**Caste**		
General	1,133	22.2%
Other Backward Caste	2,386	46.8%
Scheduled Caste/Tribe	1,576	30.9%
**Parity (>=1 Child)**	3,486	68.4%
**Employed**	1,921	37.7%
**Education**		
No School	548	10.8%
Primary or less	876	17.2%
Middle School	1,560	30.6%
High School	1,666	32.7%
Higher Education	445	8.70%
**Literacy**		

For peer review only

Cannot read at all	1,659	32.6%
Able to read only parts of sentence	554	10.9%
Able to read whole sentence	2,880	56.6%
**Nuclear family structure**	1,092	21.6%
**Self Help Group**	380	7.46%
**Woman is the primary decision maker for health decisions**	1,451	28.5%
**Attendance of maternity care services**		
Antenatal care 1	742	14.6%
Antenatal care 2	1,225	24.0%
Antenatal care 3	952	18.7%
Antenatal care 4+	2,176	42.7%
**Satisfied with the services provided by the ASHA**	4,699	92.2%
**Owns Phone**	3,860	75.8%
**District**		
Hoshangabad	406	7.97%
Mandsaur	821	16.1%
Rewa	2,920	57.3%
Rajgarh	948	18.6%

Determinants of RMNCH&N knowledge

Figure 3 shows average knowledge scores for the five composite knowledge domains: maternal nutrition and pregnancy danger signs (52%), family planning score (58%), immediate post-birth care for the neonate (47%), infant and young child feeding (56%), and infant and young child care (58%). The average of all domains was used to create a final composite score (54%).

Table 2 presents results from bivariate and multivariate regressions on factors underpinning pregnant women's knowledge. In the multivariable model, women's knowledge was significantly higher among older women (25-34 years, p<0.001; >34 years, p=0.02); with higher education (p=0.009); able to read a full sentence (p<0.001); living in a nuclear family (p<0.001); who reported being the primary decision maker in health decisions (p<0.001); had attended a greater number of ANC visits (p<0.001); and reported being satisfied with ASHA services (p<0.001). Women from the rural districts of Mandsaur and Rajgarh had significantly lower knowledge scores than women in the reference peri-urban district of Hoshangaband. Women's phone ownership, socioeconomic status, and caste were not associated with significant differences in knowledge.

Table 2. Factors associated with pregnant women's RMNCH&N knowledge in four districts of Madhya Pradesh, India

Variable	Unadjusted Coefficient (95% Confidence Interval)	p-value	Adjusted Coefficient (95% Confidence Interval)	p-value
**Age**				
18-24	1	-	1	--
25-34	3.21(2.68-3.74)	<0.001	1.91(1.35-2.46)	<0.001
35+	1.87(-0.04-3.78)	0.055	2.04(0.34-3.74)	0.02
**Parity (>=1 child)**	4.40(3.85-4.96)	<0.001	4.39(3.74-5.03)	<0.001
**Caste**				
General	1	-	1	-

For peer review only

Other backwards	-0.62(-1.30-0.05)	0.070	0.62(-0.06-1.3)	0.076
Scheduled	-0.97(-1.70-0.24)	0.010	0.24(-0.58-1.06)	0.567
**Wealth Index**				
Q1	1	--	1	--
Q2	0.30(-0.52-1.13)	0.471	0.32(-0.49-1.13)	0.442
Q3	0.03(-0.80-0.86)	0.938	0.14(-0.78-1.07)	0.765
Q4	0.25(-0.58-1.08)	0.551	0.16(-0.83-1.16)	0.751
Q5	1.83(1.00-2.66)	<0.001	0.72(-0.38-1.82)	0.197
**Education level**				
No School	1	--	1	--
Primary or Less	0.71(-0.31-1.72)	0.172	0.65(-0.35-1.65)	0.201
Middle School	1.32(0.39-2.24)	0.005	0.6(-0.51-1.72)	0.289
Higher Education	3.26(2.37-4.16)	<0.001	1.65(0.42-2.89)	0.009
**Literacy**				
Cannot read at all	1	--	1	-
Reading only parts of sentence	1.43(0.52-2.34)	0.002	0.95(0.01-1.9)	0.049
Read whole sentence	3.02(2.44-3.59)	<0.001	2.29(1.42-3.15)	<0.001
**Self Help Group**	0.97(-0.03-1.97)	0.057	0.4(-0.42-1.22)	0.335
**Nuclear Family Structure**	-1.97(-2.61--1.33)_	<0.001	-2.14(-2.95--1.33)	<0.001
**Women is the primary decision maker in health decisions**	2.03(1.45-2.61)	<0.001	1.59(1-2.19)	<0.001
**Number of ANC Visits**				
1	1	--	1	--
2	1.13(0.27-2.01)	0.011	1.03(0.16-1.9)	0.021
3	1.64(0.73-2.56)	<0.001	1.95(1.04-2.85)	<0.001
4	1.14(0.34-1.94)	0.005	1.68(0.81-2.54)	<0.001
**Satisfied with the services provided by the ASHA**	2.61(2.02-3.19)	<0.001	2.18(1.57-2.79)	<0.001
**Phone Ownership**	1.31(0.71-1.93)	<0.001	0.61(-0.01-1.22)	0.055
**District**				
Hoshangabad	1	--	1	--
Mandsaur	-3.30(-4.42--2.17)	<0.001	-3.3(-4.51--2.09)	<0.001
Rewa	-1.53(-2.52--0.55)	<0.001	-0.13(-1.21-0.95)	0.814
Rajgarh	-4.65(-5.75--3.55)	<0.001	-3.05(-4.21--1.89)	<0.001

Supplementary Table 2 explores determinants of knowledge for each of the five domains. Across all domains, knowledge was significantly higher among older women (25-34 years of age), with a least one child, able to read a whole sentence, and who reported being satisfied with services provided by the ASHA. Knowledge of maternal nutrition and pregnancy danger signs was significantly higher amongst women living in a nuclear family, with higher education, and who had attended a greater number of antenatal care visits. Family planning knowledge was significantly higher amongst women in the highest socioeconomic strata, with primary school or higher education. Knowledge of immediate post-birth care for the newborns was similarly higher amongst women in the highest socioeconomic strata, and with higher education.

For peer review only

Survey reliability

For each survey question, inter-rater reliability was assessed by comparing reported results from face-to-face surveys conducted at two time points (test-retest), while intermodal reliability was assessed by comparing findings from the face-to-face survey to phone surveys (Supplementary Table 3). The question responses were ordered by highest reliability first in the test-retest, and then those that were above the Cronbach's alpha of 0.7 were reordered in the phone survey.

Questions with high reliability included those asking women about the items that should not be eaten or drank during pregnancy (k = 0.95), to identify the benefits of breastfeeding (k = 1.00), specify types of food given to baby (k = 0.99), newborn danger signs (k = 0.99), and items given to children who have diarrhea (k = 0.98). Questions with the lowest reliability included asking women what they should do if they are anemic (k = 0.30); to state the benefits of family planning (k = 0.06); and stipulate the stereotypes of male sterilization (k = 0.28).

Questions that were proven reliable for face-to-face surveys but not phone surveys included number of tetanus shots (k = 0.77), and several questions who's response options accommodated multiple answers: expenditure of breastfeeding (k = 0.82); eggs as a food to eat during pregnancy (k=0.79), beans, peas, and nuts as a type of food a baby can eat after 6 months (k=0.75), and ORS and zinc as a solution for childhood diarrhea (k = 0.70).

Questions were also considered to have low reliability if the reported prevalence changed by more than 15% between the face-to-face survey and secondary testing, whether via retest or phone survey (Supplementary Table 4). Particularly high percent differences between face-to-face prevalence and phone prevalence were seen in asking what items should be used to cut an umbilical cord, methods for safe birth control, and female sterilization.

Figure 4 graphs the correlation between the prevalence and bias adjusted kappa statistics for face-to-face surveys against the in-person re-test and the phone survey. The correlation forms a positive linear line, representing a strong positive reliability between the two survey options.

DISCUSSION

The primary purpose of this study was to develop and refine a phone survey tool for the measurement of RMNCH&N knowledge among pregnant women in four districts of Madhya Pradesh. Our development process entailed first assessing inter-rater reliability via face-to-face surveys repeated at two time points, followed by intermodal reliability testing by comparing an initial face-to-face survey repeated in abbreviated fashion over the phone. This iterative process of testing was intended to help refine the content of the survey tool and ascertain what kinds of questions are reliable over which modality. During analyses we clubbed questions across common content areas into one of five domains: maternal nutrition and pregnancy danger signs, family planning, essential newborn care, infant and young child feeding, and infant and young child care. Mean knowledge scores across domains were generated, along with an overall mean composite score across all domains. PABAK kappa statistics were used to assess the reliability of each survey question by comparing responses to face-to-face surveys at two time points and face-to-face and phone surveys.

Three key findings emerged from this process. First, overall knowledge levels among pregnant women in these four districts of Madhya Pradesh is low ranging from 47% for essential newborn care to 58% for family planning. Areas of low knowledge included what should be put on the cord after delivery, how to keep the baby warm after delivery, what foods to eat during pregnancy, what food and drinks to avoid during pregnancy, etc. Second, and perhaps not surprisingly, knowledge scores were significantly higher amongst older women; with higher levels of education and literacy; living in a nuclear family; who reported being the primary decision maker in health decisions; who had attended a greater number of ANC visits;

For peer review only

and reported being satisfied with ASHA services. Third, knowledge can be reliably measured over the phone, but the survey tool requires modification with regard to length, content, and response options used.

Our process of modifying the tool to develop an abridged version started by first exploring inter-rater (test retest) and then inter-modal (face-to-face versus phone) reliability. Questions with high overall reliability ($\alpha >= 0.70$) for both retest and phone surveys included items that should not be consumed during pregnancy, benefits of breastfeeding, newborn danger signs, types of food that should be given to babies over six months in age, and items given to children who have diarrhea. Questions that were unreliable included family planning questions (length of time between pregnancies, benefits of using family planning, true/false questions about male sterilization) and questions about infant vaccination, complementary feeding, and anemia. Across delivery modalities a number of questions were reliable when asked face-to-face but not when asked over the phone: number of tetanus shots a woman should receive during pregnancy, and several questions with multi-answer response options. Only one question on the length of time before bathing the baby after delivery was reliable when asked face-to-face and then over the phone, but not reliable when asked through repeated face-to-face measurement (test retest).

Reasons for low reliability could include recall bias (especially for open-ended questions with multiple coded response options), social desirability, perceived redundancies in answer options, and enumerator-dependent ambiguous answers (21,22). Questions that were not reliable when asked in-person but over the phone could be attributed to concerns about privacy (i.e. someone else being the room with the pregnant women at the time of the interview); content, including questions about sensitive or taboo topics (e.g. family planning); and differences in enumerator and respondent characteristics, including language (i.e. while enumerators shared a common language with respondents, they were not from Madhya Pradesh and may have spoken with a different accent or dialect). Some questions with open-ended response options or that required the enumerator to list out answers as opposed to saying “yes” or “no” may have elicited a degree of discomfort on the part of the respondent. Some questions were also better asked face-to-face when the enumerators were allowed to elaborate or probe more and build a rapport. The questions and how they were asked could have been confusing over the phone depending on wording, translation, and concept difficulty.

Study findings contribute to the limited body of literature on how to optimise the development of phone surveys for use in the measurement of population level knowledge and health outcomes. Few studies to date have sought to refine phone survey tools through a rigourous process of reliability testing (24). Findings from a 2017 systematic review identified only 10 studies which have reported using reliability testing to develop phone survey tools; five of these included face-to-face and CATI survey comparisons (24). In Honduras and Peru (25), comparisons were made across independent samples, while in Lebanon (26) and two separate surveys in Brazil (27), comparisons were made using the same sample across two modalities (as in our study) (11, 13). Overall findings broadly suggest that there was concordance in the results across CATI and face-to-face modalities (12). However, for some outcomes, prevalence estimates did differ across modalities (28). Studies in Honduras and Peru compared additional phone survey modalities, including text messages and interactive voice response (IVR), and concluded that CATI surveys had the lowest discordance with the face-to-face surveys (29). Elsewhere, more recent efforts have sought to compare the reliability of alternative phone survey delivery modalities (IVR versus CATI) for measuring non-communicable disease risk factors (30). While findings were similar across modality, there was lower consistency in responses to questions related to history of checking for hypertension and diabetes (K ranged from 0.43 to 0.67), physical activity (K ranged from 0.10 to 0.41), weekly fruit and vegetable consumption (K ranging from 0.08 to 0.45), and salt intake ($K<0.30$) (30). Collectively, this body of literature underscores the need to carefully explore the reliability of question content, phrasing and response options across varied modalities before using phone surveys.

Limitations

For peer review only

This survey was limited to RMNCH&N knowledge in women 5-7 months pregnant with access to a mobile phone during the day, in four predominately rural districts of Madhya Pradesh. This may have led to a pro-rich bias in the sampling given the profile of beneficiaries with access to mobile phones (31). Survey questions were derived expert review and from large national survey tools, including the demographic and health survey for India and the Multiple Indicator Cluster Survey (MICS). Cognitive interviews were used to refine questions on respectful maternity care; a process described elsewhere (13, 32, 33). Ideally the same process would have been followed for refining knowledge questions. However, budget and time constraints made this added step infeasible. Phone surveys were administered by Delhi based enumerators whose demographic profile differed from respondents. Alternative phone survey modalities, including text messages, interactive voice response, and USSD were considered but deemed less desirable to using computer assisted telephone interviews (CATI) surveys which could allow beneficiaries to speak with and ask for clarification questions.

CONCLUSIONS

Study findings have led to the development of a reliable phone survey tool appropriate for measuring knowledge among pregnant women with access to a phone in rural India. Questions found to have a low reliability over the phone focused on sensitive topics such as family planning or included open ended response options. Ensuring alignment between beneficiary and respondent profile, and using a CATI modality of delivery (as opposed to IVR or text messages) is recommended for improving the reliability of survey responses. As phones become more ubiquitous in India and elsewhere globally, they offer much for potential as low cost, rapid, alternatives to face-to-face surveys for capturing health information.

ACKNOWLEDGEMENTS

We thank the women and families of Madhya Pradesh who generously gave of their time to support this work. We are humbled by the opportunity to convey their perspectives and experiences. We additionally are grateful to Neha Dumke and the team at the National Health Systems Resource Centre for their support. This work was made possible by the Bill and Melinda Gates Foundation. We thank Diva Dhar, Suhel Bidani, Rahul Mullick, Dr. Suneeta Krishnan, Dr. Neeta Goel and Dr. Priya Nanda for believing in us and giving us this opportunity. We additionally wish to thank are grateful to the larger team of enumerators from OPM-India and NHSRC who worked tirelessly over many months to implement the surveys that form the backbone of our analyses. We additionally thank Vinit Pattnaik at OPM and Erica Crawford at Johns Hopkins University for their support to the financial management support of our work.

REFERENCES

1. Global Health Observatory. India Statistics Summary. World Heal Organ [Internet]. 2019 [cited 2019 Mar 3]; Available from: http://apps.who.int/gho/data/node.country.country-IND?lang=en
2. Nair H, Williams LJ, Marsh A, et al. Assessing the reactivity to mobile phones and repeated surveys on reported care-seeking for common childhood illnesses in rural India. Journal of Global Health. 2018;8(2).
3. International Institute for Population Sciences. NATIONAL FAMILY HEALTH SURVEY (NFHS-4) 2015-16 INDIA [Internet]. Deonar, Mumbai; 2017 [cited 2019 Mar 3]. Available from: http://www.rchiips.org/nfhs
4. Manesh AO, Sheldon TA, Pickett KE, Carr-Hill R. Accuracy of child morbidity data in demographic and health surveys. International Journal of Epidemiology. 2007;37(1):194–200.
5. Mohan D, Bashingwa JJ, Tiffin N, et al. Does having a mobile phone matter? Linking phone access among women to health in India: An exploratory analysis of the National Family Health Survey. PLOS ONE. 2020;15(7).
6. International Telecommunication Union. The World in 2016: ICT Facts and Figures [Internet]. [cited 2019 Mar 9]. Available from: https://www.itu.int/en/ITU-D/Statistics/Pages/stat/default.aspx

For peer review only

7. Okonjo-Iweala N, Osafo-Kwaako P. Improving health statistics in Africa. The Lancet. 2007;370(9598):1527–8.
8. McDougal L, Raj A, Yore J, et al. Strengthening Gender Measures and Data in the COVID-19 Era : An Urgent Need for Change (English). 2021. World Bank Group. http://documents.worldbank.org/curated/en/681671619424596227/Strengthening-Gender-Measures-and-Data-in-the-COVID-19-Era-An-Urgent-Need-for-Change
9. Raju S, Ayyar Panchapagesan N. Madhya Pradesh [Internet]. Encyclopædia Britannica, inc. [cited 2019 Mar 4]. Available from: https://www.britannica.com/place/Madhya-Pradesh
10. National Family Health Survey. State Fact Sheet Madhya Pradesh [Internet]. 2016 [cited 2019 Mar 3]. Available from: http://rchiips.org/nfhs/pdf/NFHS4/MP_FactSheet.pdf
11. LeFevre A, Agarwal S, Chamberlain S, Scott K, Godfrey A, Chandra R, et al. Are stage-based health information messages effective and good value for money in improving maternal newborn and child health outcomes in India? Protocol for an individually randomized controlled trial. Trials. 2019;20(1).
12. Shah N, Mohan D, Agarwal S, et al. Novel approaches to measuring knowledge among frontline health workers in India: Are phone surveys a reliable option? PLOS ONE. 2020;15(6).
13. LeFevre AE, Scott K, Mohan D, Shah N, Bhatnagar A, Labrique A, et al. Development of a Phone Survey Tool to Measure Respectful Maternity Care During Pregnancy and Childbirth in India: Study Protocol. JMIR Research Protocols. 2019;8(4).
14. Terwee CB, Bot SDM, de Boer MR, van der Windt DAWM, Knol DL, Dekker J, et al. Quality criteria were proposed for measurement properties of health status questionnaires. Journal of Clinical Epidemiology. 2007;60(1):34–42.
15. Nigatu SG, Worku AG, Dadi AF. Level of mother ' s knowledge about neonatal danger signs and associated factors in North West of Ethiopia : a community based study. BMC Res Notes. 2015;4–9.
16. Sandberg J, Odberg Pettersson K, Asp G, Kabakyenga J, Agardh A. Inadequate Knowledge of Neonatal Danger Signs among Recently Delivered Women in Southwestern Rural Uganda: A Community Survey. PLoS ONE. 2014;9(5).
17. Senarath U, Fernando DN, Vimpani G, Rodrigo I. Factors associated with maternal knowledge of newborn care among hospital-delivered mothers in Sri Lanka. Transactions of the Royal Society of Tropical Medicine and Hygiene. 2007;101(8):823–30.
18. Singh A, Singh KK, Verma P. Knowledge, attitude and practice GAP in family planning usage: an analysis of selected cities of Uttar Pradesh. Contraception and Reproductive Medicine. 2016;1(1).
19. Sharma V, Mohan U, Das V, Awasthi S. Socio demographic determinants and knowledge, attitude, practice: Survey of family planning. Journal of Family Medicine and Primary Care. 2012;1(1):43.
20. Acharya AS, Kaur R, Prasuna JG, et al. Making Pregnancy Safer-Birth Preparedness and Complication Readiness Study Among Antenatal Women Attendees of A Primary Health Center, Delhi. Indian Journal of Community Medicine. 2015;40(2):127.
21. Deoki N, Kushwah SS, Dubey DK, et al. A study for assessing birth preparedness and complication readiness intervention in Rewa District of Madhya Pradesh Chief Investigator, India. Department of Community Medicine, SS Medical College, Rewa, MP. 2008;9.
22. Cannell CF, Miller PV, Oksenberg L. Research on Interviewing Techniques. Sociological Methodology. 1981;12:389.
23. Nair H, Williams LJ, Marsh A, et al. Assessing the reactivity to mobile phones and repeated surveys on reported care-seeking for common childhood illnesses in rural India. Journal of Global Health. 2018;8(2).
24. Greenleaf AR, Gibson DG, Khattar C, et al. Building the Evidence Base for Remote Data Collection in Low- and Middle-Income Countries: Comparing Reliability and Accuracy Across Survey Modalities. Journal of Medical Internet Research. 2017;19(5).
25. Ballivian A, Azevedo J, Durbin W, et al. Using mobile phones for high-frequency data collection. In: Toninelli D, Pinter R, de Pedraza P, editors. Mobile Research Methods: Opportunities and

For peer review only

Challenges of Mobile Research Methodologies. London: Ubiquity Press; 2015:21-39.
26. Mahfoud Z, Ghandour L, Ghandour B, et al. Cell phone and face-to-face interview responses in population-based surveys: how do they compare? Field Methods 2014 Jul 01;27(1):39-54.
27. Ferreira A, César CC, Malta DC, et al. Validade de estimativas obtidas por inquérito telefônico: comparação entre VIGITEL 2008 e inquérito Saúde em Beagá. Rev bras epidemiol 2011 Sep;14(1):16-30.
28. Francisco P, Azevedo Barros MB, Segri N, et al. Comparação de estimativas para o auto-relato de condições crônicas entre inquérito domiciliar e telefônico - Campinas (SP), Brasil. Rev bras epidemiol 2011 Sep;14(3):5-15.
29. Ballivian A, Azevedo J, Durbin W, et al. Using mobile phones for high-frequency data collection. In: Toninelli D, Pinter R, de Pedraza P, editors. Mobile Research Methods: Opportunities and Challenges of Mobile Research Methodologies. London: Ubiquity Press; 2015:21-39.
30. Pariyo GW, Greenleaf AR, Gibson DG, Ali J, Selig H, Labrique AB, et al. Does mobile phone survey method matter? Reliability of computer-assisted telephone interviews and interactive voice response non-communicable diseases risk factor surveys in low and middle income countries. PLOS ONE. 2019;14(4).
31. Mohan D, Bashingwa JJ, Tiffin N, Dhar D, Mulder N, George A, et al. Does having a mobile phone matter? Linking phone access among women to health in India: An exploratory analysis of the National Family Health Survey. PLOS ONE. 2020;15(7).
32. Scott K, Gharai D, Sharma M, Choudhury N, Mishra B, Chamberlain S, et al. Yes, no, maybe so: the importance of cognitive interviewing to enhance structured surveys on respectful maternity care in northern India. Health Policy and Planning. 2019
33. Scott K, Ummer O, LeFevre AE. The devil is in the detail: reflections on the value and application of cognitive interviewing to strengthen quantitative surveys in global health. Health Policy and Planning. 2021;36(6):982–95.

For peer review only

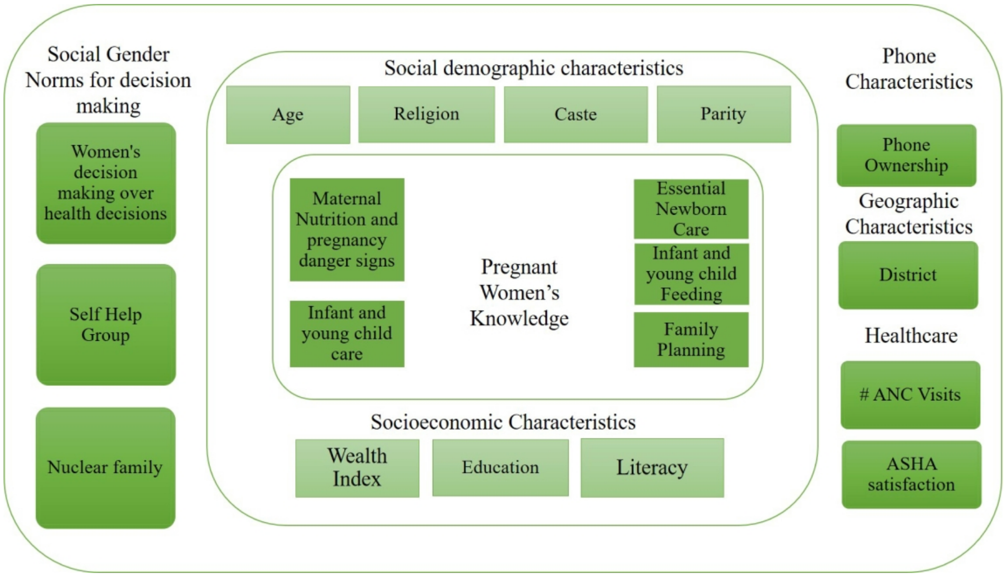

Figure 2. Conceptual framework for identifying the determinants of pregnant women's RMNCH&N knowledge

670x381mm (300 x 300 DPI)

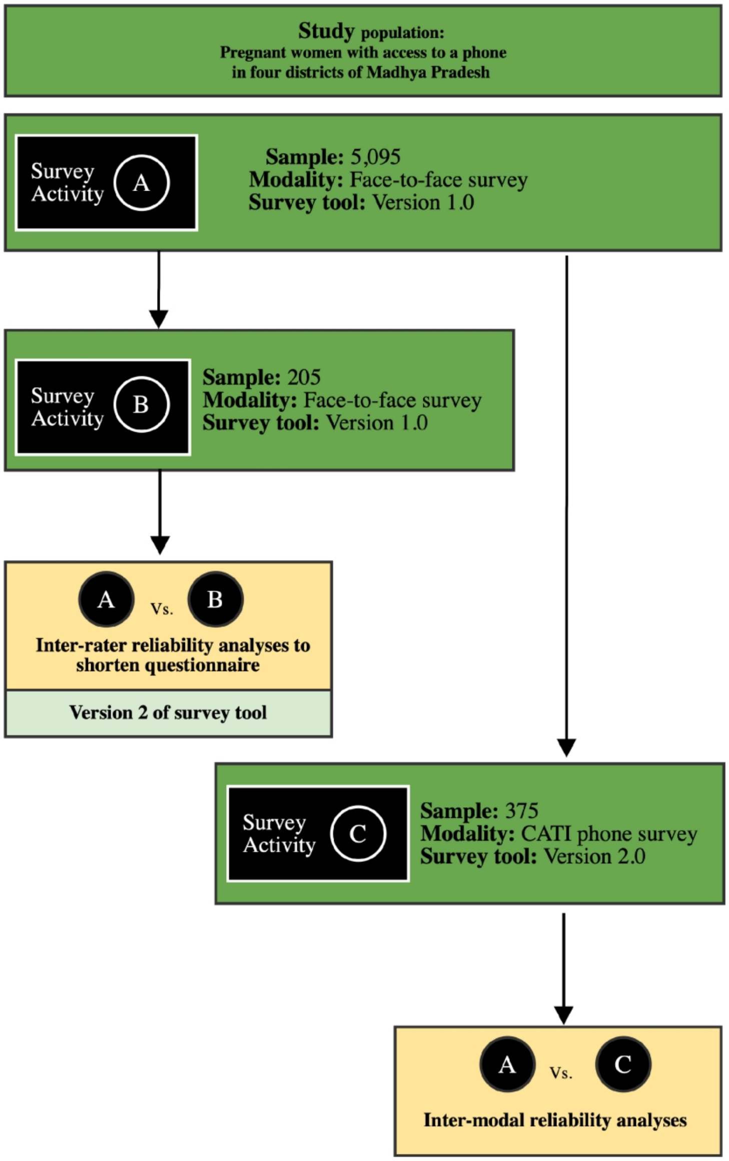

Figure 1. Completed interviews for face-to-face and CATI surveys

177x273mm (300 x 300 DPI)

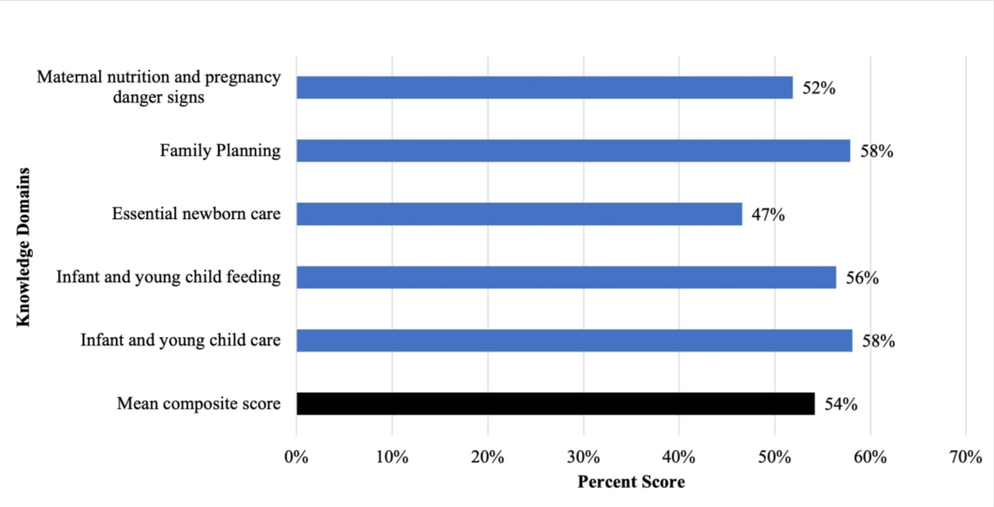

Figure 4. Average knowledge scores for composite knowledge domains

212x109mm (300 x 300 DPI)

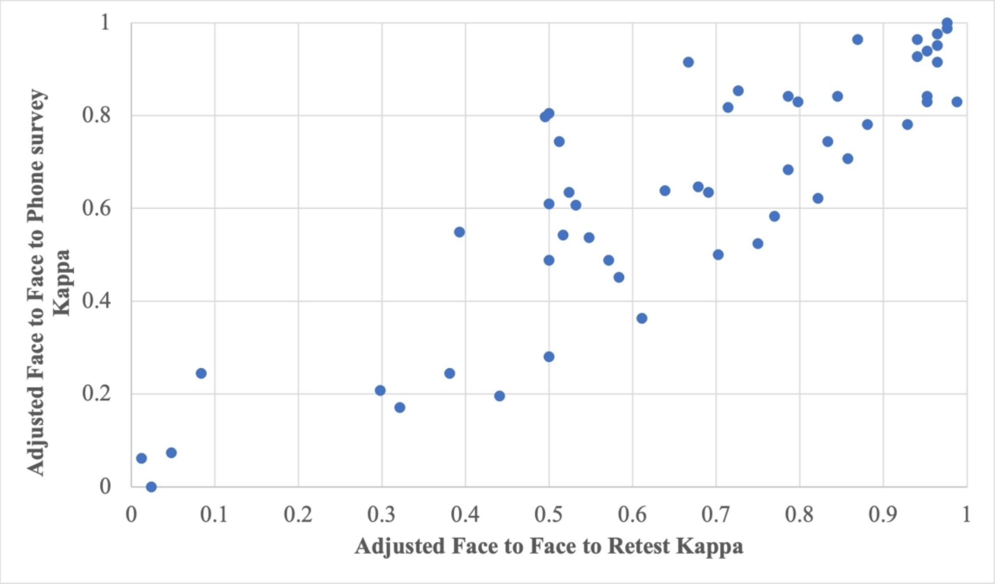

Figure 3. Measurement of degree in which repeated measurements in pregnant women interviewed (test-retest) provide similar answers

180x105mm (300 x 300 DPI)

Supplementary Table 1: Questions included in each domain of knowledge

Questions	Answer options	Correct answer(s)
**Maternal nutrition and pregnancy danger signs (6 questions)**		Maximum possible score: 6
What foods should you eat during pregnancy?	Unprompted free list, enumerator noted any correct answers mentioned	Milk/dairy products; fruits; green leafy vegetables; pulses and nuts; egg; meat; fish
What should you not eat or drink during your pregnancy?	Unprompted free list, enumerator noted any correct answers mentioned	Tea; coffee; cigarettes/bidis; alcohol
What should you do if you are anemic?	Unprompted free list, enumerator noted any correct answers mentioned	Have your blood checked regularly; take IFA tablets daily; eat well
What are the benefits of taking Iron Folic Acid tablets during pregnancy?	Unprompted free list, enumerator noted any correct answers mentioned	Help prevent/treat anemia; improve the health/well-being of my baby
How many tetanus injections should you have during pregnancy?	Number or don't know	2
What danger signs during pregnancy and before labor starts would lead you to go to the health facility immediately?	Unprompted free list, enumerator noted any correct answers mentioned	Vaginal bleeding; vaginal discharge; convulsions; stomach cramps; swelling on limbs and face; yellowing of skin; fever; decreased or absent fetal movements
**Immediate post-birth care for the neonate (4 questions)**		Maximum possible score: 4
How soon after delivery should your baby be bathed?		
How should you keep the baby warm after delivery?	Unprompted free list, enumerator noted any correct answers mentioned	
What should be used to cut the umbilical cord?	Unprompted free list, enumerator noted any correct answers mentioned	
What should be put on the cord after delivery?	Unprompted free list, enumerator noted any correct answers mentioned	
**Infant and young child feeding (5 questions)**		Maximum possible score: 5
What are some of the benefits of breastfeeding?	Unprompted free list, enumerator noted any correct answers mentioned	Promotes mother-baby bonding; helps build immunity for child; promotes child growth, wellness; Reduces expenditure on medical care as child will fall sick less; the more the child breastfeeds, the more milk

For peer review only

Questions	Answer options	Correct answer(s)
		will be produced; it helps to maintain space between two children
How soon after delivery should you breastfeed your child?	Enumerator noted “immediately”, number of hours or days mentioned, or “don’t know”	Immediately or <1 hour
How many times per day should newborn babies be breastfed?	Enumerator can note if respondent provides either of the correct answers or “don’t know”	9-10 times in 24 hours; On-demand (as often as the baby wants to feed)
How soon after delivery should you give foods or liquids other than mother's milk?		Six months
What types of foods should a baby be given after 6 months of age?	Unprompted free list, enumerator noted any correct answers mentioned	
**Infant and young child care (5 questions)**		Maximum possible score: 5
What danger signs do you know about for the newborn after delivery that would cause you to take the baby to the health facility immediately?	Unprompted free list, enumerator noted any correct answers mentioned	
How soon after your baby is born should it receive its first vaccination?		
What should you give your child to treat diarrhea?	Unprompted free list, enumerator noted any correct answers mentioned	
What are things you can do to prevent your child from getting diarrhea?	Unprompted free list, enumerator noted any correct answers mentioned	
What are three critical times for a woman to wash her hands?	Unprompted free list, enumerator noted any correct answers mentioned	
**Family planning (9 questions)**		Maximum possible score: 9
How soon after you give birth can you get pregnant again?		
What is the recommended length of time you should wait between having this child and getting pregnant with another child?		
What are benefits of using family planning?	Unprompted free list, enumerator noted any correct answers mentioned	
Which ways or methods contraception have you heard about?	Unprompted free list, enumerator noted any correct answers mentioned	
True/False: There are many safe methods of birth control	True / false / don’t know	True

Questions	Answer options	Correct answer(s)
True/False: Female sterilization can be done at the time of giving birth	True / false / don't know	True
True/False: Male sterilization is an easy way to control family size	True / false / don't know	True
True/False: Men become physically weak after accepting male sterilization	True / false / don't know	False
True/False: PPIUCD insertion and female sterilization services can be free of cost at government facilities	True / false / don't know	True

Supplementary Table 2: Factors associated with pregnant women's RMNCH&N knowledge by domain in four districts of Madhya Pradesh, India

Variable	Mean composite score	Maternal nutrition and pregnancy danger signs	Family Planning	Infant and young child care	Immediate post-birth care for newborns	Infant and young child feeding
**Age**						
18-24	Reference					
25-34	1.91(1.35-2.46)*	1.95(1.02-2.87)*	1.92(1.18-2.67)*	1.85(1.11-2.58)*	2.32(1.1-3.54)*	1.51(0.55-2.47)*
35+	2.04(0.34-3.74)*	2.19(-0.52-4.9)	4.67(2.06-7.29)*	0.81(-1.33-2.95)	1.53(-2.42-5.47)	1.01(-2.36-4.38)
**Parity (>=1 child)**	4.39(3.74-5.03)*	3.29(2.33-4.26)*	1.74(0.92-2.56)*	5.93(5.13-6.74)*	6.8(5.46-8.14)*	4.18(3.11-5.25)*
**Caste**						
General	Reference					
Other backwards	0.62(-0.06-1.3)	0.51(-0.63-1.65)	0.07(-0.83-0.96)	0.82(-0.04-1.68)	1.94(0.47-3.41)*	-0.25(-1.41-0.91)
Scheduled	0.24(-0.58-1.06)	0.12(-1.2-1.43)	0.35(-0.71-1.4)	-0.04(-1.05-0.96)	1.08(-0.63-2.79)	-0.31(-1.65-1.04)
**Wealth Index**						
Q1	Reference					
Q2	0.32(-0.49-1.13)	-0.61(-1.99-0.78)	1.33(0.23-2.43)*	0.67(-0.4-1.75)	0.66(-1.01-2.33)	-0.47(-1.85-0.9)
Q3	0.14(-0.78-1.07)	0.17(-1.37-1.71)	1.17(-0.04-2.37)	0.5(-0.69-1.7)	-1.24(-3.06-0.57)	0.11(-1.44-1.66)
Q4	0.16(-0.83-1.16)	-0.02(-1.65-1.61)	0.95(-0.33-2.24)	0.85(-0.41-2.1)	-2.32(-4.28--0.36)*	1.35(-0.3-2.99)
Q5	0.72(-0.38-1.82)	1.8(-0.01-3.6)	2.32(0.93-3.72)*	1.21(-0.19-2.62)	-2.95(-5.22--0.68)*	1.24(-0.55-3.03)
**Education**						

For peer review only

No School	Reference					
Primary or Less	0.65(-0.35-1.65)	1.87(0.24-3.5)*	1.57(0.2-2.95)*	0.01(-1.34-1.36)	-1.55(-3.6-0.5)	1.35(-0.43-3.13)
Middle School	0.6(-0.51-1.72)	1.19(-0.56-2.93)	2.43(0.92-3.94)*	0.14(-1.27-1.54)	-1.78(-4.06-0.51)	1.04(-0.83-2.91)
Higher Education	1.65(0.42-2.89)*	3.42(1.51-5.34)*	3.29(1.63-4.94)*	1.5(-0.03-3.03)	-2.82(-5.41--0.24)*	2.87(0.83-4.91)*
**Literacy**						
Cannot read at all	Reference					
Reading only parts of sentence	0.95(0.01-1.9)*	0.93(-0.57-2.42)	0.88(-0.42-2.18)	0.91(-0.33-2.15)	0.33(-1.64-2.31)	1.7(0.05-3.34)*
Read whole sentence	2.29(1.42-3.15)*	3.12(1.78-4.46)*	2.29(1.17-3.41)*	1.4(0.33-2.47)*	1.86(0.11-3.61)*	2.76(1.32-4.19)*
**Self Help Group**	0.4(-0.42-1.22)	0.88(-0.57-2.34)	0.77(-0.46-2.01)	0.45(-0.71-1.61)	-0.18(-2.33-1.98)	0.08(-1.39-1.54)
**Nuclear Family**	-2.14(-2.95--1.33)*	-2.61(-3.83--1.38)*	-1.7(-2.65--0.75)*	-4.38(-5.39--3.38)*	-1.46(-2.92-0)	-0.57(-1.81-0.67)
**Primary decision maker in health decisions**	1.59(1-2.19)*	2.74(1.75-3.74)*	0.48(-0.32-1.27)	2.61(1.88-3.34)*	0.43(-0.8-1.66)	1.71(0.68-2.74)*
**Number of ANC Visits**						
1	Reference					
2	1.03(0.16-1.9)*	4.19(2.8-5.59)*	0.62(-0.62-1.86)	-0.03(-1.15-1.08)	0.18(-1.59-1.95)	0.17(-1.24-1.58)
3	1.95(1.04-2.85)*	5.91(4.37-7.45)*	0.35(-0.94-1.63)	0.65(-0.51-1.81)	1.38(-0.49-3.25)	1.44(-0.05-2.93)
4	1.68(0.81-2.54)*	6.61(5.13-8.1)*	0.19(-1.01-1.4)	0.43(-0.67-1.54)	0.12(-1.67-1.9)	1.02(-0.42-2.46)
**Satisfied with the services provided by the ASHA**	2.18(1.57-2.79)*	2.78(1.83-3.72)*	2.06(1.24-2.87)*	1.7(0.9-2.5)*	2.93(1.72-4.13)*	1.44(0.42-2.46)*
**Phone Ownership**	0.61(-0.01-1.22)	0.82(-0.2-1.85)	0.71(-0.11-1.52)	0.73(-0.1-1.57)	0.4(-0.82-1.63)	0.37(-0.67-1.4)
**District**						
Hoshangabad	Reference					
Mandsaur	-3.3(-4.51--2.09)*	-1.72(-3.54-0.1)	-1.49(-2.98-0)	-2.14(-3.59--0.7)*	-6.98(-9.75--4.21)*	-4.18(-5.98--2.38)*
Rewa	-0.13(-1.21-0.95)	-3.05(-4.78--1.31)*	-0.85(-2.24-0.54)	-0.69(-2.02-0.63)	3.58(1.18-5.98)*	0.36(-1.31-2.03)
Rajgarh	-3.05(-4.21--1.89)*	-6.59(-8.41--4.78)*	-1.26(-2.76-0.24)	-0.26(-1.68-1.16)	-4.35(-6.92--1.78)*	-2.77(-4.53--1.01)*

*= Significant at 0.05 level

For peer review only

Supplementary Table 3: Measurement of degree to which repeated measurements in pregnant women interviewed provide similar answers

Question text	Response Options	Baseline Prevalence	Retest Prevalence	Phone Prevalence	Retest Kappa	Phone Kappa
**Domain: Maternal nutrition and pregnancy danger signs**						
What foods should you eat during pregnancy?	Fish	0.07	0.08	0.02	0.79	0.84
	Meat	0.08	0.06	0.04	0.80	0.83
	Eggs	0.13	0.08	0.05	0.79	0.68
	Milk/Dairy Products	0.73	0.79	0.59	0.32	0.17
What should you not eat or drink during your pregnancy?	Alcohol	0.03	0.02	0.01	0.94	0.93
	Coffee	0.03	0.01	0.05	0.95	0.84
	Cigarettes	0.05	0.02	0.01	0.95	0.83
	Tea	0.14	0.16	0.09	0.58	0.45
Anaemia (khoon ki kami) in pregnancy can affect the growth and development. What should you do if you are anemic?	Take IFA tablet daily	0.64	0.73	0.56	0.30	0.21
How many tetanus injections should you have during pregnancy?	2 shots	0.66	0.52	0.65	0.77	0.58
What danger signs during pregnancy and before labor starts would lead you to go to the health facility immediately?	Yellowing of skin	0.10	0.02	0.01	0.88	0.78
**Domain: Infant and young child feeding**						
How many times per day should newborns be breastfed?	9-10 times a day, on demand	0.94	0.94	N/A	0.75	N/A
What are some of the benefits of breastfeeding?	It helps to maintain space between children	0.01	0.01	0.01	0.98	1.00
	Reduces expenditure on medical care as child will fall sick less number of times	0.11	0.05	0.05	0.82	0.62
	The more the child breastfeeds, the more milk will be produced	0.07	0.09	0.02	0.67	0.91
How soon after delivery should you give foods or liquids other than mothers milk?	6 months	0.83	0.74	0.75	0.64	0.64
	Cheese	0.05	0.07	0.01	0.73	0.85

For peer review only

What types of foods should a baby be given after 6 months of age?	White potatoes, roots	0.01	0.00	0.09	0.99	0.83
	Ripe mangoes, papayas	0.05	0.10	0.05	0.71	0.82
	Beans, peas, nuts	0.14	0.08	0.02	0.75	0.52
	Bread, roti, grains	0.17	0.08	0.14	0.55	0.54
	Fruits or vegetables	0.14	0.16	0.16	0.52	0.63
	Baby food	0.42	0.39	0.47	0.05	0.07
**Domain: Infant and young child care**						
What danger signs do you know about for the newborn after delivery?	Redness	0.01	0.01	0.01	0.98	0.99
	Red eyes	0.02	0.02	0.01	0.94	0.96
	Skin lesions	0.03	0.02	0.01	0.87	0.96
	Blueness of hands	0.03	0.01	0.01	0.96	0.91
	Convulsions	0.04	0.02	0.06	0.85	0.84
	Low birth weight	0.05	0.05	0.04	0.83	0.74
	Lethargy	0.09	0.17	0.06	0.51	0.74
	Difficulty feeding	0.22	0.17	0.16	0.50	0.28
	Yellow color of skin	0.17	0.23	0.04	0.50	0.61
	Pitched cry	0.26	0.21	0.30	0.44	0.20
How soon after your baby is born should it receive its first vaccination?	At Birth	0.74	0.80	0.73	0.52	0.54
	Within 1 month	0.09	0.11	0.09		
	1-2 months	0.04	0.01	0.02		
	2+ months	0.02	0.01	0.02		
What are things you can do to prevent your child from getting diarrhea?	Give baby safe drinking water > 6 months	0.17	0.08	0.05	0.68	0.65
	Exclusively breastfeed children < 6 months	0.14	0.06	0.05	0.69	0.63
	Cover water and food to avoid flies sitting on it	0.16	0.23	0.10	0.39	0.55
	Safe disposal of stools	0.09	0.11	0.12	0.57	0.49
	Make sure the environment is clean	0.50	0.63	0.38	0.02	-0.02
What should you give your child to treat diarrhea?	Intravenous	0.01	0.01	0.01	0.96	0.98
	Antibiotic	0.03	0.00	0.01	0.96	0.95

	Injection	0.07	0.18	0.01	0.50	0.80
	Home remedy	0.10	0.02	0.01	0.93	0.78
	Salt and sugar	0.06	0.04	0.13	0.86	0.71
	ORS + Zinc	0.11	0.11	0.18	0.70	0.50
	ORS	0.40	0.48	0.27	0.38	0.24
**Domain: Family Planning**						
How soon after you give birth, can you get pregnant again?	Immediately	0.02	0.01	0.04	0.43	-0.02
	Not until menses return	0.73	0.80	0.12		
	After you stop breastfeeding	0.03	0.01	0.14		
What is the recommended length of time you should wait between having another child?	Immediately	0.00	0.00	0.00	0.61	0.36
	Wait for at least 1 year	0.02	0.02	0.02		
	Wait for at least 2 years	0.19	0.27	0.18		
	Wait for at least 3 years	0.67	0.64	0.51		
What are the benefits of using family planning to limit the size of your family?	Easy way to control the size of your family	0.48	0.47	0.46	0.02	0.00
	Financial savings	0.50	0.51	0.50	0.01	0.06
Men become physically weak after accepting sterilization	FALSE	0.15	0.14	0.09	0.28	-0.02
**Domain: Essential newborn care**						
How do you keep the baby warm after delivery?	Put the baby on your chest	0.17	0.15	0.13	0.50	0.49
	Dried or wiped soon after birth	0.25	0.30	0.24	0.08	0.24
What should you put on the cord after delivery?	Nothing	0.06	0.05	0.04	0.82	0.77
How soon after delivery should your baby be bathed?	1 Day	0.80	0.77	0.03	0.49	0.80

Supplementary Table 4: Low Reliability Questions

Question text	Response Options	Baseline Prevalence	Retest Prevalence	Phone Prevalence
**Domain: Maternal nutrition and danger signs**				

For peer review only

What foods should you eat during pregnancy?	Fruits	0.89	0.92	0.73
	Green leafy vegetables	0.94	0.93	0.76
	Pulses and Nuts	0.86	0.88	0.34
What are Iron Folic Acid tablets?	Help prevent/treat anemia	0.80	0.90	0.64
	Improve the health/well-being of my baby	0.73	0.85	0.43
What danger signs during pregnancy and before labor starts would lead you to immediately go to the health center?	Vaginal Bleeding	0.31	0.32	0.10
	Vaginal Discharge	0.35	0.33	0.10
	Convulsions	0.42	0.52	0.09
	Stomach Cramps	0.87	0.95	0.45
	Swelling on limbs and face	0.38	0.30	0.20
Anaemia (khoon ki kami) in pregnancy can affect the growth and development. What should you do if you are anemic?	Tea	0.40	0.63	0.07
	Cigarettes/bids	0.83	0.76	0.34
**Domain: Infant and young child feeding**				
What are some of the benefits of breastfeeding?	Promotes Mother-Baby bonding	0.09	0.01	0.27
	Help build immunity for your child	0.72	0.71	0.57
	Promotes child growth, wellness	0.85	0.96	0.54
How soon after delivery should you breastfeed your child?	Immediately	0.28	0.64	0.15
What types of foods should a baby be given after 6 months of age?	Plain Water	0.74	0.76	0.37
	Juice	0.10	0.07	0.35
	Lentil Broth/soup	0.18	0.14	0.73
	Milk	0.78	0.90	0.52
	Pumpkin, squash	0.78	0.88	0.38

For peer review only

	Solid, semi-solid, soft food	0.27	0.45	0.00
	Liver	0.00		0.00
	Chicken	0.00		0.00
	Meat	0.00		0.00
	Eggs	0.00		0.00
	Dried Fish	0.00		0.00
**Domain: Infant and young child care**				
What danger signs do you know about for the newborn after delivery that would cause you to go to the health center?	Difficulty breathing	0.36	0.35	0.05
	Baby feels hot or cold to touch	0.30	0.49	0.15
	Fever	0.83	0.93	0.34
	Vomiting	0.52	0.49	0.24
What are things you can do to prevent your child from getting diarrhea?	Wash hands	0.39	0.40	0.20
What should you give your child to treat diarrhea?	Antidiarrheals	0.39	0.41	0.05
	Other pill or syrup	0.62	0.69	0.38
What are three critical times for a woman to wash her hands?	After defecation	0.98	0.94	0.43
	Before cooking or handling food	0.91	0.94	0.37
	Before eating or feeding the child	0.89	0.92	0.37
**Domain: Family Planning**				
What are the benefits of using family planning to limit the size of your family?	Give you more time to take care of the children you already have	0.70	0.82	0.54
Which ways or methods of contraception have you heard about?	Female sterilization	0.92	0.97	0.37
	Male Sterilization	0.49	0.54	0.21
	IUD	0.47	0.36	0.31
	Oral contraceptive pills	0.84	0.93	0.58

For peer review only

	Injectables	0.70	0.78	0.28
	Male Condom	0.64	0.74	0.46
	Rhythm Method	0.56	0.45	0.02
	Withdrawl	0.24	0.08	0.05
There are many safe methods of birth control	TRUE	0.94	0.96	0.33
Female sterilization can be done at the time of birth	TRUE	0.76	0.82	0.21
Male sterilization is an easy way to control family size	TRUE	0.72	0.63	0.22
PPIUCD insertion and female sterilization	TRUE	0.87	0.86	0.35
Do you know of a place where you can obtain a method of family planning?	Yes	0.90	0.98	0.38
**Domain: Immediate post-birth care for the neonate**				
How do you keep the baby warm after delivery?	Cover in clothes	0.96	1.00	0.56
What should you use to cut the umbilical cord?	New Razor blades	0.72	0.71	0.11
	Blade used for other purposes	0.72		0.05
	Scissors	0.41	0.72	0.29
	Knife	0.02	0.03	0.18
	Surgical Blade	0.03	0.01	0.39

For peer review only

BMJ Open

Assessing the reliability of phone surveys to measure reproductive, maternal, and child health knowledge among pregnant women in rural India: a feasibility study

Journal:	*BMJ Open*
Manuscript ID	bmjopen-2021-056076.R1
Article Type:	Original research
Date Submitted by the Author:	15-Nov-2021
Complete List of Authors:	Ng, Angela; Johns Hopkins University Bloomberg School of Public Health<br>Mohan, Diwakar; Johns Hopkins University Bloomberg School of Public Health<br>Shah, Neha; Johns Hopkins University Bloomberg School of Public Health<br>Scott, Kerry; Johns Hopkins University Bloomberg School of Public Health<br>Ummer, Osama; Oxford Policy Management; BBC Media Action,<br>Chamberlain, Sara; BBC Media Action, BBC Media Action, India; BBC Media Action, Asia<br>Bhatnagar, Aarushi; World Bank New Delhi Office, Health, Nutrition and Population<br>Dhar, Diva; Bill & Melinda Gates Foundation<br>Agarwal , Smisha ; Johns Hopkins University Bloomberg School of Public Health, Department of International Health<br>Ved, Rajani; National Health Systems Resource Centre<br>LeFevre, Amnesty; Johns Hopkins University Bloomberg School of Public Health, International Health
**Primary Subject Heading**:	Global health
Secondary Subject Heading:	General practice / Family practice, Paediatrics
Keywords:	Community child health < PAEDIATRICS, Maternal medicine < OBSTETRICS, Neonatal intensive & critical care < INTENSIVE & CRITICAL CARE

SCHOLARONE™
Manuscripts

I, the Submitting Author has the right to grant and does grant on behalf of all authors of the Work (as defined in the below author licence), an exclusive licence and/or a non-exclusive licence for contributions from authors who are: i) UK Crown employees; ii) where BMJ has agreed a CC-BY licence shall apply, and/or iii) in accordance with the terms applicable for US Federal Government officers or employees acting as part of their official duties; on a worldwide, perpetual, irrevocable, royalty-free basis to BMJ Publishing Group Ltd ("BMJ") its licensees and where the relevant Journal is co-owned by BMJ to the co-owners of the Journal, to publish the Work in this journal and any other BMJ products and to exploit all rights, as set out in our licence.

The Submitting Author accepts and understands that any supply made under these terms is made by BMJ to the Submitting Author unless you are acting as an employee on behalf of your employer or a postgraduate student of an affiliated institution which is paying any applicable article publishing charge ("APC") for Open Access articles. Where the Submitting Author wishes to make the Work available on an Open Access basis (and intends to pay the relevant APC), the terms of reuse of such Open Access shall be governed by a Creative Commons licence – details of these licences and which Creative Commons licence will apply to this Work are set out in our licence referred to above.

Other than as permitted in any relevant BMJ Author's Self Archiving Policies, I confirm this Work has not been accepted for publication elsewhere, is not being considered for publication elsewhere and does not duplicate material already published. I confirm all authors consent to publication of this Work and authorise the granting of this licence.

For peer review only

**Title**	Assessing the reliability of phone surveys to measure reproductive, maternal, and child health knowledge among pregnant women in rural India: a feasibility study
**Author's Names**	Angela Ng, Diwakar Mohan (0000-0002-7532-366X), Neha Shah, Kerry Scott (0000-0003-3597-9637), Osama Ummer (0000-0002-4189-5328), Sara. Chamberlain (0000-0003-4785-6482), Aarushi Bhatnagar, Diva Dhar, Smisha Agarwal, Rajani Ved, Amnesty E. LeFevre (0000-0001-8437-7240), on behalf of the Kilkari Impact Evaluation Team*
**Address for each author**	**Angela Ng MSPH**<br>Department of International Health, Johns Hopkins Bloomberg School of Public Health, 615 N. Wolfe St, Baltimore, Maryland, USA<br>angelang1201@gmail.com<br><br>**Diwakar Mohan DrPH MD**<br>Title: Assistant Scientist<br>Department of International Health, Johns Hopkins Bloomberg School of Public Health, 615 N. Wolfe St, Baltimore, Maryland, USA<br>dmohan3@jhu.edu<br>ORCiD iD: 0000-0002-7532-366X<br><br>**Neha Shah MSPH**<br>Title: Research Associate<br>Department of International Health, Johns Hopkins Bloomberg School of Public Health, 615 N. Wolfe St, Baltimore, Maryland, USA<br>nshah67@jhu.edu<br><br>**Kerry Scott PhD, MSc**<br>Title: Associate<br>Department of International Health, Johns Hopkins Bloomberg School of Public Health, 615 N. Wolfe St, Baltimore, Maryland, USA<br>kscott26@jhu.edu<br>ORCiD iD: 0000-0003-3597-9637<br><br>**Osama Ummer MHA, BAMS**<br>Title: Research Manager<br>(1) BBC Media Action, India<br>Innov8 Old Fort Saket District Mall, Saket District Centre, Sector 6, Pushp Vihar, New Delhi, Delhi 110017, India<br>(2) Oxford Policy Management-Delhi, 4/6 First Floor, Siri Fort Institutional Area, New Delhi, Delhi 110049, India<br>kposamaummer@gmail.com<br>ORCiD iD: 0000-0002-4189-5328<br><br>**Sara Chamberlain, MA**<br>Title: Digital Director, BBC Media Action, India<br>Innov8 Old Fort Saket District Mall, Saket District Centre, Sector 6, Pushp Vihar, New Delhi, Delhi 110017, India<br>sara.chamberlain@in.bbcmediaaction.org<br>ORCiD iD: 0000-0003-4785-6482<br><br>**Aarushi Bhatnagar**<br>Title: Health Economist<br>Oxford Policy Management-Delhi, 4/6 First Floor, Siri Fort Institutional Area, New Delhi, Delhi 110049, India<br>aarushibhatnagar@gmail.com<br><br>**Diva Dhar**

For peer review only

	Title: Senior Program Officer, Gender Data & Evidence<br>Bill and Melinda Gates Foundation, 500 5th Ave N, Seattle, WA 98109 USA<br>Diva.Dhar@gatesfoundation.org<br><br>**Smisha Agarwal**<br>Title: Assistant Professor<br>Department of International Health, Johns Hopkins Bloomberg School of Public Health, 615 N. Wolfe St, Baltimore, Maryland, USA<br>sagarw23@jhu.edu<br><br>**Rajani Ved**<br>Title: Executive Director<br>National Health Systems Resource Centre, Baba Gangnath Marg, Munirka, New Delhi 110067<br>rajani.ved@gmail.com<br><br>**Amnesty LeFevre PhD, MHS**<br>Title: Associate Professor<br>1. School of Public Health and Family Medicine, University of Cape Town, Cape Town, South Africa<br>2. Department of International Health, Johns Hopkins Bloomberg School of Public Health, 615 N. Wolfe St, Baltimore, Maryland, USA<br>Email: aelefevre@gmail.com<br>ORCiD iD: 0000-0001-8437-7240<br><br>On behalf of the Kilkari Impact Evaluation Team<br>*(listed in Alphabetical order) Smisha Agarwal, Salil Arora, Jean JH Bashingwa, Aarushi Bhatanagar, Sara Chamberlain, Rakesh Chandra, Arpita Chakraborty, Neha Dumke, Priyanka Dutt, Anna Godfrey, Suresh Gopalakrishnan, Nayan Kumar, Simone Honikman, Alain Labrique, Amnesty LeFevre, Jai Mendiratta, Molly Miller, Radharani Mitra, Diwakar Mohan, Deshen Moodley, Nicola Mulder, Angela Ng, Dilip Parida, Nehru Penugonda, Sai Rahul, Shiv Rajput, Neha Shah, Kerry Scott, Aashaka Shinde, Aaditya Singh, Nicki Tiffin, Osama Ummer, Rajani Ved, Falyn Weiss, Sonia Whitehead
**Contributions**	AEL is the overall study PI and conceived the idea for this study along with DM, AB, RV, KS, and SC. Data collection tools were designed by AL, NS, DM, KS, AB, SA, OU, and RV with inputs from DD. Phone survey data collection was led and supervised by NS and RV. Face-to-face data collection was supervised by AB, OU, AEL, DM and AL. AN and NS led the analyses with oversight from DM and inputs from AL. AN and AL wrote the manuscripts with edits and inputs from all co-authors. AN generated all final tables and figures.
**Keywords**	Phone Surveys, Knowledge, Pregnant Women, India, Madhya Pradesh
**Corresponding Author**	**Angela Ng, MSPH**<br>Department of International Health, Johns Hopkins Bloomberg School of Public Health, 615 N. Wolfe St, Baltimore, Maryland, USA<br>angelang1201@gmail.com

Copyright: The Corresponding Author has the right to grant on behalf of all authors and does grant on behalf of all authors, an exclusive licence on a worldwide basis to the BMJ Publishing Group Ltd to permit this article (if accepted) to be published in BMJ editions and any other BMJPGL products and sublicences such use and exploit all subsidiary rights, as set out in our licence.

Competing interests: All authors have completed the Unified Competing Interest form (available on request from the corresponding author) and declare that the research reported was funded by the Bill and Melinda Gates Foundation. AG, SC, PD are employed by BBC Media Action; one of the entities supporting program implementation. The authors do not have other relationships and are not engaged in activities that could appear to have influenced the submitted work.

Transparency declaration: I, Angela Ng, affirm that the manuscript is an honest, accurate, and transparent account of the study being reported; that no important aspects of the study have been omitted; and that any discrepancies from the study as planned (and, if relevant) have been explained.

Ethics: Institutional Review Boards from the Johns Hopkins Bloomberg School of Public Health in Baltimore, Maryland USA and Sigma Research and Consulting in Delhi, India provided ethical clearance for study activities. Verbal informed consent was obtained from all study participants.

Funding: Bill and Melinda Gates Foundation Role of study sponsors: The study sponsors had no role in the design, implementation or interpretation of study findings. The study team were independent from the funders.

Data Sharing Statement: Extra data can be found by emailing Amnesty LeFevre at aelefevre@gmail.com

For peer review only

Abstract (298 of 300)

Objectives: Efforts to understand the factors influencing the uptake of reproductive, maternal and child health, and nutrition (RMNCH&N) services in high disease burden low-resource settings have often focused on face-to-face surveys or direct observations of service delivery. Increasing access to mobile phones has led to growing interest in phone surveys as a rapid, low-cost alternatives to face-to-face surveys. We assess determinants of RMNCH&N knowledge among pregnant women with access to phones and examine the reliability of alternative modalities of survey delivery.

Participants: Women 5-7 month's pregnant with access to a phone

Setting: Four districts of Madhya Pradesh, India

Design: Cross-sectional surveys administered face-to-face and within two weeks, the same surveys were repeated among two random sub-samples of the original sample: face-to-face (n=205), and caller attended telephone interviews (n=375). Bivariate analyses, multivariable linear regression, and prevalence and bias-adjusted kappa scores are presented.

Results: Knowledge scores were low across domains: 52% for maternal nutrition and pregnancy danger signs, 58% for family planning, 47% for essential newborn care, 56% infant and young child feeding, and 58% for infant and young child care. Higher knowledge (>=1 composite score) was associated with older age; higher levels of education and literacy; nuclear family accommodations; primary health decision making; greater attendance in antenatal care; and satisfaction with ASHA services. Surveys questions had low interrater and intermodal reliability (K<.70) with few exceptions. Questions with low reliability included True/false questions and those with unprompted, multiple response options. Reliability may have been hampered by the sensitivity of the content, lack of privacy, enumerators and respondents profile differences, rapport, social desirability bias, and/or enumerator's ability to adequately convey concepts or probe.

Conclusions: Phone surveys are a reliable modality for generating population level estimates data about pregnant women's knowledge, however, should not be used for individual level tracking.

ARTICLE SUMMARY

Strengths and limitations of this study:

Strengths:

- COVID-19 has accelerated the use of remote data capture strategies, however, few studies have undertaken a rigorous process of tool development inclusive of reliability testing.
- Study findings suggest that phone surveys can be a reliable modality for measuring knowledge among pregnant women in rural India at a population level.
- To optimize reliability, limit the use of questions which are sensitive, or have open-ended or multi-response options.

Limitations:

- The sample included pregnant women with reported access to a phone during the day; a population arguably more advantaged than women without access to a phone which could limit the generalizability of findings.

INTRODUCTION

COVID-19 has accelerated the demand for remote data capture solutions, including phone surveys. There is a growing importance and reliance on remote data collection including phone surveys (in India and elsewhere) as COVID has impacted the ease and budgets for data collection (1). Recommendations on questions to consider in determining the suitability of phone surveys to measure varied outcomes is emerging (2). However, despite an increasing body of literature on methods (3,4), including sampling (5, 6), ethics and obtaining consent (5,7,8), improving response rates and other performance metrics (9), significant gaps persist in the standards for developing phone survey tools.

Efforts to measure health practice, prevention, and careseeking in India have most commonly been done via face-to-face survey methods, including the population-based National Family Health Surveys (NFHS) carried out approximately every 5 to 7 years (10,11). In addition to being costly and time intensive, these population based surveys are limited by lengthy recall bias (12). Phone surveys have the potential to be administered more proximally to event of focus, at lower cost, with more minimal resources, and with potentially less burden to the beneficiary.

In 2015-16, NFHS survey findings suggested that household access to mobile phones in India is near universal (94% urban and 80% rural) and nearly half (48%) of women have a mobile phone that they themselves use (13). Growing access to mobile phones at a household level, and more specifically among women, provides an opportunity to develop alternative modalities for the routine collection of data on health behaviors and knowledge. Despite their immense potential, few applications of phone surveys have been reported for measuring health knowledge, behaviors and outcomes in India (11) and even less information is available on the methods influencing the development of these tools. As phone ownership and access increase in India (14), a reliable phone survey tool would enable timely, routine and low cost measurement of the population health status, including knowledge, careseeking, and practices (15).

In this manuscript, we outline the process for developing and refining a phone survey tool for the measurement of reproductive, maternal and newborn health and nutrition (RMNCH&N) knowledge among pregnant women in four districts of Madhya Pradesh. We start by developing overall and domain-specific RMNCH&N knowledge scores and then identify the determinants of higher knowledge scores (Figure 1). We then assess differences in the reliability of the knowledge questions over different modalities including face-to-face surveys at two time points (hereafter called test-retest) and caller attended telephone interviews (CATI; hereafter called phone surveys). This study illuminates gaps in RMNCH&N knowledge among pregnant women, determinants of RMNCH&N knowledge levels, and the reliability of phone survey tools compared to face-to-face tools.

METHODS

Study Setting

Data collection activities were carried out from August – October 2018 in four districts (Hoshangabad, Mandsaur, Rewa, and Rajgarh) of Madhya Pradesh. Madhya Pradesh is a state in central India with a population of over 75 million, predominantly Hindu, and based in rural areas (16). Significant gaps between men and women persist with regard to literacy (82% of men are literate as compared to 59% of women), and only 29% of women report having access to mobile phones that they themselves can use (17). For nearly all health indicators, Madhya Pradesh falls below national averages. Despite near universal attendance of at least one antenatal care visit during pregnancy, only 53% of women receive services within the first trimester, and 36% receive the recommended four visits (17). While institutional delivery rates have increased over time, 20% of women deliver outside the formal health sector, and only 18% receive a health check after birth from a trained provider within 2 days of birth (17). Among children, 51 per 1,000 live births will die prior to their first birthday and 65 per 1,000 live births will die prior to their fifth birthday (17). High mortality rates are affected by undernutrition (43% of children are under weight, 42% are

For peer review only

stunted), low rates of exclusive breastfeeding (58%), and gaps in access to basic health services including immunization. Where the latter is concerned, only 73% of children 12-23 months have received the recommended 3 doses of the DPT vaccine (17).

Study design and sampling

The target population was women 5-7 months pregnant at randomization, ≥ 18 years of age, speak and understand Hindi, and own or have access to a mobile phone during the morning and afternoon. Survey data and sampling technique were captured as part of baseline survey activities conducted as part of the impact evaluation of the Kilkari mobile health intervention described in detail elsewhere (18). A face-to-face cross-sectional survey was conducted among (n=5,095) women with reported access to a mobile phone during the day (Survey Activity A in Figure 1). The survey sample size was powered to detect a 5% change in the practice of exclusive breastfeeding from baseline to endline (12 months later), assuming an alpha of 0.05, power of 0.80, 20% loss to follow-up, and 35% loss of women due to changes in mobile phone numbers (SIM change). Women excluded included BSNL mobile subscribers due to poor network coverage and those who did not provide consent to participate in the trial. Front line health workers in the study villages and census of villages produced the list of eligible women. The sample was randomized and stratified by gestational age, parity, age of woman, and ownership of phone in stat using the sample command (19).

Within two weeks of the original survey, two separate surveys were conducted on random samples of these women: test-retest (n=205), and phone surveys (n=375). To assess reproducibility in the surveys, stability of the instrument, and inter-rater reliability, a subsample of women who completed the main survey was re-interviewed face-to-face to assess the inter-rater reliability of the responses from the main survey (Survey Activity B in Figure 1). In order to develop a validated phone survey tool, pregnant women who previously completed a face-to-face interview and were not interviewed in the test-retest phase were interviewed a second time over the phone to assess the inter-modal reliability of the responses from the main survey (Survey Activity C in Figure 1).

Each sample size was calculated assuming a kappa of 0.80, margin of error 0.05, and alpha of 0.5, proportion of positive responses of 0.35 for rater 1 and 0.40 for rater 2: 146 participants were needed. To accommodate a 15% loss to follow up or refusal for the test-retest, a minimum of 168 women were targeted and ultimately 205 interviewed. Assuming a 20% response rate for the phone survey, a random sample of 880 women were contacted within 1-2 weeks of the initial face-to-face interview, and ultimately 375 completed the interviews.

Data collection

The survey included seven modules on: 1. mobile phone ownership, use, and digital literacy; 2. pregnancy, delivery, child health and family planning knowledge; 3. birth history; 4. experiences during pregnancy care; 5. content of pregnancy care; 6. delivery and postpartum care intentions; and 7. client satisfaction with accredited social health activists (ASHA) services. Survey questions were drawn from standardised national survey tools, the literature and expert judgment and enhanced through cognitive testing and piloting (18, 20). This manuscript focuses on knowledge; findings from other modules are reported elsewhere (18, 21).

Female enumerators conducted face-to-face and phone surveys. The phone surveys were conducted out of the National Health Systems Resource Center in New Delhi. Surveyors had a minimum of high school education and received training on tool content, and survey implementation, including informed consent. Supervisors provided day to day support and oversight.

Data analysis

For peer review only

Data were analyzed using Stata 15 (Stata Corporation, College Station, TX, USA). First, a conceptual framework was developed comprised of factors believed to influence pregnant women's knowledge from previous literature (Figure 2) (18-35). At the individual level, characteristics such as phone ownership, health care characteristics, socioeconomic, and sociodemographic factors were considered. A wealth index was created by dividing the women into 5 quintiles using principal component analysis. At the community level, social gender norms were analyzed such as participation in self-help groups, women being the primary health decision maker, and family living structure as well as geographic characteristics of the community.

In order to best categorize knowledge among pregnant women, survey questions were consolidated into five thematic domains: 1. Maternal nutrition and pregnancy danger signs, 2. Family planning, 3. Essential newborn care, 4. Infant and young child feeding, and 5. Infant and young child care (Supplementary Table 1). For each question, if there was only one correct answer, a binary score was given: 1 if the women knew the correct answer, 0 if not. If there were multiple correct answer options, each correct option was weighted so if all correct options were selected, the final score for that question would be 1. Each question then had equal weight in its category. All the knowledge domains were averaged together at the end to create a final composite score.

A simple linear regression was performed on the final composite score with the factors thought to have an impact on them. A multiple linear regression was performed to see all together which factors would have an impact on overall women's knowledge. In order to find the best model, only variables with a p-value of 0.20 or less during bivariate analyses were used. A multiple linear regression was also performed on each individual knowledge domain to see if particular factors influenced one category more than another.

If the difference in prevalence between survey questions was <15%, kappa scores were used to assess the reliability of the question. Questions were deemed unreliable if the difference in prevalence between survey questions was >15% or if the Cronbach's alpha was 0.7 or higher (22). Kappa coefficients were adjusted for the difference in prevalence levels using the Prevalence Adjusted Bias Adjusted Kappa (PABAK) score. This was to determine if the questions themselves were reliable, and if so, if the question was reliable over the phone. If the threshold of 0.7 was met for just the test-retest, the question was deemed to be reliable for face-to-face surveys in this context. If the threshold of 0.7 was met for both test-retest and phone survey, the question was deemed reliable for phone surveys in this context.

Patient and public involvement

Pregnant women were first engaged upon identification in their households as part of a household listing carried out in mid/late 2018. Those meeting eligibility criteria were interviewed as part of the baseline survey. A further sub-sample of these were interviewed as part of reliability surveys, including caller attended telephone interviews. Prior to the administration of surveys, a small number of pregnant women were involved in the refinement of survey tools through qualitative interviews, including cognitive interviews, which were carried out to optimise survey questions, including the language and translation used. COVID-19 and associated travel restrictions prevented further engagement with women interviewed to disseminate of study findings.

Ethics Approval

Ethical approval for this research study was obtained through Johns Hopkins School of Public Health Institutional Review Board in Baltimore, Maryland, as well as Sigma Research and Consulting in New Delhi, India. All participants consented to the study prior to interview.

RESULTS

Pregnant Women Characteristics

For peer review only

Among 5,095 interviewed through the face-to-face survey, 95% were Hindu, 58% were between 18-24 years of age, 68% had at least one child, 47% belonged to other backward castes, 8% belong to a self-help group, 41% had completed high school or higher, and 57% were able to read the whole sentence (Table 1). A majority of the women interviewed resided in Rewa (57%), followed by Mandsaur, Hoshangabad, and Rajgarh falling at 16%, 8%, and 19% respectively.

Table 1. Characteristics of pregnant women drawn from a face-to-face survey in 4 districts of Madhya Pradesh.

Characteristic	n	Frequency
**Age**		
18-24	2,975	58.4%
25-34	2,023	39.7%
35+	97	1.90%
**Religion**		
Hindu	4,848	95.2%
Muslim	241	4.73%
Other	6	0.12%
**Caste**		
General	1,133	22.2%
Other Backward Caste	2,386	46.8%
Scheduled Caste/Tribe	1,576	30.9%
**Parity (>=1 Child)**	3,486	68.4%
**Employed**	1,921	37.7%
**Education**		
No School	548	10.8%
Primary or less	876	17.2%
Middle School	1,560	30.6%
High School	1,666	32.7%
Higher Education	445	8.70%
**Literacy**		
Cannot read at all	1,659	32.6%
Able to read only parts of sentence	554	10.9%
Able to read whole sentence	2,880	56.6%
**Nuclear family structure**	1,092	21.6%
**Self Help Group**	380	7.46%
**Woman is the primary decision maker for health decisions**	1,451	28.5%
**Attendance of maternity care services**		
Antenatal care 1	742	14.6%
Antenatal care 2	1,225	24.0%
Antenatal care 3	952	18.7%
Antenatal care 4+	2,176	42.7%
**Satisfied with the services provided by the ASHA**	4,699	92.2%
**Owns Phone**	3,860	75.8%
**District**		
Hoshangabad	406	7.97%
Mandsaur	821	16.1%
Rewa	2,920	57.3%
Rajgarh	948	18.6%

Survey reliability

For each survey question, inter-rater reliability was assessed by comparing reported results from face-to-face surveys conducted at two time points (test-retest; n=205), while intermodal reliability was assessed by

For peer review only - http://bmjopen.bmj.com/site/about/guidelines.xhtml

comparing findings from the face-to-face survey to phone surveys (n=375). Table 2 presents prevalence and reliability estimates by question.

Overall results suggest that population level estimates of knowledge over time and across modalities were similar, but reliability among individual women in the sample was poor. Prevalence estimates illustrate the latter; demonstrating that women's knowledge across and within domains was low. Questions with low reliability included True/false questions and those with unprompted, multiple response options.

Figure 3 graphs the correlation between the prevalence and bias adjusted kappa statistics for face-to-face surveys against the in-person re-test and the phone survey. The correlation forms a positive linear line, representing a strong positive reliability between the face-to-face and phone survey options.

For peer review only

Table 2: Prevalence and reliability estimates for knowledge questions among pregnant women

Question text	Response Options	Baseline Prevalence	Retest Prevalence	Phone Prevalence	Retest Kappa	Phone Kappa
**Domain: Maternal nutrition and pregnancy danger signs**						
What foods should you eat during pregnancy?	Fish	0.07	0.08	0.02	0.79	0.84
	Meat	0.08	0.06	0.04	0.80	0.83
	Eggs	0.13	0.08	0.05	0.79	0.68
	Milk/Dairy Products	0.73	0.79	0.59	0.32	0.17
	Fruits	0.89	0.92	0.73	-	-
	Green leafy vegetables	0.94	0.93	0.76	-	-
	Pulses and Nuts	0.86	0.88	0.34	-	-
What should you not eat or drink during your pregnancy?	Alcohol	0.03	0.02	0.01	0.94	0.93
	Coffee	0.03	0.01	0.05	0.95	0.84
	Cigarettes	0.05	0.02	0.01	0.95	0.83
	Tea	0.14	0.16	0.09	0.58	0.45
Anaemia (khoon ki kami) in pregnancy can affect the growth and development. What should you do if you are anemic?	Take IFA tablet daily	0.64	0.73	0.56	0.30	0.21
	Tea	0.40	0.63	0.07	-	-
	Cigarettes/bids	0.83	0.76	0.34	-	-
How many tetanus injections should you have during pregnancy?	2 shots	0.66	0.52	0.65	0.77	0.58
What are Iron Folic Acid tablets?	Help prevent/treat anemia	0.80	0.90	0.64	-	-
	Improve the health/well-being of my baby	0.73	0.85	0.43	-	-
What danger signs during pregnancy and before labor starts would lead you to go to the health facility immediately?	Yellowing of skin	0.10	0.02	0.01	0.88	0.78
	Vaginal Bleeding	0.31	0.32	0.10	-	-
	Vaginal Discharge	0.35	0.33	0.10	-	-
	Convulsions	0.42	0.52	0.09	-	-
	Stomach Cramps	0.87	0.95	0.45	-	-
	Swelling on limbs and face	0.38	0.30	0.20	-	-
**Domain: Infant and young child feeding**						
How many times per day should newborns be breastfed?	9-10 times a day, on demand	0.94	0.94	N/A	0.75	N/A
What are some of the benefits of breastfeeding?	It helps to maintain space between children	0.01	0.01	0.01	0.98	1.00
	Reduces expenditure on medical care as child will fall sick a fewer number of times	0.11	0.05	0.05	0.82	0.62
	The more the child breastfeeds, the more milk will be produced	0.07	0.09	0.02	0.67	0.91

For peer review only

	Promotes Mother-Baby bonding	0.09	0.01	0.27	-	-
	Help build immunity for your child	0.72	0.71	0.57	-	-
	Promotes child growth, wellness	0.85	0.96	0.54	-	-
How soon after delivery should you give foods or liquids other than mothers milk?	6 months	0.83	0.74	0.75	0.64	0.64
	Immediately	0.28	0.64	0.15	-	-
What types of foods should a baby be given after 6 months of age?	Cheese	0.05	0.07	0.01	0.73	0.85
	White potatoes, roots	0.01	0.00	0.09	0.99	0.83
	Ripe mangoes, papayas	0.05	0.10	0.05	0.71	0.82
	Beans, peas, nuts	0.14	0.08	0.02	0.75	0.52
	Bread, roti, grains	0.17	0.08	0.14	0.55	0.54
	Fruits or vegetables	0.14	0.16	0.16	0.52	0.63
	Baby food	0.42	0.39	0.47	0.05	0.07
	Plain Water	0.74	0.76	0.37	-	-
	Juice	0.10	0.07	0.35	-	-
	Lentil Broth/soup	0.18	0.14	0.73	-	-
	Milk	0.78	0.90	0.52	-	-
	Pumpkin, squash	0.78	0.88	0.38	-	-
	Solid, semi-solid, soft food	0.27	0.45	0.00	-	-
	Liver	0.00	0.00	0.00	-	-
	Chicken	0.00	0.00	0.00	-	-
	Meat	0.00	0.00	0.00	-	-
	Eggs	0.00	0.00	0.00	-	-
	Dried Fish	0.00	0.00	0.00	-	-
**Domain: Infant and young child care**						
What danger signs do you know about for the newborn after delivery?	Redness	0.01	0.01	0.01	0.98	0.99
	Red eyes	0.02	0.02	0.01	0.94	0.96
	Skin lesions	0.03	0.02	0.01	0.87	0.96
	Blueness of hands	0.03	0.01	0.01	0.96	0.91
	Convulsions	0.04	0.02	0.06	0.85	0.84
	Low birth weight	0.05	0.05	0.04	0.83	0.74
	Lethargy	0.09	0.17	0.06	0.51	0.74
	Difficulty feeding	0.22	0.17	0.16	0.50	0.28
	Yellow color of skin	0.17	0.23	0.04	0.50	0.61
	Pitched cry	0.26	0.21	0.30	0.44	0.20
	Difficulty breathing	0.36	0.35	0.05	-	-
	Baby feels hot or cold to touch	0.30	0.49	0.15	-	-
	Fever	0.83	0.93	0.34	-	-

For peer review only

	Vomiting	0.52	0.49	0.24	-	-
How soon after your baby is born should it receive its first vaccination?	At Birth	0.74	0.8	0.73	0.52	0.54
	Within 1 month	0.09	0.11	0.09	-	-
	1-2 months	0.04	0.01	0.02	-	-
	2+ months	0.02	0.01	0.02	-	-
What are things you can do to prevent your child from getting diarrhea?	Give baby safe drinking water > 6 months	0.17	0.08	0.05	0.68	0.65
	Exclusively breastfeed children < 6 months	0.14	0.06	0.05	0.69	0.63
	Cover water and food to avoid flies sitting on it	0.16	0.23	0.1	0.39	0.55
	Safe disposal of stools	0.09	0.11	0.12	0.57	0.49
	Make sure the environment is clean	0.50	0.63	0.38	0.02	-0.02
	Wash hands	0.39	0.40	0.20	-	-
What should you give your child to treat diarrhea?	Intravenous	0.01	0.01	0.01	0.96	0.98
	Antibiotic	0.03	0.00	0.01	0.96	0.95
	Injection	0.07	0.18	0.01	0.50	0.80
	Home remedy	0.10	0.02	0.01	0.93	0.78
	Salt and sugar	0.06	0.04	0.13	0.86	0.71
	ORS + Zinc	0.11	0.11	0.18	0.70	0.50
	ORS	0.40	0.48	0.27	0.38	0.24
	Antidiarrheals	0.39	0.41	0.05	-	-
	Other pill or syrup	0.62	0.69	0.38	-	-
What are three critical times for a woman to wash her hands?	After defecation	0.98	0.94	0.43	-	-
	Before cooking or handling food	0.91	0.94	0.37	-	-
	Before eating or feeding the child	0.89	0.92	0.37	-	-
**Domain: Family Planning**						
How soon after you give birth, can you get pregnant again?	Immediately	0.02	0.01	0.04	0.43	-0.02
	Not until menses return	0.73	0.8	0.12	-	-
	After you stop breastfeeding	0.03	0.01	0.14	-	-
What is the recommended length of time you should wait between having another child?	Immediately	0.00	0.00	0.00	0.61	0.36
	Wait for at least 1 year	0.02	0.02	0.02	-	-
	Wait for at least 2 years	0.19	0.27	0.18	-	-
	Wait for at least 3 years	0.67	0.64	0.51	-	-
What are the benefits of using family planning to limit the size of your family?	Easy way to control the size of your family	0.48	0.47	0.46	0.02	0.00
	Financial savings	0.50	0.51	0.50	0.01	0.06
	Give you more time to take care of the children you already have	0.70	0.82	0.54	-	-

For peer review only

Men become physically weak after accepting sterilization	FALSE	0.15	0.14	0.09	0.28	-0.02
There are many safe methods of birth control	TRUE	0.94	0.96	0.33	-	-
Female sterilization can be done at the time of birth	TRUE	0.76	0.82	0.21	-	-
Male sterilization is an easy way to control family size	TRUE	0.72	0.63	0.22	-	-
PPIUCD insertion and female sterilization	TRUE	0.87	0.86	0.35	-	-
Do you know of a place where you can obtain a method of family planning?	Yes	0.9	0.98	0.38	-	-
Which ways or methods of contraception have you heard about?	Female sterilization	0.92	0.97	0.37	-	-
	Male Sterilization	0.49	0.54	0.21	-	-
	IUD	0.47	0.36	0.31	-	-
	Oral contraceptive pills	0.84	0.93	0.58	-	-
	Injectables	0.70	0.78	0.28	-	-
	Male Condom	0.64	0.74	0.46	-	-
	Rhythm Method	0.56	0.45	0.02	-	-
	Withdrawal	0.24	0.08	0.05	-	-
**Domain: Essential newborn care**						
How do you keep the baby warm after delivery?	Put the baby on your chest	0.17	0.15	0.13	0.5	0.49
	Dried or wiped soon after birth	0.25	0.30	0.24	0.08	0.24
	Cover in clothes	0.96	1.00	0.56		
What should you put on the cord after delivery?	Nothing	0.06	0.05	0.04	0.82	0.77
	Blade used for other purposes	0.72	0.72	0.05	-	-
	Scissors	0.41	0.72	0.29	-	-
	Knife	0.02	0.03	0.18	-	-
	Surgical Blade	0.03	0.01	0.39	-	-
	New Razor blades	0.72	0.71	0.11	-	-
How soon after delivery should your baby be bathed?	1 Day	0.8	0.77	0.03	0.49	0.8

For peer review only

Determinants of RMNCH&N knowledge

Figure 4 shows average knowledge scores for the five composite knowledge domains: maternal nutrition and pregnancy danger signs (52%), family planning score (58%), immediate post-birth care for the neonate (47%), infant and young child feeding (56%), and infant and young child care (58%). The average of all domains was used to create a final composite score (54%).

Table 3 presents results from bivariate and multivariate regressions on factors influencing pregnant women's knowledge. In the multivariable model, women's knowledge was significantly higher among older women (25-34 years, p<0.001; >34 years, p=0.02); with higher education (p=0.009); able to read a full sentence (p<0.001); living in a nuclear family (p<0.001); who reported being the primary decision maker in health decisions (p<0.001); had attended a greater number of ANC visits (p<0.001); and reported being satisfied with ASHA services (p<0.001). Women from the rural districts of Mandsaur and Rajgarh had significantly lower knowledge scores than women in the reference peri-urban district of Hoshangaband. Women's phone ownership, socioeconomic status, and caste were not associated with significant differences in knowledge.

Table 3. Factors associated with pregnant women's RMNCH&N knowledge in four districts of Madhya Pradesh, India

Variable	Unadjusted Coefficient (95% Confidence Interval)	p-value	Adjusted Coefficient (95% Confidence Interval)	p-value
**Age**				
18-24	1	-	1	--
25-34	3.21(2.68-3.74)	<0.001	1.91(1.35-2.46)	<0.001
35+	1.87(-0.04-3.78)	0.055	2.04(0.34-3.74)	0.02
**Parity (>=1 child)**	4.40(3.85-4.96)	<0.001	4.39(3.74-5.03)	<0.001
**Caste**				
General	1	-	1	-
Other backwards	-0.62(-1.30-0.05)	0.070	0.62(-0.06-1.3)	0.076
Scheduled	-0.97(-1.70-0.24)	0.010	0.24(-0.58-1.06)	0.567
**Wealth Index**				
Q1	1	--	1	--
Q2	0.30(-0.52-1.13)	0.471	0.32(-0.49-1.13)	0.442
Q3	0.03(-0.80-0.86)	0.938	0.14(-0.78-1.07)	0.765
Q4	0.25(-0.58-1.08)	0.551	0.16(-0.83-1.16)	0.751
Q5	1.83(1.00-2.66)	<0.001	0.72(-0.38-1.82)	0.197
**Education level**				
No School	1	--	1	--
Primary or Less	0.71(-0.31-1.72)	0.172	0.65(-0.35-1.65)	0.201
Middle School	1.32(0.39-2.24)	0.005	0.6(-0.51-1.72)	0.289
Higher Education	3.26(2.37-4.16)	<0.001	1.65(0.42-2.89)	0.009
**Literacy**				
Cannot read at all	1	--	1	-
Reading only parts of sentence	1.43(0.52-2.34)	0.002	0.95(0.01-1.9)	0.049
Read whole sentence	3.02(2.44-3.59)	<0.001	2.29(1.42-3.15)	<0.001
**Self Help Group**	0.97(-0.03-1.97)	0.057	0.4(-0.42-1.22)	0.335
**Nuclear Family Structure**	-1.97(-2.61--1.33)_	<0.001	-2.14(-2.95--1.33)	<0.001
**Women is the primary decision maker in health decisions**	2.03(1.45-2.61)	<0.001	1.59(1-2.19)	<0.001
**Number of ANC Visits**				

1	1	--	1	--
2	1.13(0.27-2.01)	0.011	1.03(0.16-1.9)	0.021
3	1.64(0.73-2.56)	<0.001	1.95(1.04-2.85)	<0.001
4	1.14(0.34-1.94)	0.005	1.68(0.81-2.54)	<0.001
**Satisfied with the services provided by the ASHA**	2.61(2.02-3.19)	<0.001	2.18(1.57-2.79)	<0.001
**Phone Ownership**	1.31(0.71-1.93)	<0.001	0.61(-0.01-1.22)	0.055
**District**				
Hoshangabad	1	--	1	--
Mandsaur	-3.30(-4.42--2.17)	<0.001	-3.3(-4.51--2.09)	<0.001
Rewa	-1.53(-2.52--0.55)	<0.001	-0.13(-1.21-0.95)	0.814
Rajgarh	-4.65(-5.75--3.55)	<0.001	-3.05(-4.21--1.89)	<0.001

Supplementary Table 2 explores determinants of knowledge for each of the five domains. Across all domains, knowledge was significantly higher among older women (25-34 years of age), with a least one child, able to read a whole sentence, and who reported being satisfied with services provided by the ASHA. Knowledge of maternal nutrition and pregnancy danger signs was significantly higher amongst women living in a nuclear family, with higher education, and who had attended a greater number of antenatal care visits. Family planning knowledge was significantly higher amongst women in the highest socioeconomic strata, with primary school or higher education. Knowledge of immediate post-birth care for the newborns was similarly higher amongst women in the highest socioeconomic strata, and with higher education.

DISCUSSION

The study sought to develop and refine a phone survey tool for the measurement of RMNCH&N knowledge among pregnant women in four districts of Madhya Pradesh. Findings suggest that there is a correlation between face-to-face and re-test reliability and face-to-face and phone reliability. Instances were not observed where there was re-test reliability for face-to-face and poor reliability for face-to-face versus phone. Questions with unprompted and more possible response options (e.g. foods to eat during pregnancy) had similarly poor inter-rater and inter-modal reliability as questions that sought to pin respondents down to one answer (e.g. How many tetanus injections should you have during pregnancy?). This suggests that the questions themselves may not be well suited to repeat questioning. Overall knowledge levels were low for most questions asked. This underscores the need to bolster awareness among women and improve access to health information. It too may indicate that some questions and response options used were not culturally appropriate or relevant for this context (e.g. alcohol, coffee, and cigarettes are uncommonly consumed in rural India by women which may explain the low awareness of these items when asked "What should you not eat or drink during your pregnancy?"). The collective body of findings suggests that survey estimates may be appropriate for ascertaining population level estimates rapidly, however, contraindicated in instances where longitudinal tracking or individual level analyses are required. This may have critical implications for those looking to utilize remote surveys for measuring intermediate program outcomes, including knowledge.

Study findings contribute to the limited body of literature on how to optimise the development of phone surveys for use in the measurement of population level knowledge and health outcomes. Few studies to date have sought to refine phone survey tools through a rigourous process of reliability testing (23). Findings from a 2017 systematic review identified only 10 studies which have reported using reliability testing to develop phone survey tools; five of these included face-to-face and CATI survey comparisons (23). In Honduras and Peru (24), comparisons were made across independent samples, while in Lebanon (25) and two separate surveys in Brazil (26), comparisons were made using the same sample across two modalities (as in our study) (18, 22). Overall findings broadly suggest that there was concordance in the results across CATI and face-to-face modalities (21). However, for some outcomes, prevalence estimates

For peer review only

did differ across modalities (27). Studies in Honduras and Peru compared additional phone survey modalities, including text messages and interactive voice response (IVR), and concluded that CATI surveys had the lowest discordance with the face-to-face surveys (24). Elsewhere, more recent efforts have sought to compare the reliability of alternative phone survey delivery modalities (IVR versus CATI) for measuring non-communicable disease risk factors (28). In our context, low literacy rates precluded testing phone survey modalities other than CATI surveys.

Underpinning efforts to rigorously develop the phone survey tool was a design to measure knowledge among pregnant women. Overall knowledge levels among pregnant women in these four districts of Madhya Pradesh ranged from 47% for essential newborn care to 58% for family planning. Areas of lower knowledge included what should be put on the cord after delivery, how to keep the baby warm after delivery, what foods to eat during pregnancy, what food and drinks to avoid during pregnancy, etc. Knowledge scores were significantly higher amongst older women; those with higher levels of education and literacy; living in a nuclear family; who reported being the primary decision maker in health decisions; who had attended a greater number of ANC visits; and reported being satisfied with ASHA services.

Other studies have measured women's knowledge levels on topics such as neonatal danger signs, immediate post-birth care for the neonate, infant and young child feeding, breastfeeding, and family planning. Compared to 18% of women in Ethiopia and 15% of women in Uganda who have good knowledge of neonatal danger signs, our women have a higher relative knowledge at 58% (29,30). In a survey among hospital-delivered mothers in Sri Lanka, while 90% of mothers knew about advantages of breastfeeding compared to our survey of 56%, and only 22% correctly answered what should be applied to an umbilical cord compared to our survey at 47% (31). In India, universal knowledge existed among married women in Uttar Pradesh about at least one family planning method and 90% of women in Lucknow were aware of contraceptive methods in family planning existed comparatively to our study's lower family planning knowledge at 58% (32,33). This is all subject to each study's own verified survey methods and composite score generation. Developing one standardized survey questionnaire across studies to measure women's RMNCH&N knowledge would lend more insight into how comparative this data is.

There is limited empirical evidence of factors influencing pregnant women's knowledge. A study conducted in Delhi assessed knowledge components that included immunization against tetanus, number of antenatal visits, folic acid tablets, danger signs during pregnancy or labor, and discussions with elders, mothers-in-law, doctors, or friends (34). Like our study, it found that women in joint families were also significantly more likely to have higher knowledge (34). Another study in Rewa district also found that women whose husbands and in-laws played a dominate role in decision making had lower knowledge of key danger signs during pregnancy (35). This is consistent with our study, which included Rewa district among our four study districts, as we found if the women played a role in the decision-making process, she had significantly higher knowledge of maternal danger signs. In a Uganda study, no association was found with attending ANC visits and knowledge of danger signs, which is inconsistent with our findings (30). Surprisingly, while NFHS found that more family planning methods were seen in women of higher wealth index, wealth was not found to be a determinant in this study (11). Taken together, our findings suggest to increase women's knowledge in the future, one should empower women to play a larger role in the health decision making process, strengthen ASHA services, promote education, and increase access and uptake of ANC services. Further research to understand sources and quality of sources for knowledge should be undertaken as well.

Limitations

This survey was limited to RMNCH&N knowledge in women 5-7 months pregnant with access to a mobile phone during the day, in four predominately rural districts of Madhya Pradesh. This may have led to a pro-rich bias in the sampling given the profile of beneficiaries with access to mobile phones (36). Survey questions were derived from expert review and large national survey tools, including the demographic and health survey for India and the Multiple Indicator Cluster Survey (MICS). Cognitive interviews were used

For peer review only

to refine questions on respectful maternity care; a process described elsewhere (19, 20, 37). Ideally the same process would have been followed for refining knowledge questions. However, budget and time constraints made this added step infeasible. Phone surveys were administered by Delhi based enumerators whose demographic profile differed from respondents. Alternative phone survey modalities, including text messages, interactive voice response, and USSD were considered but deemed less desirable to using computer assisted telephone interviews (CATI) surveys which could allow beneficiaries to speak with and ask for clarification questions.

CONCLUSIONS

Study findings offer insight into the challenges associated with reliably measuring RMNCH&N knowledge among pregnant women using face-to-face and remote survey tools. Population level estimates of knowledge over time and across modalities were similar, but reliability among individual women in our sample was poor. This suggests that the use of phone surveys to measure knowledge is contraindicated in instances where longitudinal tracking or individual level analyses are required however it may be appropriate as a means of gaining population level insights. Among women assessed, overall knowledge levels were low; however, knowledge scores were significantly higher amongst older women; those with higher levels of education and literacy; living in a nuclear family; who reported being the primary decision maker in health decisions; who had attended a greater number of ANC visits; and reported being satisfied with ASHA services.

ACKNOWLEDGEMENTS

We thank the women and families of Madhya Pradesh who generously gave of their time to support this work. We are humbled by the opportunity to convey their perspectives and experiences. We additionally are grateful to Neha Dumke and the team at the National Health Systems Resource Centre for their support. This work was made possible by the Bill and Melinda Gates Foundation. We thank Diva Dhar, Suhel Bidani, Rahul Mullick, Dr. Suneeta Krishnan, Dr. Neeta Goel and Dr. Priya Nanda for believing in us and giving us this opportunity. We additionally wish to thank are grateful to the larger team of enumerators from OPM-India and NHSRC who worked tirelessly over many months to implement the surveys that form the backbone of our analyses. We additionally thank Vinit Pattnaik at OPM and Erica Crawford at Johns Hopkins University for their support to the financial management support of our work.

REFERENCES

1. McDougal L, Raj A, Yore J, et al. Strengthening Gender Measures and Data in the COVID-19 Era : An Urgent Need for Change (English*)*. 2021. World Bank Group. http://documents.worldbank.org/curated/en/681671619424596227/Strengthening-Gender-Measures-and-Data-in-the-COVID-19-Era-An-Urgent-Need-for-Change
2. Menon S, Sonderegger P, Totapally S. Five questions to consider when conducting COVID-19 phone research. BMJ Global Health 2021;6 :e004917. doi:10.1136/ bmjgh-2020-004917
3. Himelein K, Eckman S, Lau C, et al. Mobile phone surveys for understanding COVID-19 impacts: Part I sampling and Mode [Internet]. World Bank Blogs. Development Impact; [cited 2021Nov13]. Available from: https://blogs.worldbank.org/impactevaluations/mobile-phone-surveys-understanding-covid-19-impacts-part-i-sampling-and-mode
4. Gibson DG, Pereira A, Farrenkopf BA, et al. Mobile phone surveys for collecting population-level estimates in low- and middle-income countries: A literature review. Journal of Medical Internet Research. 2017;19(5).
5. Kopper S, Sautmann A. Best practices for conducting phone surveys [Internet]. The Abdul Latif Jameel Poverty Action Lab (J-PAL). 2020 [cited 2021Nov13]. Available from: https://www.povertyactionlab.org/blog/3-20-20/best-practices-conducting-phone-surveys
6. Labrique A, Blynn E, Ahmed S, et al. Health surveys using mobile phones in developing countries: Automated Active Strata Monitoring and other statistical considerations for improving precision and reducing biases. Journal of Medical Internet Research. 2017;19(5).

7. Khalil K, Das P, Kammowanee R, et al. Ethical considerations of phone-based interviews from three studies of covid-19 impact in Bihar, India. BMJ Global Health. 2021;6(Suppl 5).
8. Ali J, Labrique AB, Gionfriddo K, et al. Ethics considerations in Global Mobile Phone-based surveys of Noncommunicable Diseases: A conceptual exploration. Journal of Medical Internet Research. 2017;19(5).
9. Gibson DG, Pariyo GW, Wosu AC, et al. Evaluation of mechanisms to improve performance of mobile phone surveys in low- and middle-income countries: Research protocol. JMIR Research Protocols. 2017;6(5).
10. Nair H, Williams LJ, Marsh A, et al. Assessing the reactivity to mobile phones and repeated surveys on reported care-seeking for common childhood illnesses in rural India. Journal of Global Health. 2018;8(2).
11. International Institute for Population Sciences. NATIONAL FAMILY HEALTH SURVEY (NFHS-4) 2015-16 INDIA [Internet]. Deonar, Mumbai; 2017 [cited 2019 Mar 3]. Available from: http://www.rchiips.org/nfhs
12. Manesh AO, Sheldon TA, Pickett KE, et al. Accuracy of child morbidity data in demographic and health surveys. International Journal of Epidemiology. 2007;37(1):194–200.
13. Mohan D, Bashingwa JJ, Tiffin N, et al. Does having a mobile phone matter? Linking phone access among women to health in India: An exploratory analysis of the National Family Health Survey. PLOS ONE. 2020;15(7).
14. International Telecommunication Union. The World in 2016: ICT Facts and Figures [Internet]. [cited 2019 Mar 9]. Available from: https://www.itu.int/en/ITU-D/Statistics/Pages/stat/default.aspx
15. Okonjo-Iweala N, Osafo-Kwaako P. Improving health statistics in Africa. The Lancet. 2007;370(9598):1527–8.
16. Raju S, Ayyar Panchapagesan N. Madhya Pradesh [Internet]. Encyclopædia Britannica, inc. [cited 2019 Mar 4]. Available from: https://www.britannica.com/place/Madhya-Pradesh
17. National Family Health Survey. State Fact Sheet Madhya Pradesh [Internet]. 2016 [cited 2019 Mar 3]. Available from: http://rchiips.org/nfhs/pdf/NFHS4/MP_FactSheet.pdf
18. LeFevre A, Agarwal S, Chamberlain S, et al. Are stage-based health information messages effective and good value for money in improving maternal newborn and child health outcomes in India? Protocol for an individually randomized controlled trial. Trials. 2019;20(1).
19. LeFevre AE, Scott K, Mohan D, et al. Development of a Phone Survey Tool to Measure Respectful Maternity Care During Pregnancy and Childbirth in India: Study Protocol. JMIR Research Protocols. 2019;8(4)
20. Scott K, Gharai D, Sharma M, et al. Yes, no, maybe so: the importance of cognitive interviewing to enhance structured surveys on respectful maternity care in northern India. Health Policy and Planning. 2019
21. Shah N, Mohan D, Agarwal S, et al. Novel approaches to measuring knowledge among frontline health workers in India: Are phone surveys a reliable option? PLOS ONE. 2020;15(6).
22. Terwee CB, Bot SD, de Boer MR, et al. Quality criteria were proposed for measurement properties of health status questionnaires. J Clin Epidemiol. 2007 Jan; 60(1):34-42.
23. Greenleaf AR, Gibson DG, Khattar C, et al. Building the Evidence Base for Remote Data Collection in Low- and Middle-Income Countries: Comparing Reliability and Accuracy Across Survey Modalities. Journal of Medical Internet Research. 2017;19(5).
24. Ballivian A, Azevedo J, Durbin W, et al. Using mobile phones for high-frequency data collection. In: Toninelli D, Pinter R, de Pedraza P, editors. Mobile Research Methods: Opportunities and Challenges of Mobile Research Methodologies. London: Ubiquity Press; 2015:21-39.
25. Mahfoud Z, Ghandour L, Ghandour B, et al. Cell phone and face-to-face interview responses in population-based surveys: how do they compare? Field Methods 2014 Jul 01;27(1):39-54.
26. Ferreira A, César CC, Malta DC, et al. Validade de estimativas obtidas por inquérito telefônico: comparação entre VIGITEL 2008 e inquérito Saúde em Beagá. Rev bras epidemiol 2011 Sep;14(1):16-30.
27. Francisco P, Azevedo Barros MB, Segri N, et al. Comparação de estimativas para o auto-relato de condições crônicas entre inquérito domiciliar e telefônico - Campinas (SP), Brasil. Rev bras epidemiol 2011 Sep;14(3):5-15.
28. Pariyo GW, Greenleaf AR, Gibson DG, et al. Does mobile phone survey method matter? Reliability of computer-assisted telephone interviews and interactive voice response non-communicable diseases risk factor surveys in low and middle income countries. PLOS ONE. 2019;14(4).
29. Nigatu SG, Worku AG, Dadi AF. Level of mother ' s knowledge about neonatal danger signs and associated factors in North West of Ethiopia : a community based study. BMC Res Notes. 2015;4–9.
30. Sandberg J, Odberg Pettersson K, Asp G, et al. Inadequate Knowledge of Neonatal Danger Signs among Recently Delivered Women in Southwestern Rural Uganda: A Community Survey. PLoS One. 2014 May 13;9(5):e97253.
31. Senarath U, Fernando D, Vimpani G, et al. Factors associated with maternal knowledge of newborn care

For peer review only

among hospital-delivered mothers in Sri Lanka. Transactions of the Royal Society of Tropical Medicine and Hygiene. 2007;101(8):823–30.

32. Singh A, Singh KK, Verma P. Knowledge, attitude and practice GAP in family planning usage: an analysis of selected cities of Uttar Pradesh. Contraception and Reproductive Medicine. 2016;1(1).

33. Sharma V, Mohan U, Das V, et al. Socio demographic determinants and knowledge, attitude, practice: Survey of family planning. Journal of Family Medicine and Primary Care. 2012;1(1):43.

34. Acharya AS, Kaur R, Prasuna JG, et al. Making Pregnancy Safer-Birth Preparedness and Complication Readiness Study Among Antenatal Women Attendees of A Primary Health Center, Delhi. Indian Journal of Community Medicine. 2015;40(2):127.

35. Deoki N, Kushwah SS, Dubey DK, et al. A study for assessing birth preparedness and complication readiness intervention in Rewa District of Madhya Pradesh Chief Investigator, India. Department of Community Medicine, SS Medical College, Rewa, MP. 2008;9.

36. Mohan D, Bashingwa JJ, Tiffin N, et al. Does having a mobile phone matter? Linking phone access among women to health in India: An exploratory analysis of the National Family Health Survey. PLOS ONE. 2020;15(7).

37. Scott K, Ummer O, LeFevre AE. The devil is in the detail: reflections on the value and application of cognitive interviewing to strengthen quantitative surveys in global health. Health Policy and Planning. 2021;36(6):982–95.

For peer review only

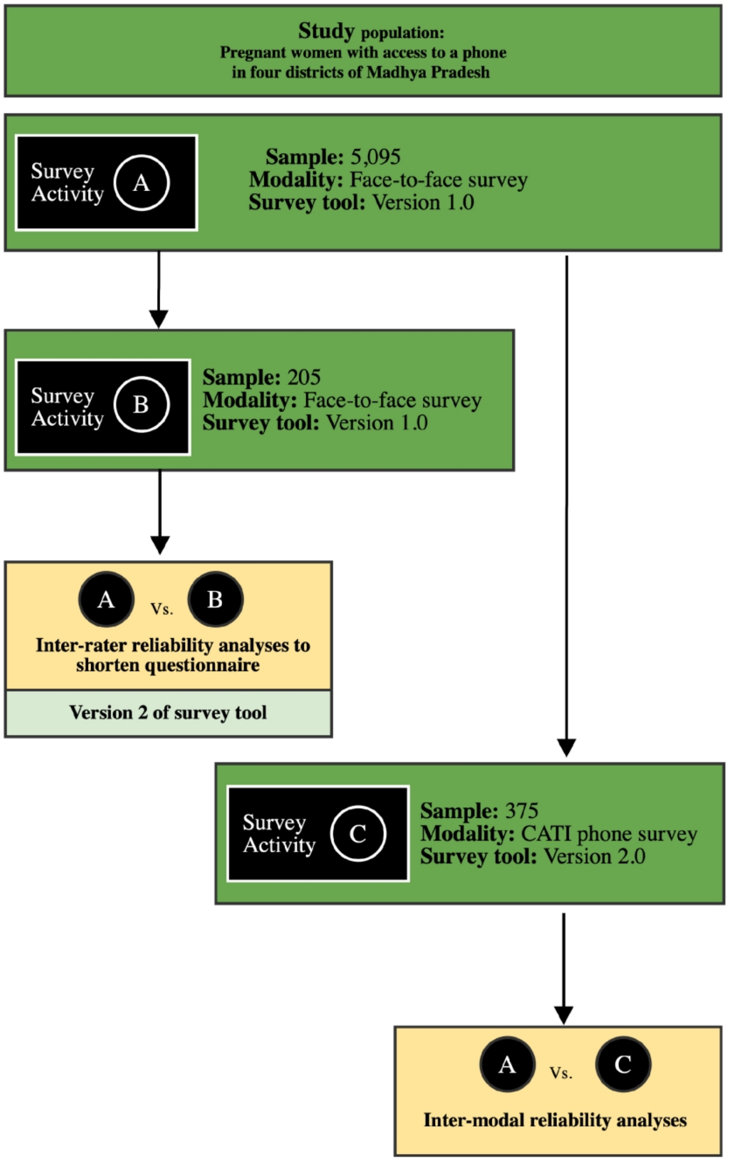

Figure 1. Completed interviews for face-to-face and CATI surveys

177x273mm (300 x 300 DPI)

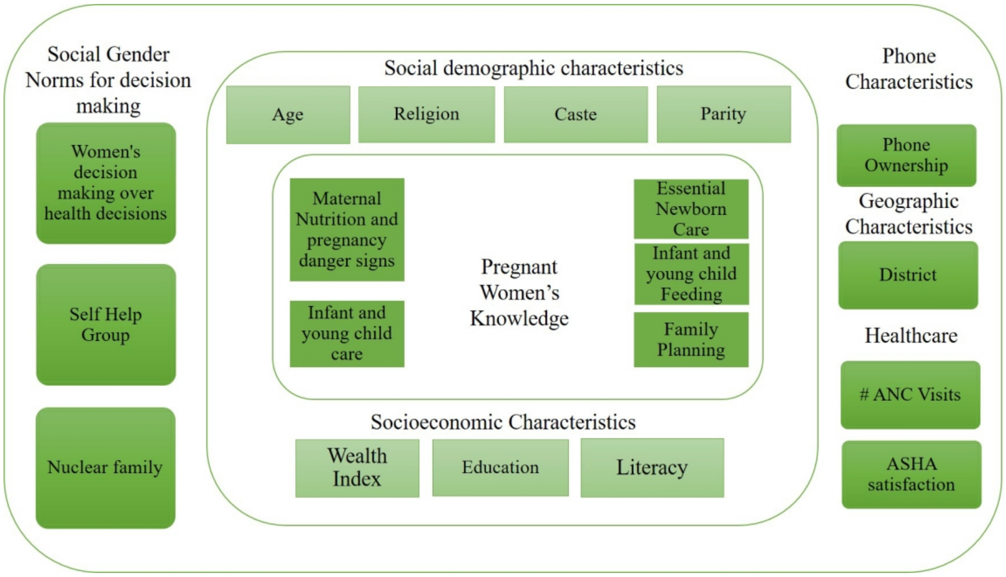

Figure 2. Conceptual framework for identifying the determinants of pregnant women's RMNCH&N knowledge

670x381mm (300 x 300 DPI)

For peer review only - http://bmjopen.bmj.com/site/about/guidelines.xhtml

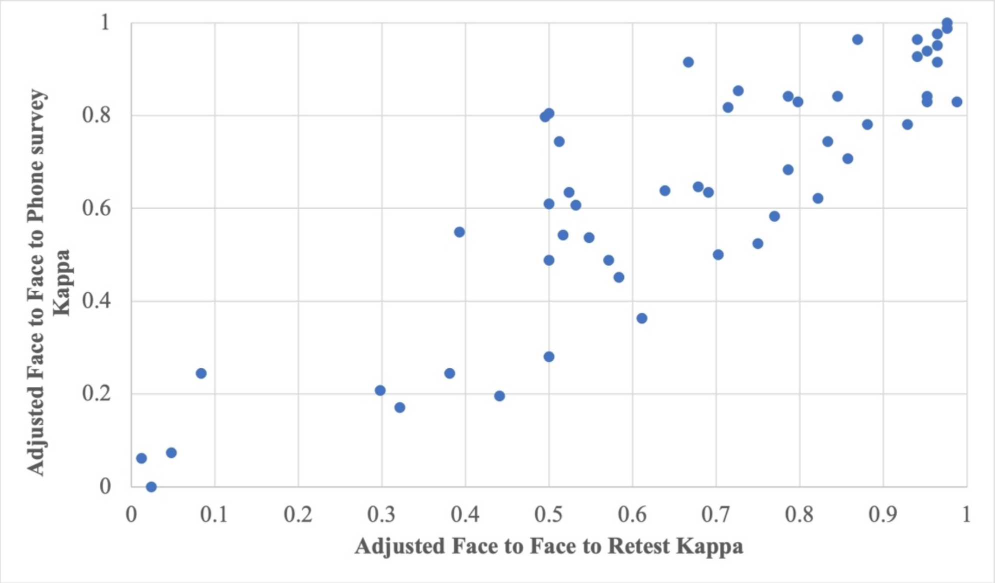

Figure 3. Measurement of degree in which repeated measurements in pregnant women interviewed (test-retest) provide similar answers

180x105mm (300 x 300 DPI)

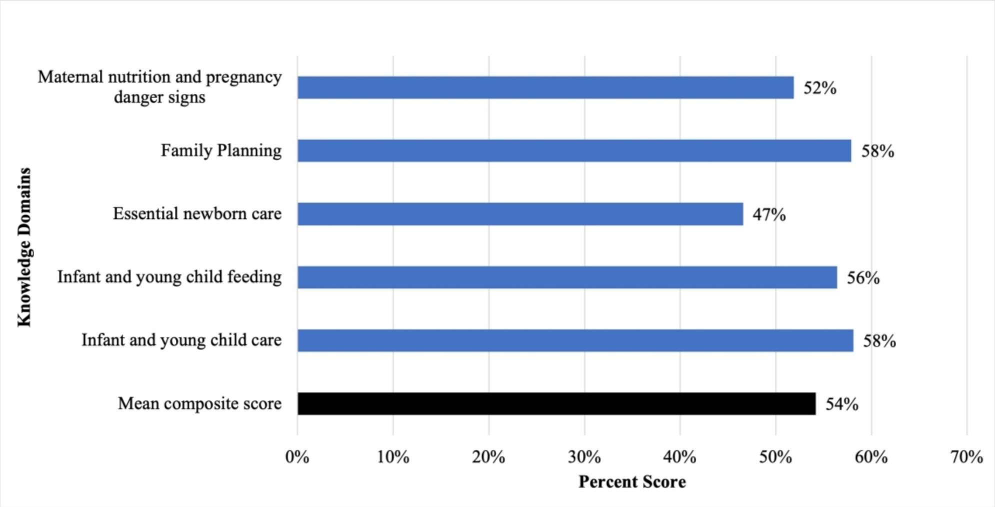

Figure 4. Average knowledge scores for composite knowledge domains

212x109mm (300 x 300 DPI)

Supplementary Table 1: Questions included in each domain of knowledge

Questions	Answer options	Correct answer(s)
**Maternal nutrition and pregnancy danger signs (6 questions)**		Maximum possible score: 6
What foods should you eat during pregnancy?	Unprompted free list, enumerator noted any correct answers mentioned	Milk/dairy products; fruits; green leafy vegetables; pulses and nuts; egg; meat; fish
What should you not eat or drink during your pregnancy?	Unprompted free list, enumerator noted any correct answers mentioned	Tea; coffee; cigarettes/bidis; alcohol
What should you do if you are anemic?	Unprompted free list, enumerator noted any correct answers mentioned	Have your blood checked regularly; take IFA tablets daily; eat well
What are the benefits of taking Iron Folic Acid tablets during pregnancy?	Unprompted free list, enumerator noted any correct answers mentioned	Help prevent/treat anemia; improve the health/well-being of my baby
How many tetanus injections should you have during pregnancy?	Number or don't know	2
What danger signs during pregnancy and before labor starts would lead you to go to the health facility immediately?	Unprompted free list, enumerator noted any correct answers mentioned	Vaginal bleeding; vaginal discharge; convulsions; stomach cramps; swelling on limbs and face; yellowing of skin; fever; decreased or absent fetal movements
**Immediate post-birth care for the neonate (4 questions)**		Maximum possible score: 4
How soon after delivery should your baby be bathed?		
How should you keep the baby warm after delivery?	Unprompted free list, enumerator noted any correct answers mentioned	
What should be used to cut the umbilical cord?	Unprompted free list, enumerator noted any correct answers mentioned	
What should be put on the cord after delivery?	Unprompted free list, enumerator noted any correct answers mentioned	
**Infant and young child feeding (5 questions)**		Maximum possible score: 5
What are some of the benefits of breastfeeding?	Unprompted free list, enumerator noted any correct answers mentioned	Promotes mother-baby bonding; helps build immunity for child; promotes child growth, wellness;<br>Reduces expenditure on medical care as child will fall sick less; the more the child breastfeeds, the more milk will be produced; it helps to maintain space between two children
How soon after delivery should you breastfeed your child?	Enumerator noted "immediately", number of hours or days mentioned, or "don't know"	Immediately or <1 hour

For peer review only

Questions	Answer options	Correct answer(s)
How many times per day should newborn babies be breastfed?	Enumerator can note if respondent provides either of the correct answers or "don't know"	9-10 times in 24 hours; On-demand (as often as the baby wants to feed)
How soon after delivery should you give foods or liquids other than mother's milk?		Six months
What types of foods should a baby be given after 6 months of age?	Unprompted free list, enumerator noted any correct answers mentioned	
**Infant and young child care (5 questions)**		Maximum possible score: 5
What danger signs do you know about for the newborn after delivery that would cause you to take the baby to the health facility immediately?	Unprompted free list, enumerator noted any correct answers mentioned	
How soon after your baby is born should it receive its first vaccination?		
What should you give your child to treat diarrhea?	Unprompted free list, enumerator noted any correct answers mentioned	
What are things you can do to prevent your child from getting diarrhea?	Unprompted free list, enumerator noted any correct answers mentioned	
What are three critical times for a woman to wash her hands?	Unprompted free list, enumerator noted any correct answers mentioned	
**Family planning (9 questions)**		Maximum possible score: 9
How soon after you give birth can you get pregnant again?		
What is the recommended length of time you should wait between having this child and getting pregnant with another child?		
What are benefits of using family planning?	Unprompted free list, enumerator noted any correct answers mentioned	
Which ways or methods contraception have you heard about?	Unprompted free list, enumerator noted any correct answers mentioned	
True/False: There are many safe methods of birth control	True / false / don't know	True
True/False: Female sterilization can be done at the time of giving birth	True / false / don't know	True
True/False: Male sterilization is an easy way to control family size	True / false / don't know	True
True/False: Men become physically weak after accepting male sterilization	True / false / don't know	False
True/False: PPIUCD insertion and female sterilization services can be free of cost at government facilities	True / false / don't know	True

For peer review only

Supplementary Table 2: Factors associated with pregnant women's RMNCH&N knowledge by domain in four districts of Madhya Pradesh, India

Variable	Mean composite score	Maternal nutrition and pregnancy danger signs	Family Planning	Infant and young child care	Immediate post-birth care for newborns	Infant and young child feeding
**Age**						
18-24	Reference					
25-34	1.91(1.35-2.46)*	1.95(1.02-2.87)*	1.92(1.18-2.67)*	1.85(1.11-2.58)*	2.32(1.1-3.54)*	1.51(0.55-2.47)*
35+	2.04(0.34-3.74)*	2.19(-0.52-4.9)	4.67(2.06-7.29)*	0.81(-1.33-2.95)	1.53(-2.42-5.47)	1.01(-2.36-4.38)
**Parity (>=1 child)**	4.39(3.74-5.03)*	3.29(2.33-4.26)*	1.74(0.92-2.56)*	5.93(5.13-6.74)*	6.8(5.46-8.14)*	4.18(3.11-5.25)*
**Caste**						
General	Reference					
Other backwards	0.62(-0.06-1.3)	0.51(-0.63-1.65)	0.07(-0.83-0.96)	0.82(-0.04-1.68)	1.94(0.47-3.41)*	-0.25(-1.41-0.91)
Scheduled	0.24(-0.58-1.06)	0.12(-1.2-1.43)	0.35(-0.71-1.4)	-0.04(-1.05-0.96)	1.08(-0.63-2.79)	-0.31(-1.65-1.04)
**Wealth Index**						
Q1	Reference					
Q2	0.32(-0.49-1.13)	-0.61(-1.99-0.78)	1.33(0.23-2.43)*	0.67(-0.4-1.75)	0.66(-1.01-2.33)	-0.47(-1.85-0.9)
Q3	0.14(-0.78-1.07)	0.17(-1.37-1.71)	1.17(-0.04-2.37)	0.5(-0.69-1.7)	-1.24(-3.06-0.57)	0.11(-1.44-1.66)
Q4	0.16(-0.83-1.16)	-0.02(-1.65-1.61)	0.95(-0.33-2.24)	0.85(-0.41-2.1)	-2.32(-4.28--0.36)*	1.35(-0.3-2.99)
Q5	0.72(-0.38-1.82)	1.8(-0.01-3.6)	2.32(0.93-3.72)*	1.21(-0.19-2.62)	-2.95(-5.22--0.68)*	1.24(-0.55-3.03)
**Education**						
No School	Reference					
Primary or Less	0.65(-0.35-1.65)	1.87(0.24-3.5)*	1.57(0.2-2.95)*	0.01(-1.34-1.36)	-1.55(-3.6-0.5)	1.35(-0.43-3.13)
Middle School	0.6(-0.51-1.72)	1.19(-0.56-2.93)	2.43(0.92-3.94)*	0.14(-1.27-1.54)	-1.78(-4.06-0.51)	1.04(-0.83-2.91)
Higher Education	1.65(0.42-2.89)*	3.42(1.51-5.34)*	3.29(1.63-4.94)*	1.5(-0.03-3.03)	-2.82(-5.41--0.24)*	2.87(0.83-4.91)*
**Literacy**						
Cannot read at all	Reference					
Reading only parts of sentence	0.95(0.01-1.9)*	0.93(-0.57-2.42)	0.88(-0.42-2.18)	0.91(-0.33-2.15)	0.33(-1.64-2.31)	1.7(0.05-3.34)*
Read whole sentence	2.29(1.42-3.15)*	3.12(1.78-4.46)*	2.29(1.17-3.41)*	1.4(0.33-2.47)*	1.86(0.11-3.61)*	2.76(1.32-4.19)*
**Self Help Group**	0.4(-0.42-1.22)	0.88(-0.57-2.34)	0.77(-0.46-2.01)	0.45(-0.71-1.61)	-0.18(-2.33-1.98)	0.08(-1.39-1.54)
**Nuclear Family**	-2.14(-2.95--1.33)*	-2.61(-3.83--1.38)*	-1.7(-2.65--0.75)*	-4.38(-5.39--3.38)*	-1.46(-2.92-0)	-0.57(-1.81-0.67)

For peer review only

**Primary decision maker in health decisions**	1.59(1-2.19)*	2.74(1.75-3.74)*	0.48(-0.32-1.27)	2.61(1.88-3.34)*	0.43(-0.8-1.66)	1.71(0.68-2.74)*
**Number of ANC Visits**						
1	Reference					
2	1.03(0.16-1.9)*	4.19(2.8-5.59)*	0.62(-0.62-1.86)	-0.03(-1.15-1.08)	0.18(-1.59-1.95)	0.17(-1.24-1.58)
3	1.95(1.04-2.85)*	5.91(4.37-7.45)*	0.35(-0.94-1.63)	0.65(-0.51-1.81)	1.38(-0.49-3.25)	1.44(-0.05-2.93)
4	1.68(0.81-2.54)*	6.61(5.13-8.1)*	0.19(-1.01-1.4)	0.43(-0.67-1.54)	0.12(-1.67-1.9)	1.02(-0.42-2.46)
**Satisfied with the services provided by the ASHA**	2.18(1.57-2.79)*	2.78(1.83-3.72)*	2.06(1.24-2.87)*	1.7(0.9-2.5)*	2.93(1.72-4.13)*	1.44(0.42-2.46)*
**Phone Ownership**	0.61(-0.01-1.22)	0.82(-0.2-1.85)	0.71(-0.11-1.52)	0.73(-0.1-1.57)	0.4(-0.82-1.63)	0.37(-0.67-1.4)
**District**						
Hoshangabad	Reference					
Mandsaur	-3.3(-4.51--2.09)*	-1.72(-3.54-0.1)	-1.49(-2.98-0)	-2.14(-3.59--0.7)*	-6.98(-9.75--4.21)*	-4.18(-5.98--2.38)*
Rewa	-0.13(-1.21-0.95)	-3.05(-4.78--1.31)*	-0.85(-2.24-0.54)	-0.69(-2.02-0.63)	3.58(1.18-5.98)*	0.36(-1.31-2.03)
Rajgarh	-3.05(-4.21--1.89)*	-6.59(-8.41--4.78)*	-1.26(-2.76-0.24)	-0.26(-1.68-1.16)	-4.35(-6.92--1.78)*	-2.77(-4.53--1.01)*

*= Significant at 0.05 level

For peer review only

BMJ Open

Assessing the reliability of phone surveys to measure reproductive, maternal, and child health knowledge among pregnant women in rural India: a feasibility study

Journal:	*BMJ Open*
Manuscript ID	bmjopen-2021-056076.R2
Article Type:	Original research
Date Submitted by the Author:	14-Feb-2022

Complete List of Authors:	Ng, Angela; Johns Hopkins University Bloomberg School of Public Health<br>Mohan, Diwakar; Johns Hopkins University Bloomberg School of Public Health<br>Shah, Neha; Johns Hopkins University Bloomberg School of Public Health<br>Scott, Kerry; Johns Hopkins University Bloomberg School of Public Health<br>Ummer, Osama; Oxford Policy Management; BBC Media Action,<br>Chamberlain, Sara; BBC Media Action, BBC Media Action, India; BBC Media Action, Asia<br>Bhatnagar, Aarushi; World Bank New Delhi Office, Health, Nutrition and Population<br>Dhar, Diva; Bill & Melinda Gates Foundation<br>Agarwal , Smisha ; Johns Hopkins University Bloomberg School of Public Health, Department of International Health<br>Ved, Rajani; National Health Systems Resource Centre<br>LeFevre, Amnesty; Johns Hopkins University Bloomberg School of Public Health, International Health

**Primary Subject Heading**:	Global health
Secondary Subject Heading:	General practice / Family practice, Paediatrics
Keywords:	Community child health < PAEDIATRICS, Maternal medicine < OBSTETRICS, Neonatal intensive & critical care < INTENSIVE & CRITICAL CARE

SCHOLARONE™ Manuscripts

I, the Submitting Author has the right to grant and does grant on behalf of all authors of the Work (as defined in the below author licence), an exclusive licence and/or a non-exclusive licence for contributions from authors who are: i) UK Crown employees; ii) where BMJ has agreed a CC-BY licence shall apply, and/or iii) in accordance with the terms applicable for US Federal Government officers or employees acting as part of their official duties; on a worldwide, perpetual, irrevocable, royalty-free basis to BMJ Publishing Group Ltd ("BMJ") its licensees and where the relevant Journal is co-owned by BMJ to the co-owners of the Journal, to publish the Work in this journal and any other BMJ products and to exploit all rights, as set out in our licence.

The Submitting Author accepts and understands that any supply made under these terms is made by BMJ to the Submitting Author unless you are acting as an employee on behalf of your employer or a postgraduate student of an affiliated institution which is paying any applicable article publishing charge ("APC") for Open Access articles. Where the Submitting Author wishes to make the Work available on an Open Access basis (and intends to pay the relevant APC), the terms of reuse of such Open Access shall be governed by a Creative Commons licence – details of these licences and which Creative Commons licence will apply to this Work are set out in our licence referred to above.

Other than as permitted in any relevant BMJ Author's Self Archiving Policies, I confirm this Work has not been accepted for publication elsewhere, is not being considered for publication elsewhere and does not duplicate material already published. I confirm all authors consent to publication of this Work and authorise the granting of this licence.

For peer review only

Title	Assessing the reliability of phone surveys to measure reproductive, maternal, and child health knowledge among pregnant women in rural India: a feasibility study
**Author's Names**	Angela Ng, Diwakar Mohan (0000-0002-7532-366X), Neha Shah, Kerry Scott (0000-0003-3597-9637), Osama Ummer (0000-0002-4189-5328), Sara. Chamberlain (0000-0003-4785-6482), Aarushi Bhatnagar, Diva Dhar, Smisha Agarwal, Rajani Ved, Amnesty E. LeFevre (0000-0001-8437-7240), on behalf of the Kilkari Impact Evaluation Team*
**Address for each author**	**Angela Ng MSPH**<br>Department of International Health, Johns Hopkins Bloomberg School of Public Health, 615 N. Wolfe St, Baltimore, Maryland, USA<br>angelang1201@gmail.com<br><br>**Diwakar Mohan DrPH MD**<br>Title: Assistant Scientist<br>Department of International Health, Johns Hopkins Bloomberg School of Public Health, 615 N. Wolfe St, Baltimore, Maryland, USA<br>dmohan3@jhu.edu<br>ORCiD iD: 0000-0002-7532-366X<br><br>**Neha Shah MSPH**<br>Title: Research Associate<br>Department of International Health, Johns Hopkins Bloomberg School of Public Health, 615 N. Wolfe St, Baltimore, Maryland, USA<br>nshah67@jhu.edu<br><br>**Kerry Scott PhD, MSc**<br>Title: Associate<br>Department of International Health, Johns Hopkins Bloomberg School of Public Health, 615 N. Wolfe St, Baltimore, Maryland, USA<br>kscott26@jhu.edu<br>ORCiD iD: 0000-0003-3597-9637<br><br>**Osama Ummer MHA, BAMS**<br>Title: Research Manager<br>(1) BBC Media Action, India<br>Innov8 Old Fort Saket District Mall, Saket District Centre, Sector 6, Pushp Vihar, New Delhi, Delhi 110017, India<br>(2) Oxford Policy Management-Delhi, 4/6 First Floor, Siri Fort Institutional Area, New Delhi, Delhi 110049, India<br>kposamaummer@gmail.com<br>ORCiD iD: 0000-0002-4189-5328<br><br>**Sara Chamberlain, MA**<br>Title: Digital Director, BBC Media Action, India<br>Innov8 Old Fort Saket District Mall, Saket District Centre, Sector 6, Pushp Vihar, New Delhi, Delhi 110017, India<br>sara.chamberlain@in.bbcmediaaction.org<br>ORCiD iD: 0000-0003-4785-6482<br><br>**Aarushi Bhatnagar**<br>Title: Health Economist<br>Oxford Policy Management-Delhi, 4/6 First Floor, Siri Fort Institutional Area, New Delhi, Delhi 110049, India<br>aarushibhatnagar@gmail.com<br><br>**Diva Dhar**

For peer review only

	Title: Senior Program Officer, Gender Data & Evidence<br>Bill and Melinda Gates Foundation, 500 5th Ave N, Seattle, WA 98109 USA<br>Diva.Dhar@gatesfoundation.org<br><br>**Smisha Agarwal**<br>Title: Assistant Professor<br>Department of International Health, Johns Hopkins Bloomberg School of Public Health, 615 N. Wolfe St, Baltimore, Maryland, USA<br>sagarw23@jhu.edu<br><br>**Rajani Ved**<br>Title: Executive Director<br>National Health Systems Resource Centre, Baba Gangnath Marg, Munirka, New Delhi 110067<br>rajani.ved@gmail.com<br><br>**Amnesty LeFevre PhD, MHS**<br>Title: Associate Professor<br>1. School of Public Health and Family Medicine, University of Cape Town, Cape Town, South Africa<br>2. Department of International Health, Johns Hopkins Bloomberg School of Public Health, 615 N. Wolfe St, Baltimore, Maryland, USA<br>Email: aelefevre@gmail.com<br>ORCiD iD: 0000-0001-8437-7240<br><br>On behalf of the Kilkari Impact Evaluation Team<br>*(listed in Alphabetical order) Smisha Agarwal, Salil Arora, Jean JH Bashingwa, Aarushi Bhatanagar, Sara Chamberlain, Rakesh Chandra, Arpita Chakraborty, Neha Dumke, Priyanka Dutt, Anna Godfrey, Suresh Gopalakrishnan, Nayan Kumar, Simone Honikman, Alain Labrique, Amnesty LeFevre, Jai Mendiratta, Molly Miller, Radharani Mitra, Diwakar Mohan, Deshen Moodley, Nicola Mulder, Angela Ng, Dilip Parida, Nehru Penugonda, Sai Rahul, Shiv Rajput, Neha Shah, Kerry Scott, Aashaka Shinde, Aaditya Singh, Nicki Tiffin, Osama Ummer, Rajani Ved, Falyn Weiss, Sonia Whitehead
**Contributions**	AEL is the overall study PI and conceived the idea for this study along with DM, AB, RV, KS, and SC. Data collection tools were designed by AL, NS, DM, KS, AB, SA, OU, and RV with inputs from DD. Phone survey data collection was led and supervised by NS and RV. Face-to-face data collection was supervised by AB, OU, AEL, DM and AL. AN and NS led the analyses with oversight from DM and inputs from AL. AN and AL wrote the manuscripts with edits and inputs from all co-authors. AN generated all final tables and figures.
**Keywords**	Phone Surveys, Knowledge, Pregnant Women, India, Madhya Pradesh
**Corresponding Author**	**Angela Ng, MSPH**<br>Department of International Health, Johns Hopkins Bloomberg School of Public Health, 615 N. Wolfe St, Baltimore, Maryland, USA<br>angelang1201@gmail.com

Copyright: The Corresponding Author has the right to grant on behalf of all authors and does grant on behalf of all authors, an exclusive licence on a worldwide basis to the BMJ Publishing Group Ltd to permit this article (if accepted) to be published in BMJ editions and any other BMJPGL products and sublicences such use and exploit all subsidiary rights, as set out in our licence.

Competing interests: All authors have completed the Unified Competing Interest form (available on request from the corresponding author) and declare that the research reported was funded by the Bill and Melinda Gates Foundation. AG, SC, PD are employed by BBC Media Action; one of the entities supporting program implementation. The authors do not have other relationships and are not engaged in activities that could appear to have influenced the submitted work.

For peer review only

Transparency declaration: I, Angela Ng, affirm that the manuscript is an honest, accurate, and transparent account of the study being reported; that no important aspects of the study have been omitted; and that any discrepancies from the study as planned (and, if relevant) have been explained.

Ethics: Institutional Review Boards from the Johns Hopkins Bloomberg School of Public Health in Baltimore, Maryland USA and Sigma Research and Consulting in Delhi, India provided ethical clearance for study activities. Verbal informed consent was obtained from all study participants.

Funding: Bill and Melinda Gates Foundation Role of study sponsors: The study sponsors had no role in the design, implementation or interpretation of study findings. The study team were independent from the funders.

Data Sharing Statement: Extra data can be found by emailing Amnesty LeFevre at aelefevre@gmail.com

For peer review only

Abstract (298 of 300)

Objectives: Efforts to understand the factors influencing the uptake of reproductive, maternal and child health, and nutrition (RMNCH&N) services in high disease burden low-resource settings have often focused on face-to-face surveys or direct observations of service delivery. Increasing access to mobile phones has led to growing interest in phone surveys as a rapid, low-cost alternatives to face-to-face surveys. We assess determinants of RMNCH&N knowledge among pregnant women with access to phones and examine the reliability of alternative modalities of survey delivery.

Participants: Women 5-7 months pregnant with access to a phone

Setting: Four districts of Madhya Pradesh, India

Design: Cross-sectional surveys administered face-to-face and within two weeks, the same surveys were repeated among two random sub-samples of the original sample: face-to-face (n=205), and caller attended telephone interviews (n=375). Bivariate analyses, multivariable linear regression, and prevalence and bias-adjusted kappa scores are presented.

Results: Knowledge scores were low across domains: 52% for maternal nutrition and pregnancy danger signs, 58% for family planning, 47% for essential newborn care, 56% infant and young child feeding, and 58% for infant and young child care. Higher knowledge (>=1 composite score) was associated with older age; higher levels of education and literacy; nuclear family accommodations; primary health decision making; greater attendance in antenatal care; and satisfaction with ASHA services. Surveys questions had low interrater and intermodal reliability (K<.70) with few exceptions. Questions with low reliability included True/false questions and those with unprompted, multiple response options. Reliability may have been hampered by the sensitivity of the content, lack of privacy, enumerators and respondents profile differences, rapport, social desirability bias, and/or enumerator's ability to adequately convey concepts or probe.

Conclusions: Phone surveys are a reliable modality for generating population level estimates data about pregnant women's knowledge, however, should not be used for individual level tracking.

ARTICLE SUMMARY

Strengths and limitations of this study:

Strengths:

- COVID-19 has accelerated the use of remote data capture strategies, however, few studies have undertaken a rigorous process of tool development inclusive of reliability testing.
- Study findings suggest that phone surveys can be a reliable modality for measuring knowledge among pregnant women in rural India at a population level.
- To optimize reliability, limit the use of questions which are sensitive, or have open-ended or multi-response options.

Limitations:

- The sample included pregnant women with reported access to a phone during the day; a population arguably more advantaged than women without access to a phone which could limit the generalizability of findings.

For peer review only

INTRODUCTION

COVID-19 has accelerated the demand for remote data capture solutions, including phone surveys. There is a growing importance and reliance on remote data collection including phone surveys (in India and elsewhere) as COVID has impacted the ease and budgets for data collection (1). Recommendations on questions to consider in determining the suitability of phone surveys to measure varied outcomes is emerging (2). However, despite an increasing body of literature on methods (3,4), including sampling (5, 6), ethics and obtaining consent (5,7,8), improving response rates and other performance metrics (9), significant gaps persist in the standards for developing phone survey tools.

Efforts to measure health practice, prevention, and careseeking in India have most commonly been done via face-to-face survey methods, including the population-based National Family Health Surveys (NFHS) carried out approximately every 5 to 7 years (10,11). In addition to being costly and time intensive, these population based surveys are limited by lengthy recall bias (12). Phone surveys have the potential to be administered more proximally to event of focus, at lower cost, with more minimal resources, and with potentially less burden to the beneficiary.

In 2019-20, NFHS survey findings stated that 54% of women have a mobile phone that they themselves use (13). Growing access to mobile phones at a household level, and more specifically among women, provides an opportunity to develop alternative modalities for the routine collection of data on health behaviours and knowledge. Despite their immense potential, few applications of phone surveys have been reported for measuring health knowledge, behaviours and outcomes in India (11) and even less information is available on the methods influencing the development of these tools. As phone ownership and access increase in India (14), a reliable phone survey tool would enable timely, routine and low cost measurement of the population health status, including knowledge, careseeking, and practices (15).

In this manuscript, we outline the process for developing and refining a phone survey tool for the measurement of reproductive, maternal and newborn health and nutrition (RMNCH&N) knowledge among pregnant women in four districts of Madhya Pradesh. We start by developing overall and domain-specific RMNCH&N knowledge scores and then identify the determinants of higher knowledge scores. We then assess differences in the reliability of the knowledge questions over different modalities including face-to-face surveys at two time points (hereafter called test-retest) and caller attended telephone interviews (CATI; hereafter called phone surveys). This study illuminates gaps in RMNCH&N knowledge among pregnant women, determinants of RMNCH&N knowledge levels, and the reliability of phone survey tools compared to face-to-face tools.

METHODS

Study Setting

Data collection activities were carried out from August – October 2018 in four districts (Hoshangabad, Mandsaur, Rewa, and Rajgarh) of Madhya Pradesh. Madhya Pradesh is a state in central India with a population of over 75 million, predominantly Hindu, and based in rural areas (16). Significant gaps between men and women persist with regard to literacy (82% of men are literate as compared to 59% of women), and only 29% of women report having access to mobile phones that they themselves can use (17). For nearly all health indicators, Madhya Pradesh falls below national averages. Despite near universal attendance of at least one antenatal care visit during pregnancy, only 53% of women receive services within the first trimester, and 36% receive the recommended four visits (17). While institutional delivery rates have increased over time, 20% of women deliver outside the formal health sector, and only 18% receive a health check after birth from a trained provider within 2 days of birth (17). Among children, 51 per 1,000 live births will die prior to their first birthday and 65 per 1,000 live births will die prior to their fifth birthday (17). High mortality rates are affected by undernutrition (43% of children are under weight, 42% are stunted), low rates of exclusive breastfeeding (58%), and gaps in access to basic health services including

For peer review only

immunization. Where the latter is concerned, only 73% of children 12-23 months have received the recommended 3 doses of the DPT vaccine (17).

Study design and sampling

The target population was women 5-7 months pregnant at randomization, ≥ 18 years of age, speak and understand Hindi, and own or have access to a mobile phone during the morning and afternoon. Survey data and sampling technique were captured as part of baseline survey activities conducted as part of the impact evaluation of the Kilkari mobile health intervention described in detail elsewhere (18). A face-to-face cross-sectional survey was conducted among (n=5,095) women with reported access to a mobile phone during the day (Survey Activity A in Figure 1). The survey sample size was powered to detect a 5% change in the practice of exclusive breastfeeding from baseline to endline (12 months later), assuming an alpha of 0.05, power of 0.80, 20% loss to follow-up, and 35% loss of women due to changes in mobile phone numbers (SIM change). Women excluded included BSNL mobile subscribers due to poor network coverage and those who did not provide consent to participate in the trial. Front line health workers in the study villages and census of villages produced the list of eligible women. The sample was randomized and stratified by gestational age, parity, age of woman, and ownership of phone in Stata using the sample command (19).

Within two weeks of the original survey, two separate surveys were conducted on random samples of these women: test-retest (n=205), and phone surveys (n=375). To assess reproducibility in the surveys, stability of the instrument, and inter-rater reliability, a subsample of women who completed the main survey was re-interviewed face-to-face to assess the inter-rater reliability of the responses from the main survey (Survey Activity B in Figure 1). In order to develop a validated phone survey tool, pregnant women who previously completed a face-to-face interview and were not interviewed in the test-retest phase were interviewed a second time over the phone to assess the inter-modal reliability of the responses from the main survey (Survey Activity C in Figure 1).

Each sample size was calculated assuming a kappa of 0.80, margin of error 0.05, and alpha of 0.5, proportion of positive responses of 0.35 for rater 1 and 0.40 for rater 2: 146 participants were needed. To accommodate a 15% loss to follow up or refusal for the test-retest, a minimum of 168 women were targeted and ultimately 205 interviewed. Assuming a 20% response rate for the phone survey, a random sample of 880 women were contacted within 1-2 weeks of the initial face-to-face interview, and ultimately 375 completed the interviews.

Data collection

The survey included seven modules on: 1. mobile phone ownership, use, and digital literacy; 2. pregnancy, delivery, child health and family planning knowledge; 3. birth history; 4. experiences during pregnancy care; 5. content of pregnancy care; 6. delivery and postpartum care intentions; and 7. client satisfaction with accredited social health activists (ASHA) services. Survey questions were drawn from standardised national survey tools, the literature and expert judgment and enhanced through cognitive testing and piloting (18, 20). This manuscript focuses on knowledge; findings from other modules are reported elsewhere (18, 21).

Female enumerators conducted face-to-face and phone surveys. The phone surveys were conducted out of the National Health Systems Resource Center in New Delhi. Surveyors had a minimum of high school education and received training on tool content, and survey implementation, including informed consent. Supervisors provided day to day support and oversight.

Data analysis

Data were analysed using Stata 15 (Stata Corporation, College Station, TX, USA). First, a conceptual framework was developed comprised of factors believed to influence pregnant women's knowledge from

For peer review only

previous literature (Figure 2) (18-35). At the individual level, characteristics such as phone ownership, health care characteristics, socioeconomic, and sociodemographic factors were considered. A wealth index was created by dividing the women into 5 quintiles using principal component analysis. At the community level, social gender norms were analysed such as participation in self-help groups, women being the primary health decision maker, and family living structure as well as geographic characteristics of the community.

In order to best categorize knowledge among pregnant women, survey questions were consolidated into five thematic domains: 1. Maternal nutrition and pregnancy danger signs, 2. Family planning, 3. Essential newborn care, 4. Infant and young child feeding, and 5. Infant and young child care (Supplementary Table 1). For each question, if there was only one correct answer, a binary score was given: 1 if the women knew the correct answer, 0 if not. If there were multiple correct answer options, each correct option was weighted so if all correct options were selected, the final score for that question would be 1. Each question then had equal weight in its category. All the knowledge domains were averaged together at the end to create a final composite score.

A simple linear regression was performed on the final composite score with the factors thought to have an impact on them. A multiple linear regression was performed to see all together which factors would have an impact on overall women's knowledge. In order to find the best model, only variables with a p-value of 0.20 or less during bivariate analyses were used. A multiple linear regression was also performed on each individual knowledge domain to see if particular factors influenced one category more than another.

If the difference in prevalence between survey questions was <15%, kappa scores were used to assess the reliability of the question. Questions were deemed unreliable if the difference in prevalence between survey questions was >15% or if the Cronbach's alpha was 0.7 or higher (22). Kappa coefficients were adjusted for the difference in prevalence levels using the Prevalence Adjusted Bias Adjusted Kappa (PABAK) score. This was to determine if the questions themselves were reliable, and if so, if the question was reliable over the phone. If the threshold of 0.7 was met for just the test-retest, the question was deemed to be reliable for face-to-face surveys in this context. If the threshold of 0.7 was met for both test-retest and phone survey, the question was deemed reliable for phone surveys in this context.

Patient and public involvement

Pregnant women were first engaged upon identification in their households as part of a household listing carried out in mid/late 2018. A small number of pregnant women were involved in the refinement of survey tools through qualitative interviews, including cognitive interviews, which were carried out to optimise survey questions, including the language and translation used. COVID-19 and associated travel restrictions prevented further engagement with women interviewed to disseminate of study findings.

Ethics Approval

Ethical approval for this research study was obtained through Johns Hopkins School of Public Health Institutional Review Board in Baltimore, Maryland, as well as Sigma Research and Consulting in New Delhi, India. All participants consented to the study prior to interview.

RESULTS

Pregnant Women Characteristics

Among 5,095 interviewed through the face-to-face survey, 95% were Hindu, 58% were between 18-24 years of age, 68% had at least one child, 47% belonged to other backward castes, 8% belong to a self-help group, 41% had completed high school or higher, and 57% were able to read the whole sentence (Table 1). A majority of the women interviewed resided in Rewa (57%), followed by Mandsaur, Hoshangabad, and Rajgarh falling at 16%, 8%, and 19% respectively.

Table 1. Characteristics of pregnant women drawn from a face-to-face survey in 4 districts of Madhya Pradesh.

For peer review only

Characteristic	n	Frequency
**Age**		
18-24	2,975	58.4%
25-34	2,023	39.7%
35+	97	1.90%
**Religion**		
Hindu	4,848	95.2%
Muslim	241	4.73%
Other	6	0.12%
**Caste**		
General	1,133	22.2%
Other Backward Caste	2,386	46.8%
Scheduled Caste/Tribe	1,576	30.9%
**Parity (>=1 Child)**	3,486	68.4%
**Employed**	1,921	37.7%
**Education**		
No School	548	10.8%
Primary or less	876	17.2%
Middle School	1,560	30.6%
High School	1,666	32.7%
Higher Education	445	8.70%
**Literacy**		
Cannot read at all	1,659	32.6%
Able to read only parts of sentence	554	10.9%
Able to read whole sentence	2,880	56.6%
**Nuclear family structure**	1,092	21.6%
**Self Help Group**	380	7.46%
**Woman is the primary decision maker for health decisions**	1,451	28.5%
**Attendance of maternity care services**		
Antenatal care 1	742	14.6%
Antenatal care 2	1,225	24.0%
Antenatal care 3	952	18.7%
Antenatal care 4+	2,176	42.7%
**Satisfied with the services provided by the ASHA**	4,699	92.2%
**Owns Phone**	3,860	75.8%
**District**		
Hoshangabad	406	7.97%
Mandsaur	821	16.1%
Rewa	2,920	57.3%
Rajgarh	948	18.6%

Survey reliability

For each survey question, inter-rater reliability was assessed by comparing reported results from face-to-face surveys conducted at two time points (test-retest; n=205), while intermodal reliability was assessed by comparing findings from the face-to-face survey to phone surveys (n=375). Table 2 presents prevalence and reliability estimates by question.

Overall results suggest that population level estimates of knowledge over time and across modalities were similar, but reliability among individual women in the sample was poor. Prevalence estimates illustrate the latter; demonstrating that women's knowledge across and within domains was low. Questions with low reliability included True/false questions and those with unprompted, multiple response options.

For peer review only

Figure 3 graphs the correlation between the prevalence and bias adjusted kappa statistics for face-to-face surveys against the in-person re-test and the phone survey. The correlation forms a positive linear line, representing a strong positive reliability between the face-to-face and phone survey options.

For peer review only

Table 2: Prevalence and reliability estimates for knowledge questions among pregnant women

Question text	Response Options	Baseline Prevalence	Retest Prevalence	Phone Prevalence	Retest Kappa	Phone Kappa
**Domain: Maternal nutrition and pregnancy danger signs**						
What foods should you eat during pregnancy?	Fish	0.07	0.08	0.02	0.79	0.84
	Meat	0.08	0.06	0.04	0.80	0.83
	Eggs	0.13	0.08	0.05	0.79	0.68
	Milk/Dairy Products	0.73	0.79	0.59	0.32	0.17
	Fruits	0.89	0.92	0.73	-	-
	Green leafy vegetables	0.94	0.93	0.76	-	-
	Pulses and Nuts	0.86	0.88	0.34	-	-
What should you not eat or drink during your pregnancy?	Alcohol	0.03	0.02	0.01	0.94	0.93
	Coffee	0.03	0.01	0.05	0.95	0.84
	Cigarettes	0.05	0.02	0.01	0.95	0.83
	Tea	0.14	0.16	0.09	0.58	0.45
Anaemia (khoon ki kami) in pregnancy can affect the growth and development. What should you do if you are anemic?	Take IFA tablet daily	0.64	0.73	0.56	0.30	0.21
	Tea	0.40	0.63	0.07	-	-
	Cigarettes/bids	0.83	0.76	0.34	-	-
How many tetanus injections should you have during pregnancy?	2 shots	0.66	0.52	0.65	0.77	0.58
What are Iron Folic Acid tablets?	Help prevent/treat anemia	0.80	0.90	0.64	-	-
	Improve the health/well-being of my baby	0.73	0.85	0.43	-	-
What danger signs during pregnancy and before labor starts would lead you to go to the health facility immediately?	Yellowing of skin	0.10	0.02	0.01	0.88	0.78
	Vaginal Bleeding	0.31	0.32	0.10	-	-
	Vaginal Discharge	0.35	0.33	0.10	-	-
	Convulsions	0.42	0.52	0.09	-	-
	Stomach Cramps	0.87	0.95	0.45	-	-
	Swelling on limbs and face	0.38	0.30	0.20	-	-
**Domain: Infant and young child feeding**						
How many times per day should newborns be breastfed?	9-10 times a day, on demand	0.94	0.94	N/A	0.75	N/A
What are some of the benefits of breastfeeding?	It helps to maintain space between children	0.01	0.01	0.01	0.98	1.00
	Reduces expenditure on medical care as child will fall sick a fewer number of times	0.11	0.05	0.05	0.82	0.62
	The more the child breastfeeds, the more milk will be produced	0.07	0.09	0.02	0.67	0.91

For peer review only

	Promotes Mother-Baby bonding	0.09	0.01	0.27	-	-
	Help build immunity for your child	0.72	0.71	0.57	-	-
	Promotes child growth, wellness	0.85	0.96	0.54	-	-
How soon after delivery should you give foods or liquids other than mothers milk?	6 months	0.83	0.74	0.75	0.64	0.64
	Immediately	0.28	0.64	0.15	-	-
What types of foods should a baby be given after 6 months of age?	Cheese	0.05	0.07	0.01	0.73	0.85
	White potatoes, roots	0.01	0.00	0.09	0.99	0.83
	Ripe mangoes, papayas	0.05	0.10	0.05	0.71	0.82
	Beans, peas, nuts	0.14	0.08	0.02	0.75	0.52
	Bread, roti, grains	0.17	0.08	0.14	0.55	0.54
	Fruits or vegetables	0.14	0.16	0.16	0.52	0.63
	Baby food	0.42	0.39	0.47	0.05	0.07
	Plain Water	0.74	0.76	0.37	-	-
	Juice	0.10	0.07	0.35	-	-
	Lentil Broth/soup	0.18	0.14	0.73	-	-
	Milk	0.78	0.90	0.52	-	-
	Pumpkin, squash	0.78	0.88	0.38	-	-
	Solid, semi-solid, soft food	0.27	0.45	0.00	-	-
	Liver	0.00	0.00	0.00	-	-
	Chicken	0.00	0.00	0.00	-	-
	Meat	0.00	0.00	0.00	-	-
	Eggs	0.00	0.00	0.00	-	-
	Dried Fish	0.00	0.00	0.00	-	-
**Domain: Infant and young child care**						
What danger signs do you know about for the newborn after delivery?	Redness	0.01	0.01	0.01	0.98	0.99
	Red eyes	0.02	0.02	0.01	0.94	0.96
	Skin lesions	0.03	0.02	0.01	0.87	0.96
	Blueness of hands	0.03	0.01	0.01	0.96	0.91
	Convulsions	0.04	0.02	0.06	0.85	0.84
	Low birth weight	0.05	0.05	0.04	0.83	0.74
	Lethargy	0.09	0.17	0.06	0.51	0.74
	Difficulty feeding	0.22	0.17	0.16	0.50	0.28
	Yellow color of skin	0.17	0.23	0.04	0.50	0.61
	Pitched cry	0.26	0.21	0.30	0.44	0.20
	Difficulty breathing	0.36	0.35	0.05	-	-
	Baby feels hot or cold to touch	0.30	0.49	0.15	-	-
	Fever	0.83	0.93	0.34	-	-

For peer review only

	Vomiting	0.52	0.49	0.24	-	-
How soon after your baby is born should it receive its first vaccination?	At Birth	0.74	0.8	0.73	0.52	0.54
	Within 1 month	0.09	0.11	0.09	-	-
	1-2 months	0.04	0.01	0.02	-	-
	2+ months	0.02	0.01	0.02	-	-
What are things you can do to prevent your child from getting diarrhea?	Give baby safe drinking water > 6 months	0.17	0.08	0.05	0.68	0.65
	Exclusively breastfeed children < 6 months	0.14	0.06	0.05	0.69	0.63
	Cover water and food to avoid flies sitting on it	0.16	0.23	0.1	0.39	0.55
	Safe disposal of stools	0.09	0.11	0.12	0.57	0.49
	Make sure the environment is clean	0.50	0.63	0.38	0.02	-0.02
	Wash hands	0.39	0.40	0.20	-	-
What should you give your child to treat diarrhea?	Intravenous	0.01	0.01	0.01	0.96	0.98
	Antibiotic	0.03	0.00	0.01	0.96	0.95
	Injection	0.07	0.18	0.01	0.50	0.80
	Home remedy	0.10	0.02	0.01	0.93	0.78
	Salt and sugar	0.06	0.04	0.13	0.86	0.71
	ORS + Zinc	0.11	0.11	0.18	0.70	0.50
	ORS	0.40	0.48	0.27	0.38	0.24
	Antidiarrheals	0.39	0.41	0.05	-	-
	Other pill or syrup	0.62	0.69	0.38	-	-
What are three critical times for a woman to wash her hands?	After defecation	0.98	0.94	0.43	-	-
	Before cooking or handling food	0.91	0.94	0.37	-	-
	Before eating or feeding the child	0.89	0.92	0.37	-	-
**Domain: Family Planning**						
How soon after you give birth, can you get pregnant again?	Immediately	0.02	0.01	0.04	0.43	-0.02
	Not until menses return	0.73	0.8	0.12	-	-
	After you stop breastfeeding	0.03	0.01	0.14	-	-
What is the recommended length of time you should wait between having another child?	Immediately	0.00	0.00	0.00	0.61	0.36
	Wait for at least 1 year	0.02	0.02	0.02	-	-
	Wait for at least 2 years	0.19	0.27	0.18	-	-
	Wait for at least 3 years	0.67	0.64	0.51	-	-
What are the benefits of using family planning to limit the size of your family?	Easy way to control the size of your family	0.48	0.47	0.46	0.02	0.00
	Financial savings	0.50	0.51	0.50	0.01	0.06
	Give you more time to take care of the children you already have	0.70	0.82	0.54	-	-

For peer review only

Men become physically weak after accepting sterilization	FALSE	0.15	0.14	0.09	0.28	-0.02
There are many safe methods of birth control	TRUE	0.94	0.96	0.33	-	-
Female sterilization can be done at the time of birth	TRUE	0.76	0.82	0.21	-	-
Male sterilization is an easy way to control family size	TRUE	0.72	0.63	0.22	-	-
PPIUCD insertion and female sterilization	TRUE	0.87	0.86	0.35	-	-
Do you know of a place where you can obtain a method of family planning?	Yes	0.9	0.98	0.38	-	-
Which ways or methods of contraception have you heard about?	Female sterilization	0.92	0.97	0.37	-	-
	Male Sterilization	0.49	0.54	0.21	-	-
	IUD	0.47	0.36	0.31	-	-
	Oral contraceptive pills	0.84	0.93	0.58	-	-
	Injectables	0.70	0.78	0.28	-	-
	Male Condom	0.64	0.74	0.46	-	-
	Rhythm Method	0.56	0.45	0.02	-	-
	Withdrawal	0.24	0.08	0.05	-	-
**Domain: Essential newborn care**						
How do you keep the baby warm after delivery?	Put the baby on your chest	0.17	0.15	0.13	0.5	0.49
	Dried or wiped soon after birth	0.25	0.30	0.24	0.08	0.24
	Cover in clothes	0.96	1.00	0.56		
What should you put on the cord after delivery?	Nothing	0.06	0.05	0.04	0.82	0.77
	Blade used for other purposes	0.72	0.72	0.05	-	-
	Scissors	0.41	0.72	0.29	-	-
	Knife	0.02	0.03	0.18	-	-
	Surgical Blade	0.03	0.01	0.39	-	-
	New Razor blades	0.72	0.71	0.11	-	-
How soon after delivery should your baby be bathed?	1 Day	0.8	0.77	0.03	0.49	0.8

For peer review only

Determinants of RMNCH&N knowledge

Figure 4 shows average knowledge scores for the five composite knowledge domains: maternal nutrition and pregnancy danger signs (52%), family planning score (58%), immediate post-birth care for the neonate (47%), infant and young child feeding (56%), and infant and young child care (58%). The average of all domains was used to create a final composite score (54%).

Table 3 presents results from bivariate and multivariate regressions on factors influencing pregnant women's knowledge (n=5,095). In the multivariable model, women's knowledge was significantly higher among older women (25-34 years, $p<0.001$; >34 years, $p=0.02$); with higher education ($p=0.009$); able to read a full sentence ($p<0.001$); living in a nuclear family ($p<0.001$); who reported being the primary decision maker in health decisions ($p<0.001$); had attended a greater number of ANC visits ($p<0.001$); and reported being satisfied with ASHA services ($p<0.001$). Women from the rural districts of Mandsaur and Rajgarh had significantly lower knowledge scores than women in the reference peri-urban district of Hoshangaband. Women's phone ownership, socioeconomic status, and caste were not associated with significant differences in knowledge.

Table 3. Factors associated with pregnant women's RMNCH&N knowledge in four districts of Madhya Pradesh, India

Variable	Unadjusted Coefficient (95% Confidence Interval)	p-value	Adjusted Coefficient (95% Confidence Interval)	p-value
**Age**				
18-24	1	-	1	--
25-34	3.21(2.68-3.74)	<0.001	1.91(1.35-2.46)	<0.001
35+	1.87(-0.04-3.78)	0.055	2.04(0.34-3.74)	0.02
**Parity (>=1 child)**	4.40(3.85-4.96)	<0.001	4.39(3.74-5.03)	<0.001
**Caste**				
General	1	-	1	-
Other backwards	-0.62(-1.30-0.05)	0.070	0.62(-0.06-1.3)	0.076
Scheduled	-0.97(-1.70-0.24)	0.010	0.24(-0.58-1.06)	0.567
**Wealth Index**				
Q1	1	--	1	--
Q2	0.30(-0.52-1.13)	0.471	0.32(-0.49-1.13)	0.442
Q3	0.03(-0.80-0.86)	0.938	0.14(-0.78-1.07)	0.765
Q4	0.25(-0.58-1.08)	0.551	0.16(-0.83-1.16)	0.751
Q5	1.83(1.00-2.66)	<0.001	0.72(-0.38-1.82)	0.197
**Education level**				
No School	1	--	1	--
Primary or Less	0.71(-0.31-1.72)	0.172	0.65(-0.35-1.65)	0.201
Middle School	1.32(0.39-2.24)	0.005	0.6(-0.51-1.72)	0.289
Higher Education	3.26(2.37-4.16)	<0.001	1.65(0.42-2.89)	0.009
**Literacy**				
Cannot read at all	1	--	1	-
Reading only parts of sentence	1.43(0.52-2.34)	0.002	0.95(0.01-1.9)	0.049
Read whole sentence	3.02(2.44-3.59)	<0.001	2.29(1.42-3.15)	<0.001
**Self Help Group**	0.97(-0.03-1.97)	0.057	0.4(-0.42-1.22)	0.335
**Nuclear Family Structure**	-1.97(-2.61--1.33)_	<0.001	-2.14(-2.95--1.33)	<0.001
**Women is the primary decision maker in health decisions**	2.03(1.45-2.61)	<0.001	1.59(1-2.19)	<0.001
**Number of ANC Visits**				

For peer review only

1	1	--	1	--
2	1.13(0.27-2.01)	0.011	1.03(0.16-1.9)	0.021
3	1.64(0.73-2.56)	<0.001	1.95(1.04-2.85)	<0.001
4	1.14(0.34-1.94)	0.005	1.68(0.81-2.54)	<0.001
**Satisfied with the services provided by the ASHA**	2.61(2.02-3.19)	<0.001	2.18(1.57-2.79)	<0.001
**Phone Ownership**	1.31(0.71-1.93)	<0.001	0.61(-0.01-1.22)	0.055
**District**				
Hoshangabad	1	--	1	--
Mandsaur	-3.30(-4.42--2.17)	<0.001	-3.3(-4.51--2.09)	<0.001
Rewa	-1.53(-2.52--0.55)	<0.001	-0.13(-1.21-0.95)	0.814
Rajgarh	-4.65(-5.75--3.55)	<0.001	-3.05(-4.21--1.89)	<0.001

Supplementary Table 2 explores determinants of knowledge for each of the five domains. Across all domains, knowledge was significantly higher among older women (25-34 years of age), with a least one child, able to read a whole sentence, and who reported being satisfied with services provided by the ASHA. Knowledge of maternal nutrition and pregnancy danger signs was significantly higher amongst women living in a nuclear family, with higher education, and who had attended a greater number of antenatal care visits. Family planning knowledge was significantly higher amongst women in the highest socioeconomic strata, with primary school or higher education. Knowledge of immediate post-birth care for the newborns was similarly higher amongst women in the highest socioeconomic strata, and with higher education.

DISCUSSION

The study sought to develop and refine a phone survey tool for the measurement of RMNCH&N knowledge among pregnant women in four districts of Madhya Pradesh. Findings suggest that there is a correlation between face-to-face and re-test reliability and face-to-face and phone reliability. Instances were not observed where there was re-test reliability for face-to-face and poor reliability for face-to-face versus phone. Questions with unprompted and more possible response options (e.g. foods to eat during pregnancy) had similarly poor inter-rater and inter-modal reliability as questions that sought to pin respondents down to one answer (e.g. How many tetanus injections should you have during pregnancy?). This suggests that the questions themselves may not be well suited to repeat questioning. Overall knowledge levels were low for most questions asked. This underscores the need to bolster awareness among women and improve access to health information. It too may indicate that some questions and response options used were not culturally appropriate or relevant for this context (e.g. alcohol, coffee, and cigarettes are uncommonly consumed in rural India by women which may explain the low awareness of these items when asked "What should you not eat or drink during your pregnancy?"). The collective body of findings suggests that survey estimates may be appropriate for ascertaining population level estimates rapidly, however, contraindicated in instances where longitudinal tracking or individual level analyses are required. This may have critical implications for those looking to utilize remote surveys for measuring intermediate program outcomes, including knowledge.

Study findings contribute to the limited body of literature on how to optimise the development of phone surveys for use in the measurement of population level knowledge and health outcomes. Few studies to date have sought to refine phone survey tools through a rigourous process of reliability testing (23). Findings from a 2017 systematic review identified only 10 studies which have reported using reliability testing to develop phone survey tools; five of these included face-to-face and CATI survey comparisons (23). In Honduras and Peru (24), comparisons were made across independent samples, while in Lebanon (25) and two separate surveys in Brazil (26), comparisons were made using the same sample across two modalities (as in our study) (18, 22). Overall findings broadly suggest that there was concordance in the results across CATI and face-to-face modalities (21). However, for some outcomes, prevalence estimates

For peer review only

did differ across modalities (27). Studies in Honduras and Peru compared additional phone survey modalities, including text messages and interactive voice response (IVR), and concluded that CATI surveys had the lowest discordance with the face-to-face surveys (24). Elsewhere, more recent efforts have sought to compare the reliability of alternative phone survey delivery modalities (IVR versus CATI) for measuring non-communicable disease risk factors (28). In our context, low literacy rates precluded testing phone survey modalities other than CATI surveys.

Underpinning efforts to rigorously develop the phone survey tool was a design to measure knowledge among pregnant women. Overall knowledge levels among pregnant women in these four districts of Madhya Pradesh ranged from 47% for essential newborn care to 58% for family planning. Areas of lower knowledge included what should be put on the cord after delivery, how to keep the baby warm after delivery, what foods to eat during pregnancy, what food and drinks to avoid during pregnancy, etc. Knowledge scores were significantly higher amongst older women; those with higher levels of education and literacy; living in a nuclear family; who reported being the primary decision maker in health decisions; who had attended a greater number of ANC visits; and reported being satisfied with ASHA services.

Other studies have measured women's knowledge levels on topics such as neonatal danger signs, immediate post-birth care for the neonate, infant and young child feeding, breastfeeding, and family planning. Compared to 18% of women in Ethiopia and 15% of women in Uganda who have good knowledge of neonatal danger signs, our women have a higher relative knowledge at 58% (29,30). In a survey among hospital-delivered mothers in Sri Lanka, while 90% of mothers knew about advantages of breastfeeding compared to our survey of 56%, and only 22% correctly answered what should be applied to an umbilical cord compared to our survey at 47% (31). In India, universal knowledge existed among married women in Uttar Pradesh about at least one family planning method and 90% of women in Lucknow were aware of contraceptive methods in family planning existed comparatively to our study's lower family planning knowledge at 58% (32,33). This is all subject to each study's own verified survey methods and composite score generation. Developing one standardized survey questionnaire across studies to measure women's RMNCH&N knowledge would lend more insight into how comparative this data is.

There is limited empirical evidence of factors influencing pregnant women's knowledge. A study conducted in Delhi assessed knowledge components that included immunization against tetanus, number of antenatal visits, folic acid tablets, danger signs during pregnancy or labour, and discussions with elders, mothers-in-law, doctors, or friends (34). Like our study, it found that women in joint families were also significantly more likely to have higher knowledge (34). Another study in Rewa district also found that women whose husbands and in-laws played a dominate role in decision making had lower knowledge of key danger signs during pregnancy (35). This is consistent with our study, which included Rewa district among our four study districts, as we found if the women played a role in the decision-making process, she had significantly higher knowledge of maternal danger signs. In a Uganda study, no association was found with attending ANC visits and knowledge of danger signs, which is inconsistent with our findings (30). Surprisingly, while NFHS found that more family planning methods were seen in women of higher wealth index, wealth was not found to be a determinant in this study (11). Taken together, our findings suggest to increase women's knowledge in the future, one should empower women to play a larger role in the health decision making process, strengthen ASHA services, promote education, and increase access and uptake of ANC services. Further research to understand sources and quality of sources for knowledge should be undertaken as well.

Limitations

This survey was limited to RMNCH&N knowledge in women 5-7 months pregnant with access to a mobile phone during the day, in four predominately rural districts of Madhya Pradesh. This may have led to a pro-rich bias in the sampling given the profile of beneficiaries with access to mobile phones (36). Survey questions were derived from expert review and large national survey tools, including the demographic and health survey for India and the Multiple Indicator Cluster Survey (MICS). Cognitive interviews were used

For peer review only - http://bmjopen.bmj.com/site/about/guidelines.xhtml

to refine questions on respectful maternity care; a process described elsewhere (19, 20, 37). Ideally the same process would have been followed for refining knowledge questions. However, budget and time constraints made this added step infeasible. Phone surveys were administered by Delhi based enumerators whose demographic profile differed from respondents. Alternative phone survey modalities, including text messages, interactive voice response, and USSD were considered but deemed less desirable to using computer assisted telephone interviews (CATI) surveys which could allow beneficiaries to speak with and ask for clarification questions.

CONCLUSIONS

Study findings offer insight into the challenges associated with reliably measuring RMNCH&N knowledge among pregnant women using face-to-face and remote survey tools. Population level estimates of knowledge over time and across modalities were similar, but reliability among individual women in our sample was poor. This suggests that the use of phone surveys to measure knowledge is contraindicated in instances where longitudinal tracking or individual level analyses are required however it may be appropriate as a means of gaining population level insights. Among women assessed, overall knowledge levels were low; however, knowledge scores were significantly higher amongst older women; those with higher levels of education and literacy; living in a nuclear family; who reported being the primary decision maker in health decisions; who had attended a greater number of ANC visits; and reported being satisfied with ASHA services.

ACKNOWLEDGEMENTS

We thank the women and families of Madhya Pradesh who generously gave of their time to support this work. We are humbled by the opportunity to convey their perspectives and experiences. We additionally are grateful to Neha Dumke and the team at the National Health Systems Resource Centre for their support. This work was made possible by the Bill and Melinda Gates Foundation. We thank Diva Dhar, Suhel Bidani, Rahul Mullick, Dr. Suneeta Krishnan, Dr. Neeta Goel and Dr. Priya Nanda for believing in us and giving us this opportunity. We additionally wish to thank are grateful to the larger team of enumerators from OPM-India and NHSRC who worked tirelessly over many months to implement the surveys that form the backbone of our analyses. We additionally thank Vinit Pattnaik at OPM and Erica Crawford at Johns Hopkins University for their support to the financial management support of our work.

FIGURE CAPTION

Figure 1. Completed interviews for face-to-face and CATI surveys
Figure 2. Conceptual framework for identifying the determinants of pregnant women's RMNCH&N knowledge
Figure 3. Measurement of degree in which repeated measurements in pregnant women interviewed (test-retest) provide similar answers
Figure 4. Average knowledge scores for composite knowledge domains

REFERENCES

1. McDougal L, Raj A, Yore J, et al. Strengthening Gender Measures and Data in the COVID-19 Era : An Urgent Need for Change (English). 2021. World Bank Group. http://documents.worldbank.org/curated/en/681671619424596227/Strengthening-Gender-Measures-and-Data-in-the-COVID-19-Era-An-Urgent-Need-for-Change
2. Menon S, Sonderegger P, Totapally S. Five questions to consider when conducting COVID-19 phone research. BMJ Global Health 2021;6 :e004917. doi:10.1136/ bmjgh-2020-004917
3. Himelein K, Eckman S, Lau C, et al. Mobile phone surveys for understanding COVID-19 impacts: Part I sampling and Mode [Internet]. World Bank Blogs. Development Impact; [cited 2021Nov13]. Available from: https://blogs.worldbank.org/impactevaluations/mobile-phone-surveys-understanding-covid-19-impacts-part-i-sampling-and-mode
4. Gibson DG, Pereira A, Farrenkopf BA, et al. Mobile phone surveys for collecting population-level estimates

For peer review only

in low- and middle-income countries: A literature review. Journal of Medical Internet Research. 2017;19(5).
5. Kopper S, Sautmann A. Best practices for conducting phone surveys [Internet]. The Abdul Latif Jameel Poverty Action Lab (J-PAL). 2020 [cited 2021Nov13]. Available from: https://www.povertyactionlab.org/blog/3-20-20/best-practices-conducting-phone-surveys
6. Labrique A, Blynn E, Ahmed S, et al. Health surveys using mobile phones in developing countries: Automated Active Strata Monitoring and other statistical considerations for improving precision and reducing biases. Journal of Medical Internet Research. 2017;19(5).
7. Khalil K, Das P, Kammowanee R, et al. Ethical considerations of phone-based interviews from three studies of covid-19 impact in Bihar, India. BMJ Global Health. 2021;6(Suppl 5).
8. Ali J, Labrique AB, Gionfriddo K, et al. Ethics considerations in Global Mobile Phone-based surveys of Noncommunicable Diseases: A conceptual exploration. Journal of Medical Internet Research. 2017;19(5).
9. Gibson DG, Pariyo GW, Wosu AC, et al. Evaluation of mechanisms to improve performance of mobile phone surveys in low- and middle-income countries: Research protocol. JMIR Research Protocols. 2017;6(5).
10. Nair H, Williams LJ, Marsh A, et al. Assessing the reactivity to mobile phones and repeated surveys on reported care-seeking for common childhood illnesses in rural India. Journal of Global Health. 2018;8(2).
11. International Institute for Population Sciences (IIPS) and ICF. 2021. National Family Health Survey (NFHS-5), India, 2019-21. Mumbai: IIPS.
12. Manesh AO, Sheldon TA, Pickett KE, et al. Accuracy of child morbidity data in demographic and health surveys. International Journal of Epidemiology. 2007;37(1):194–200.
13. Mohan D, Bashingwa JJ, Tiffin N, et al. Does having a mobile phone matter? Linking phone access among women to health in India: An exploratory analysis of the National Family Health Survey. PLOS ONE. 2020;15(7).
14. International Telecommunication Union. The World in 2016: ICT Facts and Figures [Internet]. [cited 2019 Mar 9]. Available from: https://www.itu.int/en/ITU-D/Statistics/Pages/stat/default.aspx
15. Okonjo-Iweala N, Osafo-Kwaako P. Improving health statistics in Africa. The Lancet. 2007;370(9598):1527–8.
16. Raju S, Ayyar Panchapagesan N. Madhya Pradesh [Internet]. Encyclopædia Britannica, inc. [cited 2019 Mar 4]. Available from: https://www.britannica.com/place/Madhya-Pradesh
17. National Family Health Survey. State Fact Sheet Madhya Pradesh [Internet]. 2016 [cited 2019 Mar 3]. Available from: http://rchiips.org/nfhs/pdf/NFHS4/MP_FactSheet.pdf
18. LeFevre A, Agarwal S, Chamberlain S, et al. Are stage-based health information messages effective and good value for money in improving maternal newborn and child health outcomes in India? Protocol for an individually randomized controlled trial. Trials. 2019;20(1).
19. LeFevre AE, Scott K, Mohan D, et al. Development of a Phone Survey Tool to Measure Respectful Maternity Care During Pregnancy and Childbirth in India: Study Protocol. JMIR Research Protocols. 2019;8(4)
20. Scott K, Gharai D, Sharma M, et al. Yes, no, maybe so: the importance of cognitive interviewing to enhance structured surveys on respectful maternity care in northern India. Health Policy and Planning. 2019
21. Shah N, Mohan D, Agarwal S, et al. Novel approaches to measuring knowledge among frontline health workers in India: Are phone surveys a reliable option? PLOS ONE. 2020;15(6).
22. Terwee CB, Bot SD, de Boer MR, et al. Quality criteria were proposed for measurement properties of health status questionnaires. J Clin Epidemiol. 2007 Jan; 60(1):34-42.
23. Greenleaf AR, Gibson DG, Khattar C, et al. Building the Evidence Base for Remote Data Collection in Low- and Middle-Income Countries: Comparing Reliability and Accuracy Across Survey Modalities. Journal of Medical Internet Research. 2017;19(5).
24. Ballivian A, Azevedo J, Durbin W, et al. Using mobile phones for high-frequency data collection. In: Toninelli D, Pinter R, de Pedraza P, editors. Mobile Research Methods: Opportunities and Challenges of Mobile Research Methodologies. London: Ubiquity Press; 2015:21-39.
25. Mahfoud Z, Ghandour L, Ghandour B, et al. Cell phone and face-to-face interview responses in population-based surveys: how do they compare? Field Methods 2014 Jul 01;27(1):39-54.
26. Ferreira A, César CC, Malta DC, et al. Validade de estimativas obtidas por inquérito telefônico: comparação entre VIGITEL 2008 e inquérito Saúde em Beagá. Rev bras epidemiol 2011 Sep;14(1):16-30.
27. Francisco P, Azevedo Barros MB, Segri N, et al. Comparação de estimativas para o auto-relato de condições crônicas entre inquérito domiciliar e telefônico - Campinas (SP), Brasil. Rev bras epidemiol 2011 Sep;14(3):5-15.
28. Pariyo GW, Greenleaf AR, Gibson DG, et al. Does mobile phone survey method matter? Reliability of computer-assisted telephone interviews and interactive voice response non-communicable diseases risk factor

For peer review only

surveys in low and middle income countries. PLOS ONE. 2019;14(4).
29. Nigatu SG, Worku AG, Dadi AF. Level of mother ' s knowledge about neonatal danger signs and associated factors in North West of Ethiopia : a community based study. BMC Res Notes. 2015;4–9.
30. Sandberg J, Odberg Pettersson K, Asp G, et al. Inadequate Knowledge of Neonatal Danger Signs among Recently Delivered Women in Southwestern Rural Uganda: A Community Survey. PLoS One. 2014 May 13;9(5):e97253.
31. Senarath U, Fernando D, Vimpani G, et al. Factors associated with maternal knowledge of newborn care among hospital-delivered mothers in Sri Lanka. Transactions of the Royal Society of Tropical Medicine and Hygiene. 2007;101(8):823–30.
32. Singh A, Singh KK, Verma P. Knowledge, attitude and practice GAP in family planning usage: an analysis of selected cities of Uttar Pradesh. Contraception and Reproductive Medicine. 2016;1(1).
33. Sharma V, Mohan U, Das V, et al. Socio demographic determinants and knowledge, attitude, practice: Survey of family planning. Journal of Family Medicine and Primary Care. 2012;1(1):43.
34. Acharya AS, Kaur R, Prasuna JG, et al. Making Pregnancy Safer-Birth Preparedness and Complication Readiness Study Among Antenatal Women Attendees of A Primary Health Center, Delhi. Indian Journal of Community Medicine. 2015;40(2):127.
35. Deoki N, Kushwah SS, Dubey DK, et al. A study for assessing birth preparedness and complication readiness intervention in Rewa District of Madhya Pradesh Chief Investigator, India. Department of Community Medicine, SS Medical College, Rewa, MP. 2008;9.
36. Mohan D, Bashingwa JJ, Tiffin N, et al. Does having a mobile phone matter? Linking phone access among women to health in India: An exploratory analysis of the National Family Health Survey. PLOS ONE. 2020;15(7).
37. Scott K, Ummer O, LeFevre AE. The devil is in the detail: reflections on the value and application of cognitive interviewing to strengthen quantitative surveys in global health. Health Policy and Planning. 2021;36(6):982–95.

For peer review only

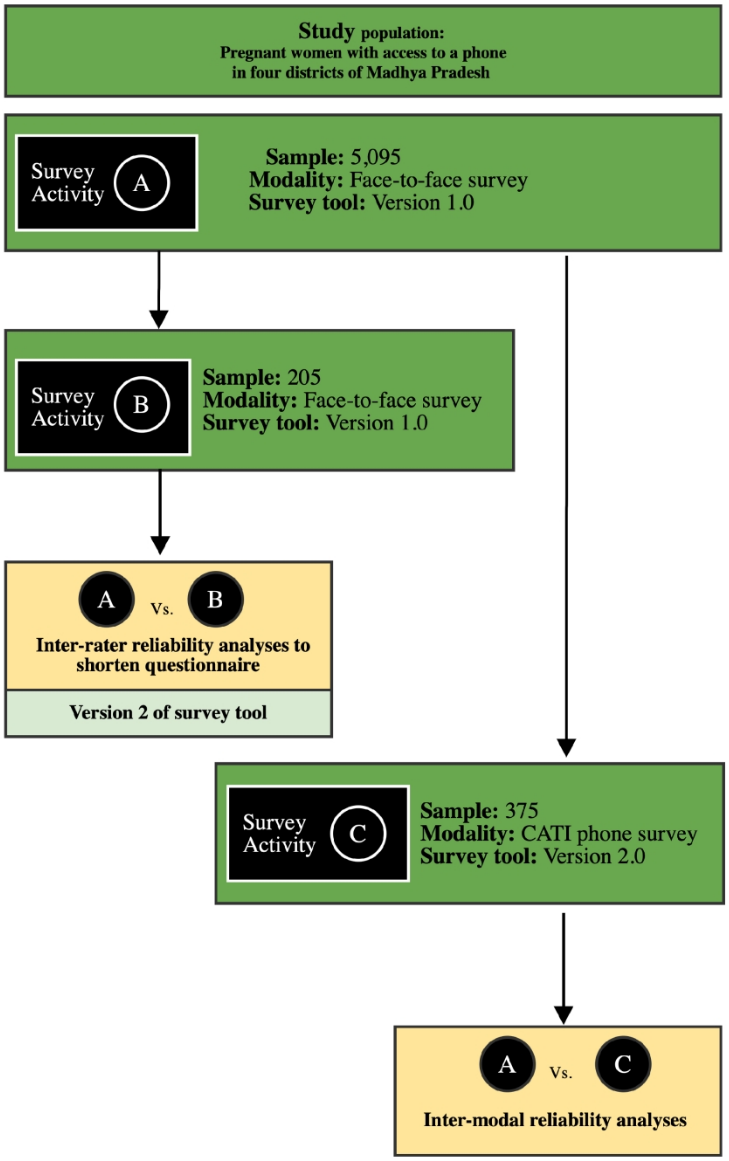

Figure 1. Completed interviews for face-to-face and CATI surveys

177x273mm (300 x 300 DPI)

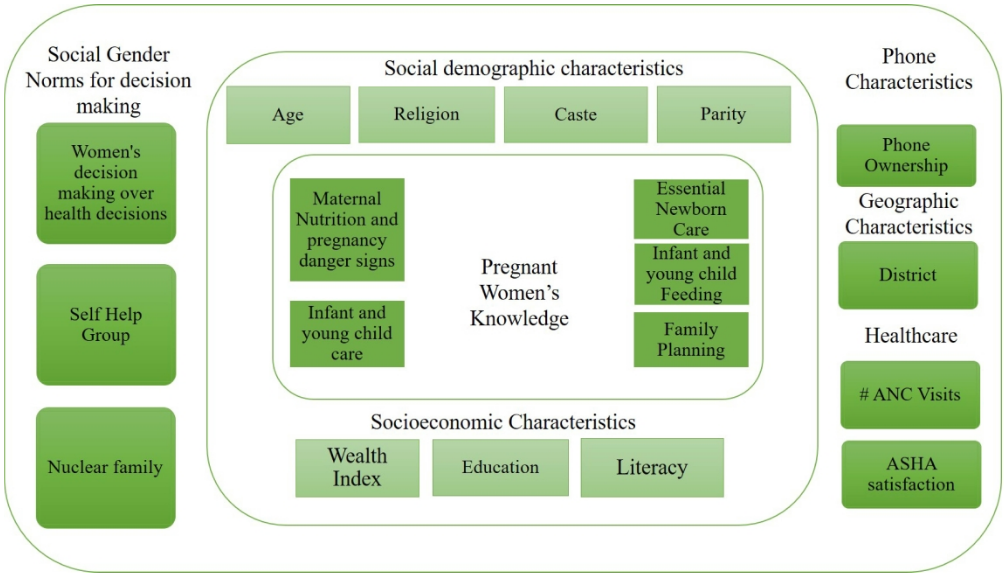

Figure 2. Conceptual framework for identifying the determinants of pregnant women's RMNCH&N knowledge

670x381mm (300 x 300 DPI)

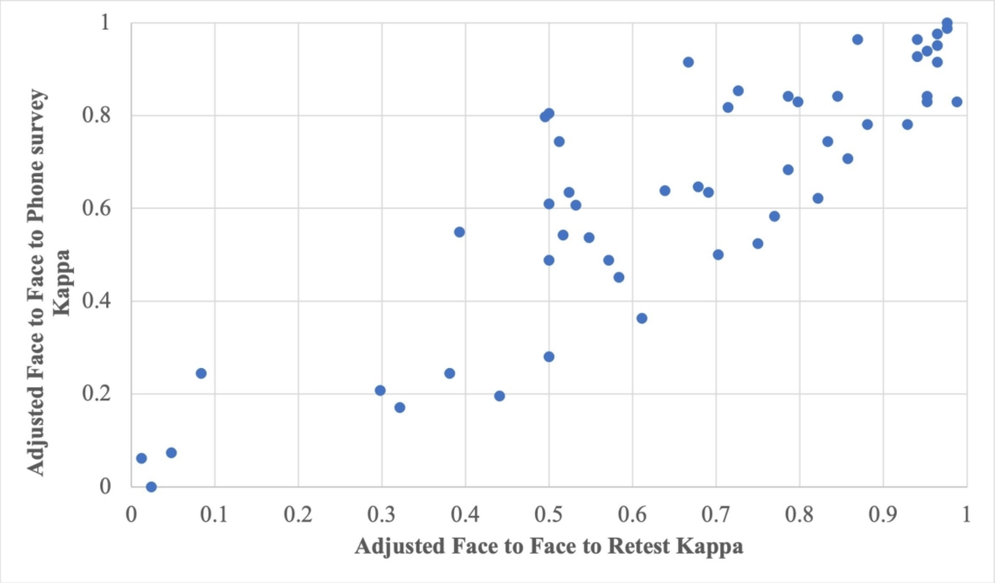

Figure 3. Measurement of degree in which repeated measurements in pregnant women interviewed (test-retest) provide similar answers

180x105mm (300 x 300 DPI)

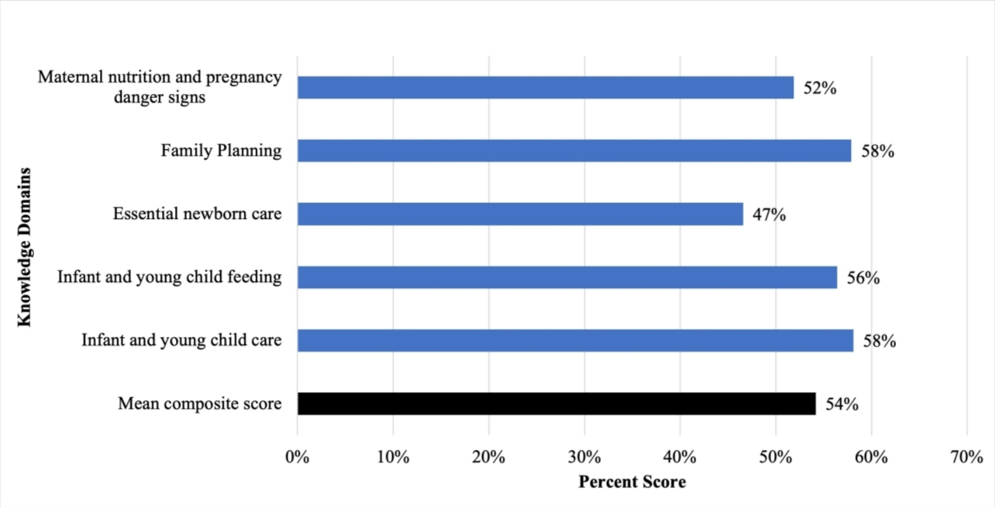

Figure 4. Average knowledge scores for composite knowledge domains

212x109mm (300 x 300 DPI)

Supplementary Table 1: Questions included in each domain of knowledge

Questions	Answer options	Correct answer(s)
**Maternal nutrition and pregnancy danger signs (6 questions)**		Maximum possible score: 6
What foods should you eat during pregnancy?	Unprompted free list, enumerator noted any correct answers mentioned	Milk/dairy products; fruits; green leafy vegetables; pulses and nuts; egg; meat; fish
What should you not eat or drink during your pregnancy?	Unprompted free list, enumerator noted any correct answers mentioned	Tea; coffee; cigarettes/bidis; alcohol
What should you do if you are anemic?	Unprompted free list, enumerator noted any correct answers mentioned	Have your blood checked regularly; take IFA tablets daily; eat well
What are the benefits of taking Iron Folic Acid tablets during pregnancy?	Unprompted free list, enumerator noted any correct answers mentioned	Help prevent/treat anemia; improve the health/well-being of my baby
How many tetanus injections should you have during pregnancy?	Number or don't know	2
What danger signs during pregnancy and before labor starts would lead you to go to the health facility immediately?	Unprompted free list, enumerator noted any correct answers mentioned	Vaginal bleeding; vaginal discharge; convulsions; stomach cramps; swelling on limbs and face; yellowing of skin; fever; decreased or absent fetal movements
**Immediate post-birth care for the neonate (4 questions)**		Maximum possible score: 4
How soon after delivery should your baby be bathed?		
How should you keep the baby warm after delivery?	Unprompted free list, enumerator noted any correct answers mentioned	
What should be used to cut the umbilical cord?	Unprompted free list, enumerator noted any correct answers mentioned	
What should be put on the cord after delivery?	Unprompted free list, enumerator noted any correct answers mentioned	
**Infant and young child feeding (5 questions)**		Maximum possible score: 5
What are some of the benefits of breastfeeding?	Unprompted free list, enumerator noted any correct answers mentioned	Promotes mother-baby bonding; helps build immunity for child; promotes child growth, wellness;<br>Reduces expenditure on medical care as child will fall sick less; the more the child breastfeeds, the more milk will be produced; it helps to maintain space between two children
How soon after delivery should you breastfeed your child?	Enumerator noted "immediately", number of hours or days mentioned, or "don't know"	Immediately or <1 hour

For peer review only

Questions	Answer options	Correct answer(s)
How many times per day should newborn babies be breastfed?	Enumerator can note if respondent provides either of the correct answers or "don't know"	9-10 times in 24 hours; On-demand (as often as the baby wants to feed)
How soon after delivery should you give foods or liquids other than mother's milk?		Six months
What types of foods should a baby be given after 6 months of age?	Unprompted free list, enumerator noted any correct answers mentioned	
**Infant and young child care (5 questions)**		Maximum possible score: 5
What danger signs do you know about for the newborn after delivery that would cause you to take the baby to the health facility immediately?	Unprompted free list, enumerator noted any correct answers mentioned	
How soon after your baby is born should it receive its first vaccination?		
What should you give your child to treat diarrhea?	Unprompted free list, enumerator noted any correct answers mentioned	
What are things you can do to prevent your child from getting diarrhea?	Unprompted free list, enumerator noted any correct answers mentioned	
What are three critical times for a woman to wash her hands?	Unprompted free list, enumerator noted any correct answers mentioned	
**Family planning (9 questions)**		Maximum possible score: 9
How soon after you give birth can you get pregnant again?		
What is the recommended length of time you should wait between having this child and getting pregnant with another child?		
What are benefits of using family planning?	Unprompted free list, enumerator noted any correct answers mentioned	
Which ways or methods contraception have you heard about?	Unprompted free list, enumerator noted any correct answers mentioned	
True/False: There are many safe methods of birth control	True / false / don't know	True
True/False: Female sterilization can be done at the time of giving birth	True / false / don't know	True
True/False: Male sterilization is an easy way to control family size	True / false / don't know	True
True/False: Men become physically weak after accepting male sterilization	True / false / don't know	False
True/False: PPIUCD insertion and female sterilization services can be free of cost at government facilities	True / false / don't know	True

For peer review only

Supplementary Table 2: Factors associated with pregnant women's RMNCH&N knowledge by domain in four districts of Madhya Pradesh, India

Variable	Mean composite score	Maternal nutrition and pregnancy danger signs	Family Planning	Infant and young child care	Immediate post-birth care for newborns	Infant and young child feeding
**Age**						
18-24	Reference					
25-34	1.91(1.35-2.46)*	1.95(1.02-2.87)*	1.92(1.18-2.67)*	1.85(1.11-2.58)*	2.32(1.1-3.54)*	1.51(0.55-2.47)*
35+	2.04(0.34-3.74)*	2.19(-0.52-4.9)	4.67(2.06-7.29)*	0.81(-1.33-2.95)	1.53(-2.42-5.47)	1.01(-2.36-4.38)
**Parity (>=1 child)**	4.39(3.74-5.03)*	3.29(2.33-4.26)*	1.74(0.92-2.56)*	5.93(5.13-6.74)*	6.8(5.46-8.14)*	4.18(3.11-5.25)*
**Caste**						
General	Reference					
Other backwards	0.62(-0.06-1.3)	0.51(-0.63-1.65)	0.07(-0.83-0.96)	0.82(-0.04-1.68)	1.94(0.47-3.41)*	-0.25(-1.41-0.91)
Scheduled	0.24(-0.58-1.06)	0.12(-1.2-1.43)	0.35(-0.71-1.4)	-0.04(-1.05-0.96)	1.08(-0.63-2.79)	-0.31(-1.65-1.04)
**Wealth Index**						
Q1	Reference					
Q2	0.32(-0.49-1.13)	-0.61(-1.99-0.78)	1.33(0.23-2.43)*	0.67(-0.4-1.75)	0.66(-1.01-2.33)	-0.47(-1.85-0.9)
Q3	0.14(-0.78-1.07)	0.17(-1.37-1.71)	1.17(-0.04-2.37)	0.5(-0.69-1.7)	-1.24(-3.06-0.57)	0.11(-1.44-1.66)
Q4	0.16(-0.83-1.16)	-0.02(-1.65-1.61)	0.95(-0.33-2.24)	0.85(-0.41-2.1)	-2.32(-4.28--0.36)*	1.35(-0.3-2.99)
Q5	0.72(-0.38-1.82)	1.8(-0.01-3.6)	2.32(0.93-3.72)*	1.21(-0.19-2.62)	-2.95(-5.22--0.68)*	1.24(-0.55-3.03)
**Education**						
No School	Reference					
Primary or Less	0.65(-0.35-1.65)	1.87(0.24-3.5)*	1.57(0.2-2.95)*	0.01(-1.34-1.36)	-1.55(-3.6-0.5)	1.35(-0.43-3.13)
Middle School	0.6(-0.51-1.72)	1.19(-0.56-2.93)	2.43(0.92-3.94)*	0.14(-1.27-1.54)	-1.78(-4.06-0.51)	1.04(-0.83-2.91)
Higher Education	1.65(0.42-2.89)*	3.42(1.51-5.34)*	3.29(1.63-4.94)*	1.5(-0.03-3.03)	-2.82(-5.41--0.24)*	2.87(0.83-4.91)*
**Literacy**						
Cannot read at all	Reference					
Reading only parts of sentence	0.95(0.01-1.9)*	0.93(-0.57-2.42)	0.88(-0.42-2.18)	0.91(-0.33-2.15)	0.33(-1.64-2.31)	1.7(0.05-3.34)*
Read whole sentence	2.29(1.42-3.15)*	3.12(1.78-4.46)*	2.29(1.17-3.41)*	1.4(0.33-2.47)*	1.86(0.11-3.61)*	2.76(1.32-4.19)*
**Self Help Group**	0.4(-0.42-1.22)	0.88(-0.57-2.34)	0.77(-0.46-2.01)	0.45(-0.71-1.61)	-0.18(-2.33-1.98)	0.08(-1.39-1.54)
**Nuclear Family**	-2.14(-2.95--1.33)*	-2.61(-3.83--1.38)*	-1.7(-2.65--0.75)*	-4.38(-5.39--3.38)*	-1.46(-2.92-0)	-0.57(-1.81-0.67)

For peer review only - http://bmjopen.bmj.com/site/about/guidelines.xhtml

**Primary decision maker in health decisions**	1.59(1-2.19)*	2.74(1.75-3.74)*	0.48(-0.32-1.27)	2.61(1.88-3.34)*	0.43(-0.8-1.66)	1.71(0.68-2.74)*
**Number of ANC Visits**						
1	Reference					
2	1.03(0.16-1.9)*	4.19(2.8-5.59)*	0.62(-0.62-1.86)	-0.03(-1.15-1.08)	0.18(-1.59-1.95)	0.17(-1.24-1.58)
3	1.95(1.04-2.85)*	5.91(4.37-7.45)*	0.35(-0.94-1.63)	0.65(-0.51-1.81)	1.38(-0.49-3.25)	1.44(-0.05-2.93)
4	1.68(0.81-2.54)*	6.61(5.13-8.1)*	0.19(-1.01-1.4)	0.43(-0.67-1.54)	0.12(-1.67-1.9)	1.02(-0.42-2.46)
**Satisfied with the services provided by the ASHA**	2.18(1.57-2.79)*	2.78(1.83-3.72)*	2.06(1.24-2.87)*	1.7(0.9-2.5)*	2.93(1.72-4.13)*	1.44(0.42-2.46)*
**Phone Ownership**	0.61(-0.01-1.22)	0.82(-0.2-1.85)	0.71(-0.11-1.52)	0.73(-0.1-1.57)	0.4(-0.82-1.63)	0.37(-0.67-1.4)
**District**						
Hoshangabad	Reference					
Mandsaur	-3.3(-4.51--2.09)*	-1.72(-3.54-0.1)	-1.49(-2.98-0)	-2.14(-3.59--0.7)*	-6.98(-9.75--4.21)*	-4.18(-5.98--2.38)*
Rewa	-0.13(-1.21-0.95)	-3.05(-4.78--1.31)*	-0.85(-2.24-0.54)	-0.69(-2.02-0.63)	3.58(1.18-5.98)*	0.36(-1.31-2.03)
Rajgarh	-3.05(-4.21--1.89)*	-6.59(-8.41--4.78)*	-1.26(-2.76-0.24)	-0.26(-1.68-1.16)	-4.35(-6.92--1.78)*	-2.77(-4.53--1.01)*

*= Significant at 0.05 level

For peer review only
